# Supplementary material for: Swedish nationwide time series analysis of influenza and suicide deaths from 1910 to 1978
Source: BMJ Open. 2021 Jul 7;11(7):e049302. doi: 10.1136/bmjopen-2021-049302 (PMC8266430; doi:10.1136/bmjopen-2021-049302)
Supplement: Supplementary data [file bmjopen-2021-049302supp001.pdf]

Supplementary material

eTable 1. Original data on the number of deaths by influenza and suicide, and population size for total population, men, and women in 1910-1978, retrieved from the Statistical Yearbook of Sweden from 1910-1978

Year	Total population, N	Men, N	Women, N	Deaths by influenza in total population, N	Deaths by influenza among men, N	Deaths by influenza among women, N	Deaths by suicide in total population, N	Deaths by suicide among men, N	Deaths by suicide among women, N
1910	5522403	2698729	2823674	534	212	322	980	787	193
1911	5561799	2718638	2843161	312	134	178	974	790	184
1912	5604192	2740737	2863455	245	101	144	1019	836	183
1913	5638583	2756946	2881637	307	139	168	1006	820	186
1914	5679607	2777447	2902160	300	128	172	899	728	171
1915	5712740	2794552	2918188	1018	417	601	876	690	186
1916	5757566	2817950	2939616	314	143	171	757	586	171
1917	5800847	2841554	2959293	213	91	122	587	444	143
1918	5813850	2849205	2964645	27379	14245	13134	580	432	148
1919	5847037	2868395	2978642	7341	3667	3674	791	623	168
1920	5904489	2898256	3006233	2853	1596	1257	866	682	184
1921	5954316	2925988	3028328	479	241	238	910	721	189
1922	5987520	2944031	3043489	2235	918	1317	861	689	172
1923	6005759	2948508	3057251	224	106	118	851	689	162
1924	6036118	2964230	3071888	458	210	248	874	705	169
1925	6053562	2972554	3081008	669	280	389	817	646	171
1926	6074368	2982625	3091743	731	335	396	896	734	162
1927	6087923	2990205	3097718	2875	1277	1598	921	736	185
1928	6105190	2999562	3105628	444	207	237	855	703	152
1929	6120080	3007946	3112134	1345	573	772	944	761	183
1930	6142191	3020848	3121343	319	146	173	970	777	193
1931	6162446	3037064	3125382	2518	1025	1493	1015	804	211
1932	6190364	3053528	3136836	531	228	303	1094	910	184
1933	6211566	3066888	3144678	673	300	373	1063	852	211
1934	6233090	3079690	3153400	303	133	170	953	777	176
1935	6250506	3090451	3160055	685	316	369	969	768	201
1936	6266888	3100534	3166354	646	275	371	1045	825	220
1937	6284722	3111256	3173466	1173	522	651	978	747	231
1938	6310214	3125000	3185214	190	95	95	996	779	217
1939	6341303	3142356	3198947	740	319	421	1022	800	222
1940	6371432	3160128	3211304	318	142	176	1086	858	228
1941	6406474	3180535	3225939	1082	434	648	1009	784	225
1942	6458200	3207756	3250444	96	43	53	922	697	225
1943	6522827	3240631	3282196	204	95	109	982	719	263
1944	6597348	3279723	3317625	172	83	89	858	671	187
1945	6673749	3321502	3352247	70	22	48	1018	761	257
1946	6763685	3366694	3396991	291	111	180	1044	777	267
1947	6842046	3407577	3434469	316	133	183	1008	770	238

Rück C, *et al. BMJ Open* 2022; 11:e049302. doi: 10.1136/bmjopen-2021-049302

Supplementary material

1948	6924888	3448122	3476766	25	8	17	1000	774	226
1949	6986181	3479079	3507102	54	20	34	1130	814	316
1950	7041829	3506442	3535387	80	44	36	1043	799	244
1951	7098740	3535736	3563004	522	208	314	1145	879	266
1952	7150606	3562475	3588131	112	45	67	1192	934	258
1953	7192316	3583598	3608718	347	145	202	1332	1008	324
1954	7234664	3605013	3629651	553	248	305	1225	918	307
1955	7290112	3633983	3656129	74	32	42	1293	983	310
1956	7338991	3659917	3679074	64	31	33	1474	1137	337
1957	7388611	3685654	3702957	649	327	322	1463	1145	318
1958	7429675	3706039	3723636	297	137	160	1282	959	323
1959	7462823	3722867	3739956	184	79	105	1350	1012	338
1960	7497967	3740119	3757848	801	378	423	1305	981	324
1961	7542028	3763040	3778988	98	47	51	1273	960	313
1962	7581148	3782252	3798896	154	71	83	1396	1043	353
1963	7627507	3805699	3821808	319	160	159	1406	1028	378
1964	7695200	3840897	3854303	39	12	27	1514	1096	418
1965	7772506	3882473	3890033	243	124	119	1459	1068	391
1966	7843088	3919170	3923918	157	68	89	1566	1145	421
1967	7892774	3942223	3950551	156	64	92	1702	1254	448
1968	7934996	3961414	3973582	291	108	183	1702	1240	462

Rück C, *et al. BMJ Open* 2022; 11:e049302. doi: 10.1136/bmjopen-2021-049302

Supplementary material

Supplementary material. Model diagnostics

We carried out several types of model diagnostics, including Portmanteau test for white noise to check autocorrelation in residuals, Breusch-Pagan/Cook-Weisberg test for heteroscedasticity, Jarque-Bera test on normality, and the regression specification error tests that indicates whether there is a misspecification in the model. We also performed tests for stability of the models by using the cumulative sum of recursive residuals and their squares to test whether there is a structural break due to changes in regression coefficients over time.

Supplementary material. STATA codes for the non-linear autoregressive distributed lag (NARDL) modelling of association between between influenza death rates and suicide rates

```
clear
use "Z:\Influenza_deaths_suicides\ALL19101978.dta"


///// 1. DATA MANAGEMENT: generate and label variables to be used in the analyses


/// Labels for original variables (retrieved from the Statistical Yearbooks 1910-1978)

 lab var year "Years 1910-1978"

 lab var number_population_all "Total number of inhabitants (population size) in Sweden in corresponding year"
 lab var number_population_men "Total number of male population in Sweden in corresponding year"
 lab var number_population_women "Total number of female population in Sweden in corresponding year"

 lab var number_influenza_all "Number of deaths from influenza in total population in corresponding year"
 lab var number_influenza_men "Number of deaths from influenza among men in corresponding year"
 lab var number_influenza_women "Number of deaths from influenza among women in corresponding year"

 lab var number_suicide_all "Number of suicides in total population in corresponding year"
 lab var number_suicide_men "Number of suicides among men in corresponding year"
 lab var number_suicide_women "Number of suicides among women in corresponding year"



/// Calculation of annual influenza death rates and suicide rates per 100,000 for total population, men, and women

 gen influenza_rates_all = (number_influenza_all/number_population_all)*100000
 gen influenza_rates_men = (number_influenza_men/number_population_men)*100000
 gen influenza_rates_women = (number_influenza_women/number_population_women)*100000

 gen suicide_rates_all = (number_suicide_all/number_population_all)*100000
 gen suicide_rates_men = (number_suicide_men/number_population_men)*100000
 gen suicide_rates_women = (number_suicide_women/number_population_women)*100000

 lab var influenza_rates_all "Influenza death rates for total population per 100,000, annual"
 lab var influenza_rates_men "Influenza death rates among men per 100,000, annual"
 lab var influenza_rates_women "Influenza death rates among women per 100,000, annual"

 lab var suicide_rates_all "Suicide rates for total population per 100,000, annual"
 lab var suicide_rates_men "Suicide rates among men per 100,000, annual"
 lab var suicide_rates_women "Suicide rates among women per 100,000, annual"



/// Logarithmic transformation for suicide rates for total population, men, and women
```


Rück C, *et al. BMJ Open* 2022; 11:e049302. doi: 10.1136/bmjopen-2021-049302

Supplementary material

```
gen ln_suicide_rates_all = ln(suicide_rates_all)
gen ln_suicide_rates_men = ln(suicide_rates_men)
gen ln_suicide_rates_women = ln(suicide_rates_women)

lab var ln_suicide_rates_all "Log-transformed suicide rates for total population per 100,000, annual"
lab var ln_suicide_rates_men "Log-transformed suicide rates among men per 100,000, annual"
lab var ln_suicide_rates_women "Log-transformed suicide rates among women per 100,000, annual"


/// Variables for changes in death registration in Sweden 1910-1978 (as dummy variables)

// based on the Bertillon criteria (prior to 1931)
gen registration_19101930=0
replace registration_19101930=1 if year<=1930
lab var registration_19101930 "Dummy variable for death registration in 1910-1930 (Bertillion)"

// introduced in cooperation with other Nordic countries (1931-1950)
gen registration_19311950=0
replace registration_19311950=1 if year>=1931 & year<=1950
lab var registration_19311950 "Dummy variable for death registration in 1931-1950 (new registration)"

// ICD-6 (1951-1957)
gen registration_19511957=0
replace registration_19511957=1 if year>=1951 & year<=1957
lab var registration_19511957 "Dummy variable for death registration in 1951-1957 (ICD-6)"

// ICD-7 (1958-1968)
gen registration_19581968=0
replace registration_19581968=1 if year>=1958 & year<=1968
lab var registration_19581968 "Dummy variable for death registration in 1958-1968 (ICD-7)"

// ICD-8 (1969-1978)
gen registration_1969after=0
replace registration_1969after=1 if year>=1969
lab var registration_1969after "Dummy variable for death registration in 1969 and after (ICD-8)"
//////////////////////////////////////////////////////////////////////////////////////////////////


///// 2. CHECKING VARIABLES' PROPERTIES AND TESTING THE CONDITIONS FOR MODELLING


/// Declare data to be time-series data
tsset year, yearly


/// Obtain optimal lags for each variable (for (i) augmentation in ADF and KPSS tests, and (ii) for p and q parameters in NARDL).
/// Lags obtained for influenza death rates and logarithmically-transformed suicide rates for total population, men, and women, and for time periods 1910-1978, 1918-1956, and 1957-1978
/// If AIC, HQIC, and SBIC information criteria indicated different lag orders, SBIC was used to select optimal lags

varsoc influenza_rates_all
varsoc influenza_rates_all if tin(1918, 1956)
varsoc influenza_rates_all if tin(1957, )

varsoc influenza_rates_men
```

Supplementary material

```
varsoc influenza_rates_men if tin(1918, 1956)
varsoc influenza_rates_men if tin(1957, )

varsoc influenza_rates_women
varsoc influenza_rates_women if tin(1918, 1956)
varsoc influenza_rates_women if tin(1957, )

varsoc ln_suicide_rates_all
varsoc ln_suicide_rates_all if tin(1918, 1956)
varsoc ln_suicide_rates_all if tin(1957, )

varsoc ln_suicide_rates_men
varsoc ln_suicide_rates_men if tin(1918, 1956)
varsoc ln_suicide_rates_men if tin(1957, )

varsoc ln_suicide_rates_women
varsoc ln_suicide_rates_women if tin(1918, 1956)
varsoc ln_suicide_rates_women if tin(1957, )


/// Tests for stationarity: ADF and KPSS for influenza death rates and logarithmically-transformed suicide rates variables for total population, men, and women
/// Augmentation by at least one lag was used (for suicide rates in women - by two lags according to SBIC in varsoc)

dfuller influenza_rates_all, lag(1)
dfuller influenza_rates_men, lag(1)
dfuller influenza_rates_women, lag(1)

dfuller ln_suicide_rates_all, lag(1)
dfuller ln_suicide_rates_men, lag(1)
dfuller ln_suicide_rates_women, lag(1)
dfuller ln_suicide_rates_women, lag(2)

dfuller D.influenza_rates_all, lag(1)
dfuller D.influenza_rates_men, lag(1)
dfuller D.influenza_rates_women, lag(1)

dfuller D.ln_suicide_rates_all, lag(1)
dfuller D.ln_suicide_rates_men, lag(1)
dfuller D.ln_suicide_rates_women, lag(1)
dfuller D.ln_suicide_rates_women, lag(2)

kpss influenza_rates_all, maxlag(1) notrend
kpss influenza_rates_men, maxlag(1) notrend
kpss influenza_rates_women, maxlag(1) notrend

kpss ln_suicide_rates_all, maxlag(1) notrend
kpss ln_suicide_rates_men, maxlag(1) notrend
kpss ln_suicide_rates_women, maxlag(2) notrend

kpss D.influenza_rates_all, maxlag(1) notrend
kpss D.influenza_rates_men, maxlag(1) notrend
kpss D.influenza_rates_women, maxlag(1) notrend

kpss D.ln_suicide_rates_all, maxlag(1) notrend
kpss D.ln_suicide_rates_men, maxlag(1) notrend
kpss D.ln_suicide_rates_women, maxlag(2) notrend
```

Supplementary material

///// 3. ESTIMATION OF NON-LINEAR AUTOREGRESSIVE DISTRIBUTED LAG (NARDL) MODELS: for total population, men, and women, and for time periods 1910-1978, 1918-1956, and 1957-1978
/// The dependent and independent variables are indicated in levels.
/// The covariates (i.e., changes in death registration system 1910-1978) are included with the deterministic option, but these were only kept in the models if statistically significant.
/// The optimal number of lags for dependent and independent variables (p and q parameters, respectively).
/// Since p and q parameters refer to levels, one additional lag is added to p and q to get an optimal lag length in differences (p and q must be at least 2).
/// The model provides an output for a long-term cointegration bounds test and diagnostic tests.

nardl ln_suicide_rates_all influenza_rates_all, p(2) q(2) h(69) plot bootstrap(100) level(95) residuals /* used as a final model */
nardl ln_suicide_rates_all influenza_rates_all, p(2) q(2) deterministic(registration_19101930 registration_19311950 registration_19511957 registration_19581968 registration_1969after) h(69) plot bootstrap(100) level(95) residuals

nardl ln_suicide_rates_men influenza_rates_men, p(2) q(2) h(69) plot bootstrap(100) level(95) residuals /* used as a final model */
nardl ln_suicide_rates_men influenza_rates_men, p(2) q(2) deterministic(registration_19101930 registration_19311950 registration_19511957 registration_19581968 registration_1969after) h(69) plot bootstrap(100) level(95) residuals

nardl ln_suicide_rates_women influenza_rates_women, p(3) q(2) h(69) plot bootstrap(100) level(95) residuals /* used as a final model */
nardl ln_suicide_rates_women influenza_rates_women, p(3) q(2) deterministic(registration_19101930 registration_19311950 registration_19511957 registration_19581968 registration_1969after) h(69) plot bootstrap(100) level(95) residuals

nardl ln_suicide_rates_all influenza_rates_all if tin(1918,1956), p(2) q(2) h(39) plot bootstrap(50) level(95) residuals
nardl ln_suicide_rates_men influenza_rates_men if tin(1918,1956), p(2) q(2) h(39) plot bootstrap(50) level(95) residuals
nardl ln_suicide_rates_women influenza_rates_women if tin(1918,1956), p(5) q(2) h(39) plot bootstrap(50) level(95) residuals

nardl ln_suicide_rates_all influenza_rates_all if tin(1957,), p(2) q(2) h(22) plot bootstrap(50) level(95) residuals
nardl ln_suicide_rates_men influenza_rates_men if tin(1957,), p(2) q(2) h(22) plot bootstrap(50) level(95) residuals
nardl ln_suicide_rates_women influenza_rates_women if tin(1957,), p(2) q(2) h(22) plot bootstrap(50) level(95) residuals

/* REFERENCES
For varsoc and dfuller:
StataCorp. Stata 15 Base Reference Manual. College Station, TX: Stata Press; 2017

For kpss:
Author: Christopher F Baum, Boston College, USA baum@@bc.edu
Kwiatkowski, D., Phillips, P.C.B., Schmidt, P. and Y. Shin. Testing the null hypothesis of stationarity against the alternative of a unit root: How sure are we that economic time series have a unit root? Journal of Econometrics, 54, 1992, 159-178.
Lee, D. and P. Schmidt. On the power of the KPSS test of stationarity against fractionally-integrated alternatives. Journal of Econometrics, 73, 1996, 285-302.
Schwert, G.W. Tests for Unit Roots: A Monte Carlo Investigation. Journal of Business and Economic Statistics, 7, 1989, 147-160.

For nardl:

Supplementary material

Author: Stata implementation by Marco Sunder (sunder@wifa.uni-leipzig.de). This version was created on 26jan2012.
Shin, Y., Yu, B., Greenwood-Nimmo, M. (2011): Modelling asymmetric cointegration and dynamic multipliers in a nonlinear ARDL framework. Working paper (version of November 2011), http://ssrn.com/abstract=1807745. */

end of do-file

Supplementary material

eTable 2. Unit root tests at the level and first difference by Augmented Dickey-Fuller (ADF) and Kwiatkowski-Phillips-Schmidt-Shin (KPSS) test statistics

	ADF at level		KPSS at level		Results for stationarity at the level	ADF at first difference		KPSS at first difference		Results for stationarity at the 1st difference	Order of integration
	Test statistic	p-value	Test statistic	p-value[1]		Test statistic	p-value	Test statistic	p-value[1]		
**Suicide rate (log-transformed)**											
All	-2.181	0.213	1.980	<0.001	nonstationary	-6.640	<0.001	0.068	>0.1	stationary	1
Males	-3.230	0.018	0.964	<0.001	nonstationary	-6.512	<0.001	0.053	>0.1	stationary	1
Females	-0.517	0.889	3.130	<0.001	nonstationary	-8.692	<0.001	0.104	>0.1	stationary	1
**Influenza death rate**											
All	-4.895	<0.001	0.461	>0.1	stationary	*-8.727*	*<0.001*	*0.016*	>0.1	*stationary*	0
Males	-4.856	<0.001	0.431	>0.1	stationary	*-8.642*	*<0.001*	*0.016*	>0.1	*stationary*	0
Females	-4.940	<0.001	0.491	>0.1	stationary	*-8.824*	*<0.001*	*0.015*	>0.1	*stationary*	0

Note: For the ADF test, the null hypothesis implies that the variable contains a unit root (the alternate hypothesis is that the variable is stationary), whereas for the KPSS test the null hypothesis implies that the variable is stationary (the alternate hypothesis is that there is a unit root). The results for ADF and KPSS tests for stationarity at first difference for influenza death rates are reported as explanatory (written in Italics) since the stationarity at the level has already been established (i.e., integrated of the order zero).

[1] KPSS test results do not indicate the exact p-value, but report the level of significance at which the null hypothesis is rejected (1%, 2.5%, 5%, or 10% significance level).

Abbreviations: ADF, Augmented Dickey–Fuller unit-root test; KPSS, Kwiatkowski-Phillips-Schmidt-Shin test for stationarity

Supplementary material

eTable 3. **Full specification for non-linear autoregressive distributed lag models used for the analysis of short-term and long-term relationship of suicide rates with positive and negative changes in influenza death rates among the whole population, men, and women in 1910-1978 in Sweden**

	**Whole population**		**Men**		**Women**	
	**Coefficient (95% CI)**	**p-value**	**Coefficient (95% CI)**	**p-value**	**Coefficient (95% CI)**	**p-value**
LnSuicide, lag 1	-0.08466 (-0.23940 to 0.07009)	0.278	-0.11457 (-0.30588 to 0.07674)	0.235	-0.04517 (-0.15832 to 0.06799)	0.427
Influenza +, lag 1	-0.00001 (-0.00761 to 0.00759)	0.998	-0.00150 (-0.01032 to 0.00732)	0.734	0.00508 (-0.00366 to 0.01382)	0.249
Influenza –, lag 1	-0.00018 (-0.00775 to 0.00739)	0.962	-0.00165 (-0.01044 to 0.00714)	0.708	0.00487 (-0.00383 to 0.01358)	0.267
ΔLnSuicide, lag 1	-0.14610 (-0.43113 to 0.13893)	0.309	0.029465 (-0.27204 to 0.33097)	0.846	-0.65184 (-0.91785 to -0.38582)	<0.001
ΔLnSuicide, lag 2	NA	NA	NA	NA	-0.30807 (-0.55722 to -0.05892)	0.016
ΔInfluenza +	0.00002 (-0.00036 to 0.00039)	0.931	0.00004 (-0.00034 to 0.00041)	0.854	-0.00007 (-0.00056 to 0.00041)	0.760
ΔInfluenza +, lag 1	0.00153 (-0.00285 to 0.00590)	0.488	0.00085 (-0.00347 to 0.00518)	0.694	0.00093 (-0.00482 to 0.00668)	0.747
ΔInfluenza –	0.00103 (-0.00579 to 0.00785)	0.764	-0.00192 (-0.01005 to 0.00621)	0.638	0.00780 (0.00015 to 0.01544)	0.046
ΔInfluenza –, lag 1	-0.00070 (-0.00236 to 0.00096)	0.401	-0.00038 (-0.00212 to 0.00136)	0.663	-0.000075 (-0.00289 to 0.00139)	0.488
**Long-term effect**						
Influenza +	-0.00012	0.998	-0.01314	0.745	0.11254	0.538
Influenza –	0.00211	0.962	0.01443	0.722	-0.10789	0.544
**Model diagnostics**						

Supplementary material

Q-test for autocorrelation, χ^2	40.160	0.125	35.260	0.273	31.850	0.424
Heteroscedasticity, χ^2	3.649	0.056	5.439	0.020	1.280	0.258
Normality, χ^2	2.826	0.243	2.237	0.327	2.372	0.305
RESET, F-statistics	4.656	0.006	5.473	0.023	1.013	0.394
CUSUM	Stable, no structural break	NA	Stable, no structural break	NA	Stable, no structural break	NA
CUSUMQ	Stable	NA	Stable	NA	Stable	NA
Adj. R^2	0.257	NA	0.300	NA	0.374	NA
$Wald_{SR}$, F-statistics	0.054	0.817	0.275	0.602	1.062	0.307
$Wald_{LR}$, F-statistics	1.206	0.277	1.176	0.283	0.771	0.384
**Cointegration test statistics**						
t_BDM	-1.095	NA	-1.199	NA	-0.799	NA
F_PSS	3.789	NA	3.735	NA	2.530	NA
**Critical values for F_PSS**						
5% critical values; I(0), I(1)	k=1: 5.055, 5.915<br>k=2: 3.947, 5.020	NA	k=1: 5.055, 5.915<br>k=2: 3.947, 5.020	NA	k=1: 5.055, 5.915<br>k=2: 3.947, 5.020	NA

Note: F_PSS denotes F-statistics for Pesaran/Shin/Smith bounds test with null hypothesis of no cointegration. Critical values are retrieved from Narayan PK (2005) for a sample size of n=70.[1] The null hypothesis of no cointegration is supported if F_PSS statistics is lower than 5% I(0) critical value; the null hypothesis is rejected if F_PSS statistics is greater than 5% I(1) critical value. We applied a conservative approach to considering the number of exposure variables (k), as suggested by Shin Y, et al (2014) [2] (since influenza death rates represent one exposure (k=1), while the analysis actually partitions the

Rück C, *et al. BMJ Open* 2022; 11:e049302. doi: 10.1136/bmjopen-2021-049302

Supplementary material

exposure to positive and negative changes (k=2)). In a given table, the results of F_PSS for the whole population, men, and women are lower than the reported 5% I(0) critical values that accepts a null hypothesis of no cointegration.

“LnSuicide” indicates that suicide rates were logarithmically-transformed. “Δ” signifies the series is differenced. Signs as “+” and “–” denote the exposure variable being partitioned in positive and negative changes, respectively. “NA” denotes that a certain test or test parameter is not applicable.

Number of lags used for each variable in the model are noted by “lag#”. To select the optimal number of lags to be used for choosing p and q parameters for the NARDL (i.e., numbers of lags for dependent and independent variables, respectively), we applied a varsoc command in STATA using the minimal values of Akaike Information Criterion, Schwarz’s Bayesian information criterion (SBIC), and the Hannan and Quinn information criterion information criteria. If information criteria indicated different lag orders, SBIC was used to select optimal lags. Thus, for the analysis of the whole population and men, both exposure and outcome time series were lagged once, while for analysis among women to outcome time series were lagged twice and exposure time series were lagged once.

Wald test for asymmetry in a short-term ($Wald_{SR}$) and long-term ($Wald_{LR}$) has a null hypothesis of no cointegration. Q-test for autocorrelation reports the results of Portmanteau test for white noise. Heteroscedasticity is measured by Breusch-Pagan/Cook-Weisberg test. Normality is measured by Jarque-Bera test. RESET statistics refers to the regression specification error tests. CUSUM and CUSUMQ refer to the tests of the cumulative sum of recursive residuals and their squares, respectively. “Stable, no structural break” in the results of CUSUM indicates that the model was found stable and the null hypothesis of no structural break was not rejected (same applies to “Stable” as an output for CUSUMQ).

Supplementary material

eTable 4. Full specification for non-linear autoregressive distributed lag models used for the analysis of short-term and long-term relationship of suicide rates with positive and negative changes in influenza death rates among the whole population, men, and women in 1918-1956 in Sweden

	Whole population		Men		Women	
	**Coefficient (95% CI)**	**p-value**	**Coefficient (95% CI)**	**p-value**	**Coefficient (95% CI)**	**p-value**
LnSuicide, lag 1	-0.32229 (-0.73472 to 0.09015)	0.121	-0.26417 (-0.60333 to 0.07492)	0.122	-0.36067 (-0.97384 to 0.25250)	0.238
Influenza +, lag 1	0.00003 (-0.00851 to 0.00858)	0.994	-0.00129 (-0.011384 to 0.00879)	0.795	0.00506 (-0.00682 to 0.01695)	0.390
Influenza –, lag 1	-0.00033 (-0.00869 to 0.00803)	0.936	-0.00145 (-0.01129 to 0.00840)	0.766	0.00396 (-0.00801 to 0.01593)	0.503
ΔLnSuicide, lag 1	-0.19387 (-0.59955 to 0.21181)	0.337	-0.03045 (-0.44243 to 0.38153)	0.881	-0.58189 (-1.19784 to 0.03406)	0.063
ΔLnSuicide, lag 2	NA	NA	NA	NA	-0.36148 (-0.91643 to 0.19346)	0.193
ΔLnSuicide, lag 3	NA	NA	NA	NA	-0.11520 (-0.61658 to 0.38617)	0.641
ΔLnSuicide, lag 4	NA	NA	NA	NA	0.11306 (-0.26409 to 0.49021)	0.544
ΔInfluenza +	0.00002 (-0.00077 to 0.00082)	0.955	-0.00014 (-0.00102 to 0.00075)	0.755	0.00098 (-0.00068 to 0.00263)	0.237
ΔInfluenza +, lag 1	0.00140 (-0.00347 to 0.00627)	0.561	0.00095 (-0.00389 to 0.00580)	0.691	-0.00063 (-0.00821 to 0.00696)	0.866
ΔInfluenza –	0.00087 (-0.00643 to 0.00817)	0.809	-0.00132 (-0.01001 to 0.00741)	0.760	0.00434 (-0.00595 to 0.01463)	0.394
ΔInfluenza –, lag 1	-0.00076 (-0.00256 to 0.00104)	0.396	-0.00044 (-0.00233 to 0.00145)	0.637	-0.00052 (-0.00328 to 0.00224)	0.704
**Long-term effect**						
Influenza +	0.00011	0.994	-0.00490	0.802	0.01403	0.521

Supplementary material

Influenza –	0.00103	0.936	0.00548	0.775	-0.01098	0.600
**Model diagnostics**						
Q-test for autocorrelation, χ^2	14.130	0.658	9.674	0.917	9.614	0.919
Heteroscedasticity, χ^2	0.939	0.333	0.768	0.381	5.042	0.025
Normality, χ^2	0.420	0.811	0.132	0.936	2.383	0.304
RESET, F-statistics	0.413	0.745	0.764	0.524	0.505	0.683
CUSUM	Stable, no structural break	NA	Stable, no structural break	NA	Stable, no structural break	NA
CUSUMQ	Stable	NA	Stable	NA	Stable	NA
Adj. R^2	0.309	NA	0.345	NA	0.432	NA
$Wald_{SR}$, F-statistics	0.056	0.815	0.155	0.697	0.183	0.673
$Wald_{LR}$, F-statistics	1.555	0.222	0.217	0.645	4.479	0.044
**Cointegration test statistics**						
t_BDM	-1.596	NA	-1.591	NA	-1.207	NA
F_PSS	1.009	NA	1.019	NA	1.328	NA
**Critical values for F_PSS**						
5% critical values; I(0) to I(1)	k=1: 5.260, 6.160<br>k=2: 4.133, 5.260	NA	k=1: 5.260, 6.160<br>k=2: 4.133, 5.260	NA	k=1: 5.260, 6.160<br>k=2: 4.133, 5.260	NA

Rück C, *et al. BMJ Open* 2022; 11:e049302. doi: 10.1136/bmjopen-2021-049302

Supplementary material

Note: F_PSS denotes F-statistics for Pesaran/Shin/Smith bounds test with null hypothesis of no cointegration. Critical values are retrieved from Narayan PK (2005) for a sample size of n=40 [1]. The null hypothesis of no cointegration is supported if F_PSS statistics is lower than 5% I(0) critical value; the null hypothesis is rejected if F_PSS statistics is greater than 5% I(1) critical value. We applied a conservative approach to considering the number of exposure variables (k), as suggested by Shin Y, et al (2014) [2] (since influenza death rates represent one exposure (k=1), while the analysis actually partitions the exposure to positive and negative changes (k=2)). In a given table, the results of F_PSS for the whole population, men, and women are lower than the reported 5% I(0) critical values that accepts a null hypothesis of no cointegration.

"LnSuicide" indicates that suicide rates were logarithmically-transformed. "Δ" signifies the series is differenced. Signs as "+" and "–" denote the exposure variable being partitioned in positive and negative changes, respectively. "NA" denotes that a certain test or test parameter is not applicable.

Number of lags used for each variable in the model are noted by "lag#". To select the optimal number of lags to be used for choosing p and q parameters for the NARDL (i.e., numbers of lags for dependent and independent variables, respectively), we applied a varsoc command in STATA using the minimal values of Akaike Information Criterion, Schwarz's Bayesian information criterion (SBIC), and the Hannan and Quinn information criterion information criteria. If information criteria indicated different lag orders, SBIC was used to select optimal lags. Thus, for the analysis of the whole population and men, both exposure and outcome time series were lagged once, while for analysis among women, outcome time series were lagged four times and exposure time series were lagged once.

Wald test for asymmetry in a short-term ($Wald_{SR}$) and long-term ($Wald_{LR}$) has a null hypothesis of no cointegration. Q-test for autocorrelation reports the results of Portmanteau test for white noise. Heteroscedasticity is measured by Breusch-Pagan/Cook-Weisberg test. Normality is measured by Jarque-Bera test. RESET statistics refers to the regression specification error tests. CUSUM and CUSUMQ refer to the tests of the cumulative sum of recursive residuals and their squares, respectively. "Stable, no structural break" in the results of CUSUM indicates that the model was found stable and the null hypothesis of no structural break was not rejected (same applies to "Stable" as an output for CUSUMQ).

Supplementary material

eTable 5. Full specification for non-linear autoregressive distributed lag models used for the analysis of short-term and long-term relationship of suicide rates with positive and negative changes in influenza death rates among the whole population, men, and women in 1957-1978 in Sweden

	Whole population		Men		Women	
	**Coefficient (95% CI)**	**p-value**	**Coefficient (95% CI)**	**p-value**	**Coefficient (95% CI)**	**p-value**
LnSuicide, lag 1	-0.64009 (-1.15104 to -0.12914)	0.018	-0.52238 (-1.11901 to 0.07425)	0.081	-0.66040 (-1.04522 to -0.27558)	0.003
Influenza +, lag 1	0.008034 (-0.02823 to 0.04429)	0.640	-0.01385 (-0.05324 to 0.02553)	0.461	0.02702 (-0.00364 to 0.05768)	0.079
Influenza –, lag 1	0.00661 (-0.02823 to 0.04429)	0.699	-0.01281 (-0.054045 to 0.02841)	0.514	0.01895 (-0.01066 to 0.04857)	0.190
ΔLnSuicide, lag 1	0.14293 (-0.41621 to 0.70206)	0.590	-0.04565 (-0.70111 to 0.60981)	0.883	0.16936 (-0.25995 to 0.59868)	0.409
ΔInfluenza +	-0.00964 (-0.02252 to 0.00324)	0.883	-0.00876 (-0.02738 to 0.00985)	0.328	-0.00976 (-0.02033 to 0.00081)	0.068
ΔInfluenza +, lag 1	-0.01999 (-0.04573 to 0.00574)	0.117	-0.01837 (-0.04871 to 0.01197)	0.214	-0.01298 (-0.04073 to 0.014772)	0.331
ΔInfluenza –	0.00194 (-0.02614 to 0.03003)	0.130	-0.01531 (-0.04658 to 0.01596)	0.309	0.02155 (-0.00343 to 0.04653)	0.085
ΔInfluenza –, lag 1	0.00864 (-0.00271 to 0.02000)	0.124	0.01033 (-0.00294 to 0.02359)	0.116	0.00585 (-0.00565 to 0.01735)	0.292
**Long-term effect**						
Influenza +	0.01255	0.618	-0.02659	0.541	0.04091	0.064
Influenza –	-0.01033	0.684	0.024538	0.582	-0.02870	0.184
**Model diagnostics**						
Q-test for autocorrelation, χ^2	5.662	0.773	3.790	0.925	5.243	0.813

Supplementary material

Heteroscedasticity, χ^2	0.102	0.750	0.155	0.694	0.079	0.778
Normality, χ^2	1.569	0.456	1.627	0.443	1.237	0.537
RESET, F-statistics	5.839	0.014	3.348	0.064	1.089	0.398
CUSUM	Stable, no structural break	NA	Stable, no structural break	NA	Stable, no structural break	NA
CUSUMQ	Stable	NA	Stable	NA	Stable	NA
Adj. R^2	0.246	NA	0.337	NA	0.371	NA
$Wald_{SR}$, F-statistics	2.903	0.112	0.536	0.477	7.132	0.019
$Wald_{LR}$, F-statistics	0.922	0.354	0.297	0.595	26.74	<0.001
**Cointegration test statistics**						
t_BDM	-2.706	NA	-1.891	NA	-3.707	NA
F_PSS	2.649	NA	2.075	NA	4.688	NA
**Critical values for F_PSS**						
5% critical values; I(0), I(1)	k=1: 5.395, 6.350<br>k=2: 4.267, 5.473	NA	k=1: 5.395, 6.350<br>k=2: 4.267, 5.473	NA	k=1: 5.395, 6.350<br>k=2: 4.267, 5.473	NA

Note: F_PSS denotes F-statistics for Pesaran/Shin/Smith bounds test with null hypothesis of no cointegration. Critical values are retrieved from Narayan PK (2005) for a sample size of n=30 [1]. The null hypothesis of no cointegration is supported if F_PSS statistics is lower than 5% I(0) critical value; the null hypothesis is rejected if F_PSS statistics is greater than 5% I(1) critical value. We applied a conservative approach to considering the number of exposure variables (k), as suggested by Shin Y, et al (2014) [2] (since influenza death rates represent one exposure (k=1), while the analysis actually partitions the exposure to positive and negative changes (k=2)). In a given table, the results of F_PSS for the whole population, men, and women are lower than the reported 5% I(0) critical values that accepts a null hypothesis of no cointegration.

Supplementary material

"LnSuicide" indicates that suicide rates were logarithmically-transformed. "Δ" signifies the series is differenced. Signs as "+" and "–" denote the exposure variable being partitioned in positive and negative changes, respectively. "NA" denotes that a certain test or test parameter is not applicable.

Number of lags used for each variable in the model are noted by "lag#". To select the optimal number of lags to be used for choosing p and q parameters for the NARDL (i.e., numbers of lags for dependent and independent variables, respectively), we applied a varsoc command in STATA using the minimal values of Akaike Information Criterion, Schwarz's Bayesian information criterion (SBIC), and the Hannan and Quinn information criterion information criteria. If information criteria indicated different lag orders, SBIC was used to select optimal lags. Thus, for the analysis of the whole population, men, and women, both exposure and outcome time series were lagged once.

Wald test for asymmetry in a short-term ($Wald_{SR}$) and long-term ($Wald_{LR}$) has a null hypothesis of no cointegration. Q-test for autocorrelation reports the results of Portmanteau test for white noise. Heteroscedasticity is measured by Breusch-Pagan/Cook-Weisberg test. Normality is measured by Jarque-Bera test. RESET statistics refers to the regression specification error tests. CUSUM and CUSUMQ refer to the tests of the cumulative sum of recursive residuals and their squares, respectively. "Stable, no structural break" in the results of CUSUM indicates that the model was found stable and the null hypothesis of no structural break was not rejected (same applies to "Stable" as an output for CUSUMQ).

REFERENCES

1 Narayan PK. The saving and investment nexus for China: evidence from cointegration tests. *Applied Economics* 2005; **37**(17): 1979–1990.
2 Shin Y, Yu B, Greenwood-Nimmo M. Modelling Asymmetric Cointegration and Dynamic Multipliers in a Nonlinear ARDL Framework. In: Sickles RC, Horrace WC, eds. Festschrift in Honor of Peter Schmidt: Econometric Methods and Applications. New York: Springer Science+Business Media, 2014: 281–314.

Rück C, *et al. BMJ Open* 2022; 11:e049302. doi: 10.1136/bmjopen-2021-049302
