# Supplementary material for: Swedish nationwide time series analysis of influenza and suicide deaths from 1910 to 1978
Source: BMJ Open. 2021 Jul 7;11(7):e049302. doi: 10.1136/bmjopen-2021-049302 (PMC8266430; doi:10.1136/bmjopen-2021-049302)
Supplement: Author's manuscript [file bmjopen-2021-049302.draft_revisions.pdf]

BMJ Open

BMJ Open is committed to open peer review. As part of this commitment we make the peer review history of every article we publish publicly available.

When an article is published we post the peer reviewers' comments and the authors' responses online. We also post the versions of the paper that were used during peer review. These are the versions that the peer review comments apply to.

The versions of the paper that follow are the versions that were submitted during the peer review process. They are not the versions of record or the final published versions. They should not be cited or distributed as the published version of this manuscript.

BMJ Open is an open access journal and the full, final, typeset and author-corrected version of record of the manuscript is available on our site with no access controls, subscription charges or pay-per-view fees (http://bmjopen.bmj.com).

If you have any questions on BMJ Open's open peer review process please email info.bmjopen@bmj.com

BMJ Open

Do pandemics lead to an increase in suicides? A Swedish nationwide analysis of historical and 2020 data

Journal:	*BMJ Open*
Manuscript ID	bmjopen-2021-049302
Article Type:	Original research
Date Submitted by the Author:	22-Jan-2021
Complete List of Authors:	Rück, Christian; Karolinska Institutet, Department of Clinical Neuroscience, Center for Psychiatry Research<br>Mataix-Cols, David; Karolinska Institutet, Department of Clinical Neuroscience, Center for Psychiatry Research<br>Malki, Kinda; Karolinska Institutet<br>Adler, Mats; Karolinska Institutet<br>Flygare, Oskar; Karolinska Institutet<br>Runeson, Bo; Karolinska Institutet, Clin Neuroscience<br>Sidorchuk, Anna; Karolinska Institutet Department of Clinical Neuroscience,
Keywords:	MENTAL HEALTH, COVID-19, Suicide & self-harm < PSYCHIATRY

SCHOLARONE™
Manuscripts

I, the Submitting Author has the right to grant and does grant on behalf of all authors of the Work (as defined in the below author licence), an exclusive licence and/or a non-exclusive licence for contributions from authors who are: i) UK Crown employees; ii) where BMJ has agreed a CC-BY licence shall apply, and/or iii) in accordance with the terms applicable for US Federal Government officers or employees acting as part of their official duties; on a worldwide, perpetual, irrevocable, royalty-free basis to BMJ Publishing Group Ltd ("BMJ") its licensees and where the relevant Journal is co-owned by BMJ to the co-owners of the Journal, to publish the Work in this journal and any other BMJ products and to exploit all rights, as set out in our licence.

The Submitting Author accepts and understands that any supply made under these terms is made by BMJ to the Submitting Author unless you are acting as an employee on behalf of your employer or a postgraduate student of an affiliated institution which is paying any applicable article publishing charge ("APC") for Open Access articles. Where the Submitting Author wishes to make the Work available on an Open Access basis (and intends to pay the relevant APC), the terms of reuse of such Open Access shall be governed by a Creative Commons licence – details of these licences and which Creative Commons licence will apply to this Work are set out in our licence referred to above.

Other than as permitted in any relevant BMJ Author's Self Archiving Policies, I confirm this Work has not been accepted for publication elsewhere, is not being considered for publication elsewhere and does not duplicate material already published. I confirm all authors consent to publication of this Work and authorise the granting of this licence.

For peer review only

Word count

Abstract: 293

Body: 3405

Tables: 3

Figures: 3

Do pandemics lead to an increase in suicides? A Swedish nationwide analysis of historical and 2020 data

Christian Rück, M.D.[1], David Mataix-Cols, Ph.D.[1], Kinda Malki, M.D. [1], Mats Adler, M.D. [1], Oskar Flygare, M.Sc.[1], Bo Runeson, M.D. [1], Anna Sidorchuk, M.D.[1]

Corresponding author:
Professor Christian Rück, M46, Karolinska University Hospital Huddinge, SE-14186 Huddinge, Sweden E-mail: Christian.ruck@ki.se, telephone +46704843392.

Affiliation:
1. Centre for Psychiatry Research, Department of Clinical Neuroscience, Karolinska Institutet, & Stockholm Health Care Services, Region Stockholm, Sweden

For peer review only

ABSTRACT

Objectives: There is concern that the COVID-19 pandemic will lead to a surge of suicides, but evidence supporting a link between pandemics and suicide is limited. Using data from the three influenza pandemics of the 20th century, we aimed to investigate whether an association exists between influenza deaths and suicide deaths.

Design: Time series analysis.

Setting: Sweden.

Participants: Deaths from influenza and suicides extracted from the Statistical Yearbook of Sweden for 1910-1978, covering three pandemics, and from the National Board of Health and Welfare for January to June of each year during 2000-2020, and deaths from COVID-19 for the first half of 2020.

Main outcome measures: Annual suicide rates in Sweden among the whole population, men, and women. Non-linear autoregressive distributed lag (NARDL) models was implemented to explore if there is a short-term and/or long-term effect of increases and decreases in influenza death rates on suicide rates during 1910-1978. Descriptive analyses were used for 2020 data.

Results: Between 1910-1978, there was no evidence of either short-term or long-term significant associations between influenza death rates and changes in suicides (β-coefficients of 0.00002, P=0.931 and β=0.00103, P=0.764 for short-term effect of increases and decreases in influenza death rates, respecively, on suicide rates, and β=-0.0002, P=0.998 and β=0.00211, P=0.962 for long-term effect of increases and decreases in influenza death rates, respecively, on suicide rates). The same pattern emerged in separate analyses for men and women. Suicide rates in January-June 2020 revealed a slight decrease compared to corresponding rates in January-June 2019 (-1.2% among men and -12.8% among women).

Conclusions: We found no evidence of short or long-term association between influenza death rates and suicide death rates across three 20th century pandemics and the first wave of COVID-19 did not lead to an acute increase of suicides.

Key words: pandemics, suicide, mental health, Spanish flu, Asian influenza, Hong-Kong influenza, COVID-19

Strengths and limitations of this study

- First study to assess influenza death and suicide across several pandemics.
- The large amount of nationwide data on influenza and suicide death rates covering 1910-1978 complemented with the most recent official information, including deaths due to COVID-19, is a strength of the study.
- To guard against effects of changes in the recording of causes of death, we created a series of dummy variables for each corresponding period but no significant effects were found.
- No historical data with higher temporal resolution than yearly data could affect the results if the time sequence of association between changes in influenza death and suicide differ from the chosen time interval.

For peer review only - http://bmjopen.bmj.com/site/about/guidelines.xhtml

INTRODUCTION

Various international surveys have documented a negative impact of the COVID-19 pandemic on the population's mental health, with increased levels of psychological stress, psychiatric symptoms, insomnia, and alcohol consumption.[1–4] A report from the UK also indicated an increase in suicidal ideation.[3] Whether these acute impacts will persist long term is currently unknown. The Royal College of Psychiatrists in the UK has warned of a "tsunami of mental illness", and the World Health Organization as well as the International Academy of Suicide Research have raised concerns of a possible increase in suicide rates.[5–7] Such concerns originate from a combination of known risk factors for suicide, including the impacts of social distancing and disconnectedness, an economic downturn, the decreased access to mental health services, and increased access to lethal means exemplified by an increase in gun purchases in the US.[8] For example, a study of the economic recession in USA 2007-2009 found that for every percentage point increase in the unemployment rate, there was about a 1.6 per cent increase in the suicide rate.[9,10] By contrast, a study failed to find an increase in suicide rates in Sweden during the two most recent economic recessions.[11]

While the concern is widespread, there is currently little evidence to support a clear association between the ongoing COVID-19 outbreak and an increased risk of suicide. A recent review concluded that the quality of the evidence so far on the association between COVID-19 and suicidal behaviour was low and initial reports do not indicate an acute increase of suicides in the first COVID-19 wave.[12,13] Historic US mortality data from the largest pandemic in the 20th century, the Spanish flu, showed that the Spanish flu was associated with an increase in suicides but those effects may have been mitigated by a decline in alcohol consumption.[14] The outbreak of the Severe Acute Respiratory Syndrome (SARS) epidemic in 2003 in Hong Kong was according to two studies associated with an increase of suicide in the population above the age of 65.[15,16] In the first of these two reports, the association was only statistically significant in elderly women.[15] To summarize, at present, our understanding of the effects of pandemics on suicide rates is very limited.

The availability of high-quality historical data on mortality due to influenza and suicide in Sweden provides a unique opportunity to examine this important question. Using historical data from the three major influenza pandemics of the 20th century, we aimed to formally investigate whether an association between influenza deaths and suicide deaths exists. Given the substantial sex differences in suicidal behaviour, we additionally report on associations separately in men and women. We also report preliminary nationwide data from the first half of 2020.

METHODS

Data acquisition

Annual data on influenza death rates and suicide rates were estimated based on information from the Statistical Yearbook of Sweden from 1910-1978.[17] Over this period, Sweden was hit by three influenza pandemics which ocurred during different socio-political contexts, namely the Spanish flu (1918-1920, with the first case appearing in Sweden in June 1918), the Asian flu (1957-1958, being first documented in August 1957), and the Hong Kong flu (1968-1969, starting in the autumn of 1968).[18] The yearbooks were produced by Statistics

For peer review only - http://bmjopen.bmj.com/site/about/guidelines.xhtml

Sweden, a governmental agency responsible for the official statistics in Sweden, with a history of population statistics going back to the 18th century.[17] For each year from 1910 to 1978, we retrieved information on the total population of Sweden, the number of deaths by influenza (if death cause was indicated as "influenza") and the number of suicides (if death cause was indicated as "suicide"), as well as the corresponding data separately for men and women. Since yearbooks reported data retrospectively for several years prior to the year each book was published, we checked the correctness of retrieved data by comparing yearbooks with overlapping reporting periods. We constructed influenza mortality rates and suicide rates per 100 000 inhabitants for each year by dividing the number of deaths by influenza and, separately, suicide, by the total number of individuals registered in Sweden in a corresponding year and then multiplying by 100 000. The corresponding rates for men and women were constructed likewise. In addition, we collected information on the changes in registration of deaths in Sweden that included the cause of death classification based on the Bertillon criteria (prior to 1931), the new classification introduced in cooperation with statistical authorities from other Nordic countries (1931-1950), the International Classification of Diseases (ICD) Sixth Revision (ICD-6; 1951-1957), ICD-7 (1958-1968), and ICD-8 (1969-1978).[19] To capture a potential effect of changes in classification, we created a series of dummy variables for each corresponding period, but these were only kept in the models if statistically significant.

On November 17th, 2020, the Swedish National Board of Health and Welfare published statistics on the number all-cause and cause specific deaths (for 1997-2020) and the corresponding age-standardized death rates per 100 000 inhabitants for men and women (for 2000-2020; with the total population of Sweden in 2019 as the standard population for both men and women).[20] Importantly, all reported mortality data refer to deaths that occurred during January-June of each year from 1997 to 2020. The reported statistics also included deaths due to COVID-19 that occurred during the first six months of 2020. As the data were made publicly available, we retrieved information on January-June 2000-2020 age-standardized death rates due to influenza (ICD-10 codes: J09 – J11) and suicide and other intentional self-harm (ICD-10 codes: X60 – X84, Y870), and information on January-June 2020 age-standardized death rates due to COVID-19 (ICD-10 codes: U07.1-U07.2), in men and women.

The study protocol was not preregistered. The full dataset used in the analysis of historical data (1910-1978) and the STATA code are available in the online Supplement (**Supplementary eTable 1** and **Supplementary material**). Data on deaths in January-June 1997-2020 are available online at the National Board of Health and Welfare.[20]

Statistical Analyses

We used the historical data 1910-1978 that span over three pandemics and focused on exploring a possible asymmetric short-term effect (i.e., instantaneous) and long-term effect (i.e., if distributed over a longer period of time) of influenza death rates on suicides by applying non-linear autoregressive distributed lag (NARDL) models,[21] a technique initially introduced for research in economics. NARDL models split the exposure in partial sum of positive changes (i.e, increases in influenza death rates) and partial sum of negative changes (i.e., decreases in influenza death rates) and explore if an outcome responds differently (i.e., asymmetrically) to an increase and decrease in exposure variable.[21] In other words, NARDL models do not rely on an assumption of symmetrical effects, by which the association of the outcome with a unit of positive change in the exposure is expected to be equal in strength and opposite in direction to the association between the outcome and a unit

For peer review only - http://bmjopen.bmj.com/site/about/guidelines.xhtml

of negative change in the exposure. We performed the modelling by using the *nardl* command in STATA.

Before the NARDL model is executed, it is important to test that the conditions for the modelling are fulfilled. It starts from testing whether or not the variables are stationary (i.e., their means and variances are constant over time). The advantage of the NARDL modelling is that it can be used regardless of whether the variables are integrated of the order zero, which means that variables are stationary, integrated of the the order one, which indicates non-stationarity, or mixed.[21] We examined the stationarity properties of the variables by using the Augmented Dickey–Fuller unit-root test (ADF) and Kwiatkowski-Phillips-Schmidt-Shin test for stationarity (KPSS) to explore the order of the integration for influenza death rates and suicide rates separately for the total population, men, and women. Previous studies on suicides hypothesized that various suicidogenic factors may interplay and reinforce each other and the logarithmic transformation of suicide time series was suggested.[22,23] We made such transformation by expressing suicide rates in natural logarithm. To select the optimal number of lags (i.e., how far in the past the dependency among measurements is examined) to be used in the NARDL for dependent and independent variables, we applied the *varsoc* command in STATA using the minimal values of Akaike Information Criterion, Schwarz's Bayesian information criterion (SBIC), and the Hannan and Quinn information criterion information criteria. If information criteria indicated different lag orders, SBIC was used to select optimal lags. We applied the NARDL model to (i) estimate the coefficients and the corresponding 95% confidence intervals for short-term and long-term association of suicide rates with an increase and decrease in influenza death rates; (ii) explore if there is a long-term cointegration between exposure and outcome by using a bounds test (if variables are cointegrated, it means their positive and negative components do not drift far away from each other in the longer term); and (iii) obtain Wald test statistics that specifies whether or not short-term and/or long-term relation between the exposure and outcome is asymmetrical. Model diagnostic tests are described in the **Supplementary material**.

The modelling was performed, first, for the whole period of 1910-1978, and then for the periods of 1918-1956 (from the beginning of the Spanish influenza pandemic to the year before the Asian influenza pandemic), and for 1957-1978 (from the beginning of Asian pandemic to the end of observation period; with this interval also covering the Hong Kong influenza pandemic).

For a descriptive analysis of the data from two recent decades, we graphically visualized age-standardized death rates among men and women for influenza and suicides across January-June 2000-2020, and for COVID-19 in January-June 2020. Additionally, for suicides among men and women, we estimated the relative change in suicide rates reported for the first six months of 2020 to the rates reported for the same months in 2019.

All statistical analyses were performed using STATA version 15.1 (StataCorp LLC, College Station, TX, USA).

Patient and public involvement
Patients and members of the public were not directly involved as this study used publically available historical national mortality data.

For peer review only - http://bmjopen.bmj.com/site/about/guidelines.xhtml

RESULTS

Over the period 1910-1978, influenza death rates fluctuated considerably in Sweden with the highest rates being observed during the Spanish flu (in 1918, 1919, and 1920, the rates were 470.93, 125.55, and 48.32 per 100 000 inhabitants, respectively) (**Figure 1**). In post-pandemic years, several noticeable peaks in influenza death rates appeared, with the ones in 1922, 1927, 1929, 1931, 1937, and 1941 being particularly high. In the following years, a considerable fluctuation in influenza mortality remained, although the rates during the periods of the Asian flu (in 1957 and 1958: 8.78 and 3.99 per 100 000 inhabitants, respectively) and the Hong Kong flu (in 1968 and 1969: 3.67 and 4.68 per 100 000 inhabitants, respectively) were lower than that in the first half of the century. The influenza death rates were very similar in men and women across the entire observation period (**Figure 2**).

[Figure 1 to be inserted here]

[Figure 2 to be inserted here]

During the same period, a total of 80 058 deaths due to suicide occurred in Sweden (60 713 in men and 19 345 in women). The average suicide rate across 1910-1978 was 16.79 per 100 000 inhabitants (standard deviation [SD] of 2.58), with corresponding rates for men and women as 25.85 (SD=3.37) and 7.91 (SD=2.26) per 100 000, respectively. There was a considerable fluctuation in the suicide rates over time (**Figure 1**). The initial decrease in suicide rates during 1913-1918, with the lowest rate of 9.97 per 100 000 in 1918, was followed by an increase with the highest peak of 22.15 per 100 000 reached in 1970, with a series of intermediate peaks.

Sex-specific suicide rates differed considerably with the rates among men being between twice to over four times higher than that in women (on average, 3.4-times higher) (**Figure 2**). Moreover, suicide rates in men exhibited a sharp dip in 1913-1918, which was first followed by an increase by 1921 and then continued to raise more gradually, although with several distinct peaks and, rarely, dips. Among women, an upward trend in suicide rate was present, although with less fluctuations than that in men.

In our analysis of 1910-1978 data, the first step with the use of the ADF and KPSS test statistics on variables' stationarity indicated that the logarithmically-transformed suicide rates were integrated of the order one (for all, and men and women separately); whereas the influenza death rates were integrated of the order zero (for all and for men and women separately) (**Supplementary eTable 2)**. On this premise, we implemented the NARDL modelling. Dummy variables on changes in death registration did neither appear statistically significant nor affected any parameter estimates in the initial model, and thus were not included in the final models. As reported in **Table 1**, for the observation period of 1910-1978, there were no statistically significant associations (either short-term or long-term) between decreased or increased influenza death rates and suicide rates among the overall population and among men. The corresponding results for women indicated a possible short-term association whereby a decrease in influenza rates seemed to be associated with a borderline decrease in suicides; however, the findings were not supported by the Wald test for asymmetry ([$Wald_{SR}$], i.e. the null hypothesis of $Wald_{SR}$ that an increase and decrease in influenza death rates would symmetrically affect suicide rates was not rejected). This suggests that association was likely due to the effect of other unobserved factors that may influence suicides in women. Full specification of the models is reported in **Supplementary eTable 3**. Full specification also includes the results of testing for long-term cointegration between

For peer review only - http://bmjopen.bmj.com/site/about/guidelines.xhtml

influenza mortality and suicides (as reported by F-statistics for Pesaran/Shin/Smith bounds test [F_PSS]). Long-term cointegration was not found as we were unable to reject the null hypothesis of no cointegration between variables (**Supplementary eTable 3,** see footnotes for details on bounds test for cointegration).

The results of the additional analyses for the periods 1918-1956 and 1957-1978 also failed to provide clear evidence of association between changes in influenza mortality rates and suicide rates in either short-term or long-term. However, in the analysis among women, a long-term asymmetry was suggested in both periods (the Wald test for asymmetry in the long-term [Wald_{LR}] p=0.044 and p<0.001 in the analyses of 1918-1956 and 1957-1978, respectively) and a short-term asymmetry in the analysis of 1957-1978 (Wald_{SR} test p=0.019) (**Tables 2** and **3**). We assume that these findings may again reflect the influence of unobserved confounders, as these results were not supported by other coefficients. It is important to mention that the results for 1957-1978 should be considered with caution since the analysis included time series with only 22 observations. Full specifications for the models used in the analyses of 1918-1956 and 1957-1978 are reported in **Supplementary eTable 4** and **5**. No evidence of long-term cointegration between influenza and suicide rates were found in either period (F_PSS statistics does not reject the null hypothesis in the analyses of the whole population, men, and women; **Supplementary eTable 4** and **5,** see footnotes for details on bounds test for cointegration).

A descriptive analysis of mortality data from the National Board of Health and Welfare for January-June 2000-2020 (**Figure 3**) showed a considerable fluctuation over time in influenza death rates along with a close correspondence in those rates in men and women. For both sexes, the highest influenza death rates in January-June appeared in 2000 and 2018. During the first six months of 2020, influenza death rates were low in both men and women (1.5 and 1.0 per 100 000 inhabitants, respectively), contrasting the very high COVID-19 death rates observed during the same period (67.8 and 39.9 per 100 000 inhabitants in men and women, respectively). For suicides, data from January-June of each year from 2000 to 2020 revealed, on average, 2.5-time higher rates in men. Interestingly, the relative change in suicide rates in the first half of 2020 compared to suicide rates in the first half of 2019 showed a decrease in rates, although marginal in men (-1.2%; from 8.6 per 100 000 in January-June 2019 to 8.5 per 100 000 in January-June 2020), while more noticeable in women (-12.8%; from 3.9 per 100 000 in January-June 2019 to 3.4 per 100 000 in January-June 2020).

[Figure 3 to be inserted here]

DISCUSSION

This study used publicly available Swedish national data from 1910 to 1978 to shed light on the potential association between influenza-related deaths and over 80 000 deaths by suicide across the three 20th century pandemics. To our knowledge, this is the first study of influenza death and suicide that analyses data from several influenza pandemics. The full modelling provided no clear evidence of either a short-term or a long-term relationship between changes in influenza death rates and changes in suicide rates. The year with the highest number of influenza-related deaths by far, 1918, had the lowest number of suicides in the whole time series. Consistently, preliminary data from the COVID-19 pandemic revealed that suicide rates had slightly decreased in January-June 2020, compared to the rates in January-June 2019 (relative decrease by -1.2% among men and -12.8% among women).

The findings in context

For peer review only - http://bmjopen.bmj.com/site/about/guidelines.xhtml

International historical data on pandemics and suicide are very scarce. A report from the US focusing on the years 1910-1920 and using monthly data suggested a possible association between influenza deaths and suicide.[14] Similarly, a study focusing on SARS and suicide found some evidence of sex-specific effects in the short-term.[15] As suicide rates varied considerably in the years preceding SARS in Hong Kong, it is difficult to be certain that SARS, and not other contributing factors, was causally associated with an increase in suicides. In the present study, which uniquely spanned over several decades, we found that while suicide was consistently more prevalent among men throuought the study period, there were no sex-specific associations to influenza death.

Meaning of the study

What do these results mean for our understanding of the short- and long-term consequences of COVID-19? Our results do not support the widespread belief that global pandemics may necessarily lead to a substantial increase in suicides. There are many factors associated with a global pandemic that may potentially increase suicide risk in the population, but these may be offset by other protective factors that should not be overlooked. A shared sense of belonging and focus, social connectedness and a "pulling together effect" may be one such factor.[1,24]

Strengths and limitations

The use of a large amount of historical national data across three pandemics and up-to-date official information on influenza and suicide death rates, including recently reported death rates due to COVID-19, is the major strength of this study.

The study used the Swedish historical public death records and we had no means of verifying the causes of death. The coverage and precision of these records is likely to have improved over time for both variables. To guard against effects of changes in the recording of causes of death, we created a series of dummy variables for each corresponding period but no significant effects of those were found. If there were some other time-varying factors that could affect the coverage and precision of death records, apart from the official changes in registration system, this might have biased our results, in particular if such factors differentially affected the quality of recording deaths due to influenza and suicide. As we did not have access to data with higher temporal resolution than yearly data, that could affect our results if the time sequence of association between changes in influenza death and suicide differ from the chosen time interval. A multitude of factors may vary that impact the resilience of the society with regards to the effect of a pandemic. Such factors may also vary over time and place. However, the fact that we observed no clear associations between influenza and suicide deaths across pandemics, which challenged society with various degrees of lockdown, economic effects and health care supply issues, does support the stability of our findings.

Conclusion

In this national analysis of historical data spanning across three 20th century pandemics, we found no evidence of a short- or long-term association between influenza death rates and suicide rates. Suicide rates in January-June 2020 (when the first wave of COVID-19 occurred) were not higher than those of the corresponding period in 2019. Our results challenge the notion that a tsunami of suicides is to be expected as a result of COVID-19 and we suggest a more moderated message to the public to avoid unfounded fear and an ineffective use of resources for prevention and care.

For peer review only - http://bmjopen.bmj.com/site/about/guidelines.xhtml

Footnotes

Contributor statement: CR and AS have full access to all the data in this study and take full responsibility as guarantors for the integrity of the data and the accuracy of the data analysis. CR, DMC, and AS conceived and designed the study. CR, KM, MA, and AS performed data collection. AS undertook the statistical analysis. CR, DMC, and AS drafted the manuscript. All authors provided critical input to the analyses, interpreted the data, and revised the manuscript critically. The corresponding author confirms that all listed authors meet authorship criteria and that no others meeting the criteria have been omitted.

Funding: Karolinska Institutet and Region Stockholm, which provide salary for the authors, had no involvement in the study design; collection, analysis, and interpretation of data; in the writing of the report; or in the decision to submit the paper for publication.

Competing interests: All authors have completed the ICMJE uniform disclosure form at www.icmje.org/coi_disclosure.pdf and declare: no grant support from any organisation for the submitted work, salaries of the author were provided from Region Stockholm and Karolinska Institutet but not specifically for this project; no financial relationships with any organisations that might have an interest in the submitted work in the previous three years; no other relationships or activities that could appear to have influenced the submitted work.

Ethical approval: Since only data already available in the public domain were used, ethical approval was not necessary.

Data sharing: The full dataset (numbers of deaths by influenza and suicide and total population for 1910-1978) is published as supplementary material (eTable 1). Data on deaths in January-June 1997-2020 are available online at the National Board of Health and Welfare.[20,25]

Dissemination to participants and patient organizations: since only aggregated mortiality data was used, dissemination to participants is not possible. We intend to disseminate the results to the general public via media and the research groups webpage.

Transparency declaration: The lead author affirms that this manuscript is an honest, accurate, and transparent account of the study being reported; that no important aspects of the study have been omitted; and that any discrepancies from the study as planned have been explained.

For peer review only

References

1 Reger MA, Stanley IH, Joiner TE. Suicide Mortality and Coronavirus Disease 2019—A Perfect Storm? *JAMA Psychiatry* Published Online First: 10 April 2020. doi:10.1001/jamapsychiatry.2020.1060

2 Dutheil F, Mondillon L, Navel V. PTSD as the second tsunami of the SARS-Cov-2 pandemic. *Psychol Med* 2020;:1–2. doi:10.1017/S0033291720001336

3 O'Connor RC, Wetherall K, Cleare S, *et al.* Mental health and well-being during the COVID-19 pandemic: longitudinal analyses of adults in the UK COVID-19 Mental Health & Wellbeing study. *Br J Psychiatry* 2020;:1–8. doi:10.1192/bjp.2020.212

4 Pierce M, Hope H, Ford T, *et al.* Mental health before and during the COVID-19 pandemic: a longitudinal probability sample survey of the UK population. *Lancet Psychiatry* 2020;**7**:883–92. doi:10.1016/S2215-0366(20)30308-4

5 WHO. Substantial investment needed to avert mental health crisis. https://www.who.int/news-room/detail/14-05-2020-substantial-investment-needed-to-avert-mental-health-crisis (accessed 1 Jun 2020).

6 Zalsman G, Stanley B, Szanto K, *et al.* Suicide in the Time of COVID-19: Review and Recommendations. *Arch Suicide Res* 2020;:1–6. doi:10.1080/13811118.2020.1830242

7 Psychiatrists see alarming rise in patients needing urgent and emergency care and forecast a 'tsunami' of mental illness. www.rcpsych.ac.uk. https://www.rcpsych.ac.uk/news-and-features/latest-news/detail/2020/05/15/psychiatrists-see-alarming-rise-in-patients-needing-urgent-and-emergency-care (accessed 1 Jun 2020).

8 Moutier C. Suicide Prevention in the COVID-19 Era: Transforming Threat Into Opportunity. *JAMA Psychiatry* Published Online First: 16 October 2020. doi:10.1001/jamapsychiatry.2020.3746

9 Stuckler D, Basu S, Suhrcke M, *et al.* The public health effect of economic crises and alternative policy responses in Europe: an empirical analysis. *The Lancet* 2009;**374**:315–23. doi:10.1016/S0140-6736(09)61124-7

10 Phillips JA, Nugent CN. Suicide and the Great Recession of 2007–2009: The role of economic factors in the 50 U.S. states. *Soc Sci Med* 2014;**116**:22–31. doi:10.1016/j.socscimed.2014.06.015

11 Reeves A, McKee M, Stuckler D. Economic suicides in the Great Recession in Europe and North America. *Br J Psychiatry* 2014;**205**:246–7. doi:10.1192/bjp.bp.114.144766

12 John A, Okolie C, Eyles E, *et al.* The impact of the COVID-19 pandemic on self-harm and suicidal behaviour: a living systematic review. *F1000Research* 2020;**9**:1097. doi:10.12688/f1000research.25522.1

13 John A, Pirkis J, Gunnell D, *et al.* Trends in suicide during the covid-19 pandemic. *BMJ* 2020;:m4352. doi:10.1136/bmj.m4352

For peer review only

14 Wasserman IM. The impact of epidemic, war, prohibition and media on suicide: United States, 1910-1920. *Suicide Life Threat Behav* 1992;**22**:240–54.

15 Chan SMS, Chiu FKH, Lam CWL, *et al.* Elderly suicide and the 2003 SARS epidemic in Hong Kong. *Int J Geriatr Psychiatry* 2006;**21**:113–8. doi:10.1002/gps.1432

16 Cheung YT, Chau PH, Yip PSF. A revisit on older adults suicides and Severe Acute Respiratory Syndrome (SARS) epidemic in Hong Kong. *Int J Geriatr Psychiatry* 2008;**23**:1231–8. doi:10.1002/gps.2056

17 *Statistics Sweden. Statistical yearbook of Sweden [Statistisk årsbok för Sverige 1914–2014]. https://www.scb.se/hitta-statistik/aldre-statistik/innehall/statistisk-arsbok-1914-2014/.*

18 Holmberg M. The ghost of pandemics past: revisiting two centuries of influenza in Sweden. *Med Humanit* 2017;**43**:141–7. doi:10.1136/medhum-2016-011023

19 The National Board of Health and Welfare. Cause of death statistics. History, production methods and reliability [Dödsorsaksstatistik. Historik, produktionsmetoder och tillförlitlighet]. www.socialstyrelsen.se. 2010.

20 Statistik om dödsorsaker. Socialstyrelsen. https://www.socialstyrelsen.se/statistik-och-data/statistik/statistikamnen/dodsorsaker/ (accessed 21 Nov 2020).

21 Shin Y, Yu B, Greenwood-Nimmo M. Modelling Asymmetric Cointegration and Dynamic Multipliers in a Nonlinear ARDL Framework. In: Sickles RC, Horrace WC, eds. *Festschrift in Honor of Peter Schmidt: Econometric Methods and Applications*. New York: : Springer Science+Business Media 2014. 281–314.

22 Norstrom T, Stickley A, Shibuya K. The importance of alcoholic beverage type for suicide in Japan: a time-series analysis, 1963-2007. *Drug Alcohol Rev* 2012;**31**:251–6. doi:10.1111/j.1465-3362.2011.00300.x

23 Gunnell D, Lopatatzidis A, Dorling D, *et al.* Suicide and unemployment in young people. Analysis of trends in England and Wales, 1921-1995. *Br J Psychiatry* 1999;**175**:263–70. doi:10.1192/bjp.175.3.263

24 Zortea TC, Brenna CTA, Joyce M, *et al.* The Impact of Infectious Disease-Related Public Health Emergencies on Suicide, Suicidal Behavior, and Suicidal Thoughts: A Systematic Review. *Crisis* 2020;:1–14. doi:10.1027/0227-5910/a000753

25 Dödligheten ökade första halvåret - covid-19 tredje vanligaste dödsorsaken - Socialstyrelsen. https://www.socialstyrelsen.se/om-socialstyrelsen/pressrum/press/dodligheten-okade-forsta-halvaret---covid-19-tredje-vanligaste-dodsorsaken/ (accessed 21 Nov 2020).

For peer review only

Figure legends

Figure 1. Annual suicide rate (scaled on the left arithmetic Y-axis) and influenza mortality (scaled on the right logarithmic Y-axis) in Sweden in 1910-1978 per 100 000 inhabitants. *Note*: To ease visualization, influenza death rates are reported on the logarithmic scale, while for the analysis, the logarithmic transformation was performed for suicide rates. Grey areas denote the time periods corresponding to the Spanish influenza pandemic ('S.flu', 1918-1920), the Asian influenza pandemic ('A.flu', 1957-1958), and the Hong Kong influenza pandemic ('HK.flu', 1968-1969) in Sweden.

Figure 2. Sex-specific annual suicide rate (scaled on the left arithmetic Y-axis) and influenza mortality (scaled on the right logarithmic Y-axis) in Sweden in 1910-1978 per 100 000 inhabitants of corresponding sex. *Note*: To ease visualization, influenza death rates are reported on the logarithmic scale, while for the analysis, the logarithmic transformation was performed for suicide rates. Grey areas denote the time periods corresponding to the Spanish influenza pandemic ('S.flu', 1918-1920), the Asian influenza pandemic ('A.flu', 1957-1958), and the Hong Kong influenza pandemic ('HK.flu', 1968-1969) in Sweden.

Figure 3. Age-standardized death rates due to suicide and influenza (scaled on the left Y-axis) in January-June 2000-2020 and age-standardized death rates due to Covid-19 (scaled on the right Y-axis) in January-June 2020 in Sweden among men and women per 100 000 inhabitants of corresponding sex. *Note*: Bars denote Covid-19 death rates in first half of 2020. All death rates in the figure are presented on arithmetic scales. The reported death rates are retrieved from the National Board of Health and Welfare tables published on November 17th, 2020.[25]

For peer review only - http://bmjopen.bmj.com/site/about/guidelines.xhtml

Table 1. Short-term and long-term relationship of suicide rates with positive and negative changes in influenza death rates among the whole population, men, and women in 1910-1978 in Sweden

	Whole population		Men		Women	
	**Coeff. (95% CI)**	**p-value**	**Coeff. (95% CI)**	**p-value**	**Coeff. (95% CI)**	**p-value**
**Short-term coefficients**						
Influenza +	0.00002 (-0.00036 to 0.00039)	0.931	0.00004 (-0.00034 to 0.00041)	0.854	-0.00007 (-0.00056 to 0.00041)	0.760
Influenza –	0.00103 (-0.00579 to 0.00785)	0.764	-0.00192 (-0.01005 to 0.00621)	0.638	0.00780 (0.00015 to 0.01544)	0.046
**Long-term coefficients**						
Influenza +	-0.00012	0.998	-0.01314	0.745	0.11254	0.538
Influenza –	0.00211	0.962	0.01443	0.722	-0.10789	0.544
**Model diagnostics**						
Q-test for autocorrelation, χ^2	40.160	0.125	35.260	0.273	31.850	0.424
Heteroscedasticity, χ^2	3.649	0.056	5.439	0.020	1.280	0.258
Normality, χ^2	2.826	0.243	2.237	0.327	2.372	0.305
RESET, F-statistics	4.656	0.006	5.473	0.023	1.013	0.394
CUSUM	Stabl., no str. break	NA	Stabl., no str. break	NA	Stabl., no str. break	NA
CUSUMQ	Stabl.	NA	Stabl.	NA	Stabl.	NA
Adj. R^2	0.257	NA	0.300	NA	0.374	NA
$Wald_{SR}$, F-statistics	0.054	0.817	0.275	0.602	1.062	0.307
$Wald_{LR}$, F-statistics	1.206	0.277	1.176	0.283	0.771	0.384

Note: Signs as "+" and "–" denote the exposure variable being partitioned in positive and negative changes, respectively. Wald test for asymmetry in the short-term ($Wald_{SR}$) and long-term ($Wald_{LR}$) has a null hypothesis of no cointegration. Q-test for autocorrelation reports the results of Portmanteau test for white noise. Heteroscedasticity is measured by Breusch-Pagan/Cook-Weisberg test. Normality is measured by Jarque-Bera test. RESET statistics refers to the regression specification error tests. CUSUM and CUSUMQ refer to the tests of the cumulative sum of recursive residuals and their squares, respectively. "Stabl., no str. break" in the results of CUSUM indicates that the model was found stable and the null hypothesis of no structural break was not rejected (same applies to "Stabl." as an output for CUSUMQ). "NA" denotes that a certain test parameter was not applicable.

For peer review only - http://bmjopen.bmj.com/site/about/guidelines.xhtml

Table 2. Short-term and long-term relationship of suicide rates with positive and negative changes in influenza death rates among the whole population, men, and women in 1918-1956 in Sweden

	Whole population		Men		Women	
	Coeff. (95% CI)	p-value	Coeff. (95% CI)	p-value	Coeff. (95% CI)	p-value
**Short-term coefficients**						
Influenza +	0.00002 (-0.00077 to 0.00082)	0.955	-0.00014 (-0.00102 to 0.00075)	0.755	0.00098 (-0.00068 to 0.00263)	0.237
Influenza –	0.00087 (-0.00643 to 0.00817)	0.809	-0.00132 (-0.01001 to 0.00741)	0.760	0.00434 (-0.00595 to 0.01463)	0.394
**Long-term coefficients**						
Influenza +	0.00011	0.994	-0.00490	0.802	0.01403	0.521
Influenza –	0.00103	0.936	0.00548	0.775	-0.01098	0.600
**Model diagnostics**						
Q-test for autocorrelation, χ^2	14.130	0.658	9.674	0.917	9.614	0.919
Heteroscedasticity, χ^2	0.939	0.333	0.768	0.381	5.042	0.025
Normality, χ^2	0.420	0.811	0.132	0.936	2.383	0.304
RESET, F-statistics	0.413	0.745	0.764	0.524	0.505	0.683
CUSUM	Stabl., no str. break	NA	Stabl., no str. break	NA	Stabl., no str. break	NA
CUSUMQ	Stabl.	NA	Stabl.	NA	Stabl.	NA
Adj. R^2	0.309	NA	0.345	NA	0.432	NA
$Wald_{SR}$, F-statistics	0.056	0.815	0.155	0.697	0.183	0.673
$Wald_{LR}$, F-statistics	1.555	0.222	0.217	0.645	4.479	0.044

Note: Signs as "+" and "–" denote the exposure variable being partitioned in positive and negative changes, respectively. Wald test for asymmetry in the short-term ($Wald_{SR}$) and long-term ($Wald_{LR}$) has a null hypothesis of no cointegration. Q-test for autocorrelation reports the results of Portmanteau test for white noise. Heteroscedasticity is measured by Breusch-Pagan/Cook-Weisberg test. Normality is measured by Jarque-Bera test. RESET statistics refers to the regression specification error tests. CUSUM and CUSUMQ refer to the tests of the cumulative sum of recursive residuals and their squares, respectively. "Stabl., no str. break" in the results of CUSUM indicates that the model was found stable and the null hypothesis of no structural break was not rejected (same applies to "Stabl." as an output for CUSUMQ). "NA" denotes that a certain test parameter was not applicable.

For peer review only - http://bmjopen.bmj.com/site/about/guidelines.xhtml

Table 3. Short-term and long-term relationship of suicide rates with positive and negative changes in influenza death rates among the whole population, men, and women in 1957-1978 in Sweden

	Whole population		Men		Women	
	**Coeff. (95% CI)**	**p-value**	**Coeff. (95% CI)**	**p-value**	**Coeff. (95% CI)**	**p-value**
**Short-term coefficients**						
Influenza +	-0.00964 (-0.02252 to 0.00324)	0.883	-0.00876 (-0.02738 to 0.00985)	0.328	-0.00976 (-0.02033 to 0.00081)	0.068
Influenza –	0.00194 (-0.02614 to 0.03003)	0.130	-0.01531 (-0.04658 to 0.01596)	0.309	0.02155 (-0.00343 to 0.04653)	0.085
**Long-term coefficients**						
Influenza +	0.01255	0.618	-0.02659	0.541	0.04091	0.064
Influenza –	-0.01033	0.684	0.024538	0.582	-0.02870	0.184
**Model diagnostics**						
Q-test for autocorrelation, χ^2	5.662	0.773	3.790	0.925	5.243	0.813
Heteroscedasticity, χ^2	0.102	0.750	0.155	0.694	0.079	0.778
Normality, χ^2	1.569	0.456	1.627	0.443	1.237	0.537
RESET, F-statistics	5.839	0.014	3.348	0.064	1.089	0.398
CUSUM	Stabl., no str. break	NA	Stabl., no str. break	NA	Stabl., no str. break	NA
CUSUMQ	Stabl.	NA	Stabl.	NA	Stabl.	NA
Adj. R^2	0.246	NA	0.337	NA	0.371	NA
$Wald_{SR}$, F-statistics	2.903	0.112	0.536	0.477	7.132	0.019
$Wald_{LR}$, F-statistics	0.922	0.354	0.297	0.595	26.74	<0.001

Note: Signs as "+" and "–" denote the exposure variable being partitioned in positive and negative changes, respectively. Wald test for asymmetry in the short-term ($Wald_{SR}$) and long-term ($Wald_{LR}$) has a null hypothesis of no cointegration. Q-test for autocorrelation reports the results of Portmanteau test for white noise. Heteroscedasticity is measured by Breusch-Pagan/Cook-Weisberg test. Normality is measured by Jarque-Bera test. RESET statistics refers to the regression specification error tests. CUSUM and CUSUMQ refer to the tests of the cumulative sum of recursive residuals and their squares, respectively. "Stabl., no str. break" in the results of CUSUM indicates that the model was found stable and the null hypothesis of no structural break was not rejected (same applies to "Stabl." as an output for CUSUMQ). "NA" denotes that a certain test parameter was not applicable.

For peer review only - http://bmjopen.bmj.com/site/about/guidelines.xhtml

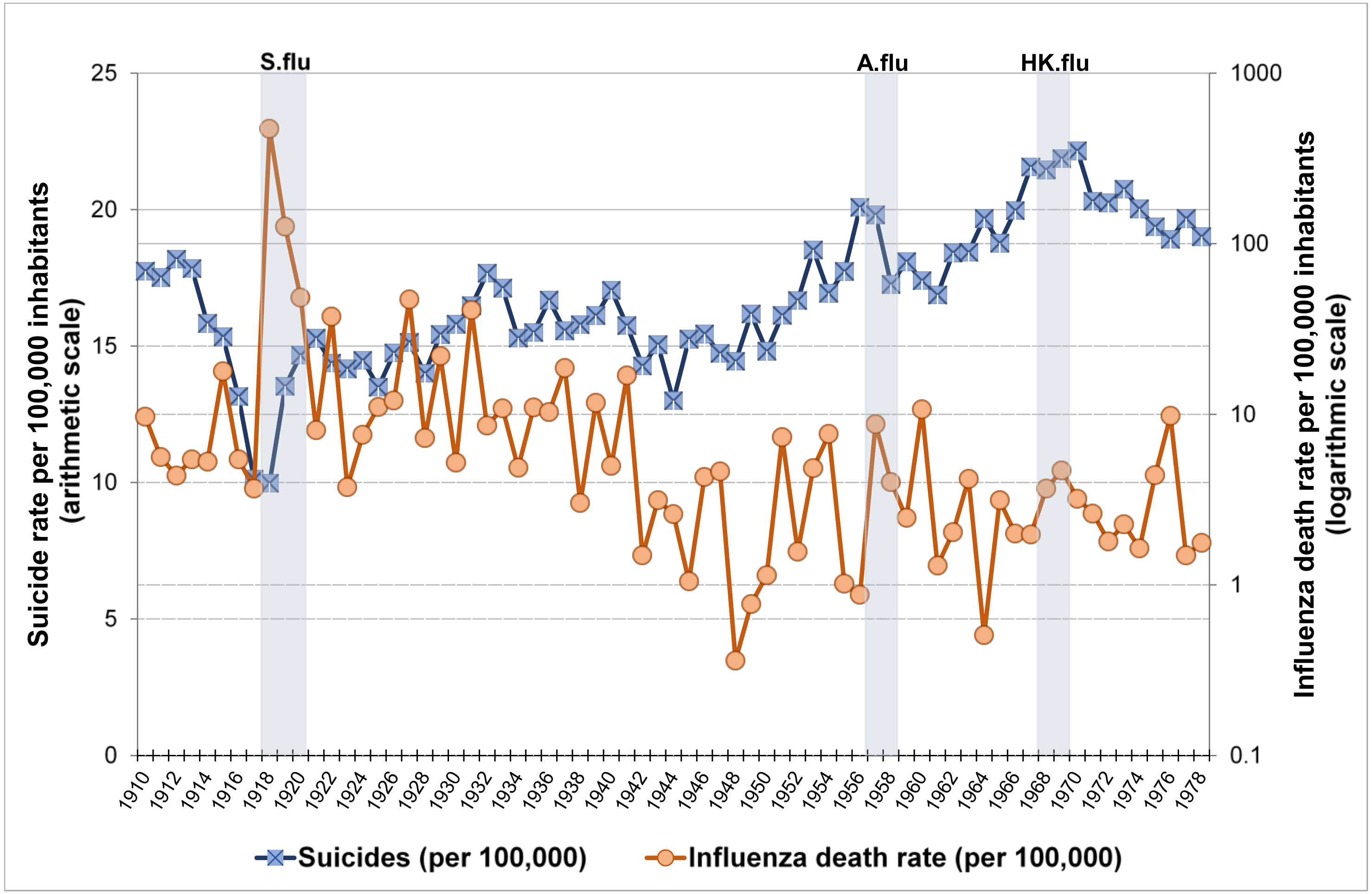

Figure 1. Annual suicide rate (scaled on the left arithmetic Y-axis) and influenza mortality (scaled on the right logarithmic Y-axis) in Sweden in 1910-1978 per 100 000 inhabitants. *Note*: To ease visualization, influenza death rates are reported on the logarithmic scale, while for the analysis, the logarithmic transformation was performed for suicide rates. Grey areas denote the time periods corresponding to the Spanish influenza pandemic ('S.flu', 1918-1920), the Asian influenza pandemic ('A.flu', 1957-1958), and the Hong Kong influenza pandemic ('HK.flu', 1968-1969) in Sweden.

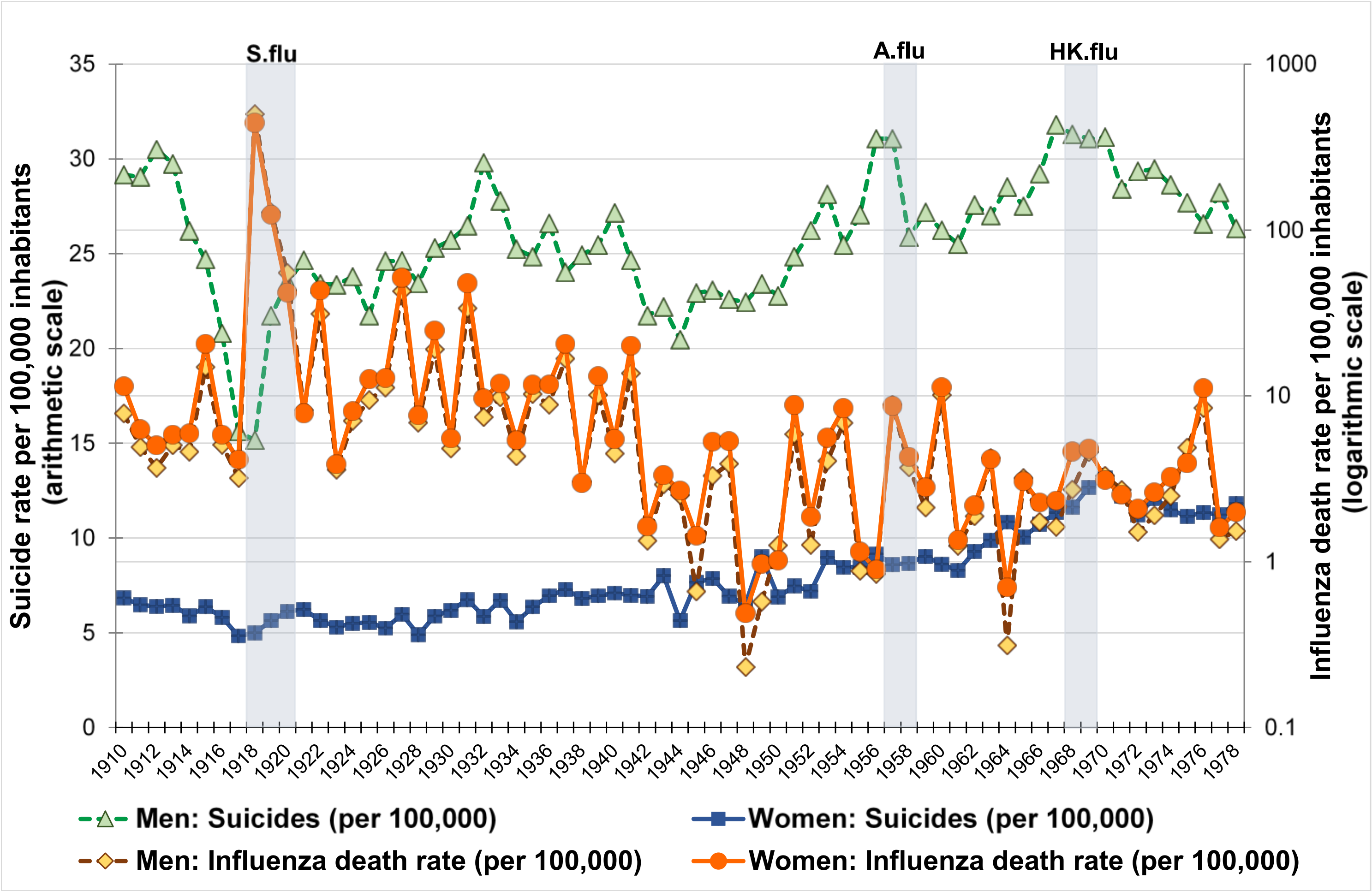

Figure 2. Sex-specific annual suicide rate (scaled on the left arithmetic Y-axis) and influenza mortality (scaled on the right logarithmic Y-axis) in Sweden in 1910-1978 per 100,000 inhabitants of corresponding sex. *Note*: To ease visualization, influenza death rates are reported on the logarithmic scale, while for the analysis, the logarithmic transformation was performed for suicide rates. Grey areas denote the time periods corresponding to the Spanish influenza pandemic ('S.flu', 1918-1920), the Asian influenza pandemic ('A.flu', 1957-1958), and the Hong Kong influenza pandemic ('HK.flu', 1968-1969) in Sweden.

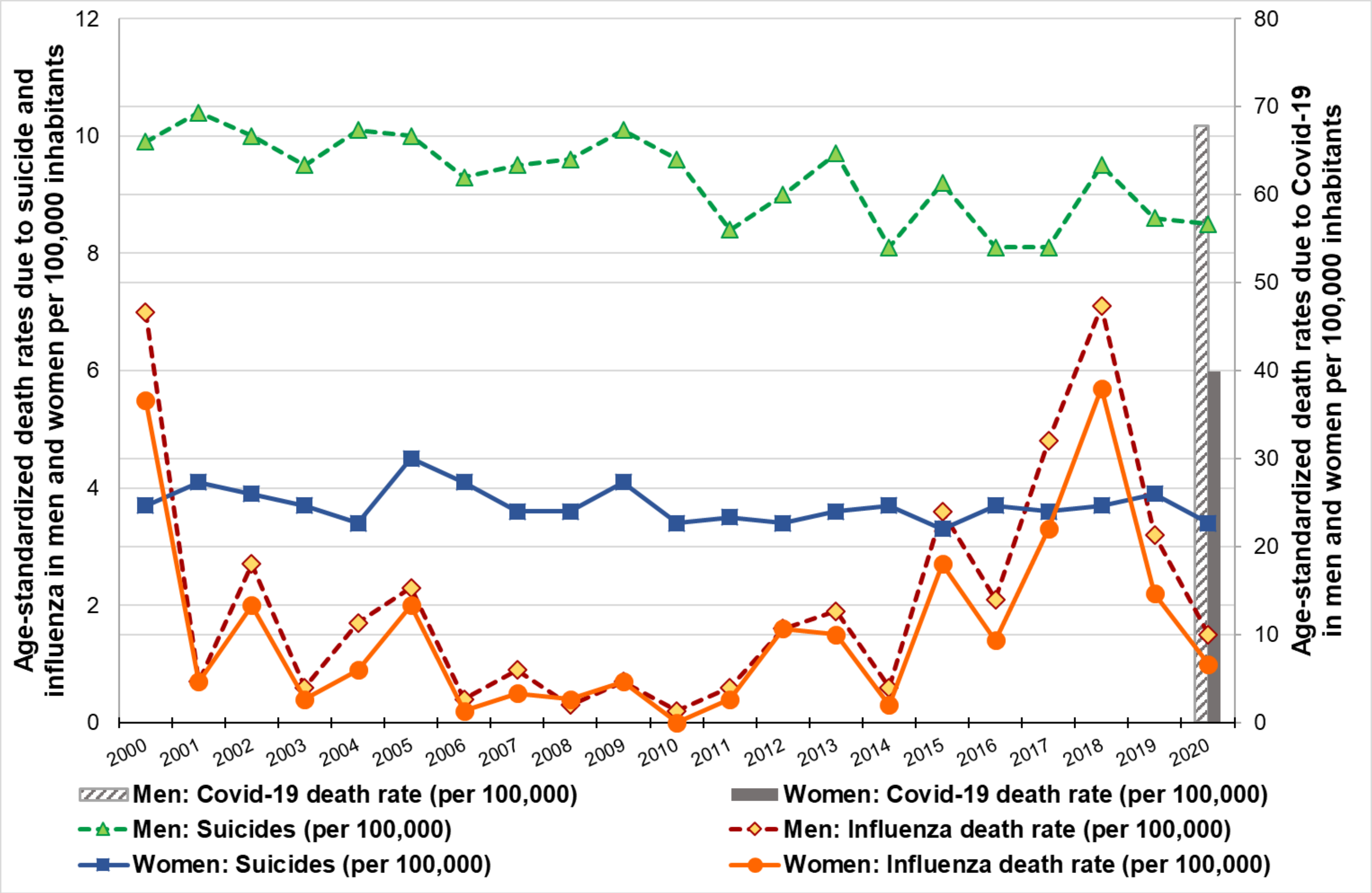

Figure 3. Age-standardized death rates due to suicide and influenza (scaled on the left Y-axis) in January-June 2000-2020 and age-standardized death rates due to Covid-19 (scaled on the right Y-axis) in January-June 2020 in Sweden among men and women per 100 000 inhabitants of corresponding sex. *Note*: Bars denote Covid-19 death rates in first half of 2020. All death rates in the figure are presented on arithmetic scales. The reported death rates are retrieved from the National Board of Health and Welfare tables published on November 17th, 2020.[25]

For peer review only - http://bmjopen.bmj.com/site/about/guidelines.xhtml

Supplementary material

eTable 1. Original data on the number of deaths by influenza and suicide, and population size for total population, men, and women in 1910-1978, retrieved from the Statistical Yearbook of Sweden from 1910-1978

Year	Total population, N	Men, N	Women, N	Deaths by influenza in total population, N	Deaths by influenza among men, N	Deaths by influenza among women, N	Deaths by suicide in total population, N	Deaths by suicide among men, N	Deaths by suicide among women, N
1910	5522403	2698729	2823674	534	212	322	980	787	193
1911	5561799	2718638	2843161	312	134	178	974	790	184
1912	5604192	2740737	2863455	245	101	144	1019	836	183
1913	5638583	2756946	2881637	307	139	168	1006	820	186
1914	5679607	2777447	2902160	300	128	172	899	728	171
1915	5712740	2794552	2918188	1018	417	601	876	690	186
1916	5757566	2817950	2939616	314	143	171	757	586	171
1917	5800847	2841554	2959293	213	91	122	587	444	143
1918	5813850	2849205	2964645	27379	14245	13134	580	432	148
1919	5847037	2868395	2978642	7341	3667	3674	791	623	168
1920	5904489	2898256	3006233	2853	1596	1257	866	682	184
1921	5954316	2925988	3028328	479	241	238	910	721	189
1922	5987520	2944031	3043489	2235	918	1317	861	689	172
1923	6005759	2948508	3057251	224	106	118	851	689	162
1924	6036118	2964230	3071888	458	210	248	874	705	169
1925	6053562	2972554	3081008	669	280	389	817	646	171
1926	6074368	2982625	3091743	731	335	396	896	734	162
1927	6087923	2990205	3097718	2875	1277	1598	921	736	185
1928	6105190	2999562	3105628	444	207	237	855	703	152
1929	6120080	3007946	3112134	1345	573	772	944	761	183
1930	6142191	3020848	3121343	319	146	173	970	777	193
1931	6162446	3037064	3125382	2518	1025	1493	1015	804	211
1932	6190364	3053528	3136836	531	228	303	1094	910	184
1933	6211566	3066888	3144678	673	300	373	1063	852	211
1934	6233090	3079690	3153400	303	133	170	953	777	176
1935	6250506	3090451	3160055	685	316	369	969	768	201
1936	6266888	3100534	3166354	646	275	371	1045	825	220
1937	6284722	3111256	3173466	1173	522	651	978	747	231
1938	6310214	3125000	3185214	190	95	95	996	779	217
1939	6341303	3142356	3198947	740	319	421	1022	800	222
1940	6371432	3160128	3211304	318	142	176	1086	858	228
1941	6406474	3180535	3225939	1082	434	648	1009	784	225
1942	6458200	3207756	3250444	96	43	53	922	697	225
1943	6522827	3240631	3282196	204	95	109	982	719	263
1944	6597348	3279723	3317625	172	83	89	858	671	187
1945	6673749	3321502	3352247	70	22	48	1018	761	257
1946	6763685	3366694	3396991	291	111	180	1044	777	267
1947	6842046	3407577	3434469	316	133	183	1008	770	238

For peer review only - http://bmjopen.bmj.com/site/about/guidelines.xhtml

Supplementary material

1948	6924888	3448122	3476766	25	8	17	1000	774	226
1949	6986181	3479079	3507102	54	20	34	1130	814	316
1950	7041829	3506442	3535387	80	44	36	1043	799	244
1951	7098740	3535736	3563004	522	208	314	1145	879	266
1952	7150606	3562475	3588131	112	45	67	1192	934	258
1953	7192316	3583598	3608718	347	145	202	1332	1008	324
1954	7234664	3605013	3629651	553	248	305	1225	918	307
1955	7290112	3633983	3656129	74	32	42	1293	983	310
1956	7338991	3659917	3679074	64	31	33	1474	1137	337
1957	7388611	3685654	3702957	649	327	322	1463	1145	318
1958	7429675	3706039	3723636	297	137	160	1282	959	323
1959	7462823	3722867	3739956	184	79	105	1350	1012	338
1960	7497967	3740119	3757848	801	378	423	1305	981	324
1961	7542028	3763040	3778988	98	47	51	1273	960	313
1962	7581148	3782252	3798896	154	71	83	1396	1043	353
1963	7627507	3805699	3821808	319	160	159	1406	1028	378
1964	7695200	3840897	3854303	39	12	27	1514	1096	418
1965	7772506	3882473	3890033	243	124	119	1459	1068	391
1966	7843088	3919170	3923918	157	68	89	1566	1145	421
1967	7892774	3942223	3950551	156	64	92	1702	1254	448
1968	7934996	3961414	3973582	291	108	183	1702	1240	462

For peer review only

Supplementary material

Supplementary material. Model diagnostics

We carried out several types of model diagnostics, including Portmanteau test for white noise to check autocorrelation in residuals, Breusch-Pagan/Cook-Weisberg test for heteroscedasticity, Jarque-Bera test on normality, and the regression specification error tests that indicates whether there is a misspecification in the model. We also performed tests for stability of the models by using the cumulative sum of recursive residuals and their squares to test whether there is a structural break due to changes in regression coefficients over time.

Supplementary material. STATA codes for the non-linear autoregressive distributed lag (NARDL) modelling of association between between influenza death rates and suicide rates

```
clear
use "Z:\Influenza_deaths_suicides\ALL19101978.dta"


///// 1. DATA MANAGEMENT: generate and label variables to be used in the analyses


/// Labels for original variables (retrieved from the Statistical Yearbooks 1910-1978)

 lab var year "Years 1910-1978"

 lab var number_population_all "Total number of inhabitants (population size) in Sweden in corresponding year"
 lab var number_population_men "Total number of male population in Sweden in corresponding year"
 lab var number_population_women "Total number of female population in Sweden in corresponding year"

 lab var number_influenza_all "Number of deaths from influenza in total population in corresponding year"
 lab var number_influenza_men "Number of deaths from influenza among men in corresponding year"
 lab var number_influenza_women "Number of deaths from influenza among women in corresponding year"

 lab var number_suicide_all "Number of suicides in total population in corresponding year"
 lab var number_suicide_men "Number of suicides among men in corresponding year"
 lab var number_suicide_women "Number of suicides among women in corresponding year"



/// Calculation of annual influenza death rates and suicide rates per 100,000 for total population, men, and
women

 gen influenza_rates_all = (number_influenza_all/number_population_all)*100000
 gen influenza_rates_men = (number_influenza_men/number_population_men)*100000
 gen influenza_rates_women = (number_influenza_women/number_population_women)*100000

 gen suicide_rates_all = (number_suicide_all/number_population_all)*100000
 gen suicide_rates_men = (number_suicide_men/number_population_men)*100000
 gen suicide_rates_women = (number_suicide_women/number_population_women)*100000

 lab var influenza_rates_all "Influenza death rates for total population per 100,000, annual"
 lab var influenza_rates_men "Influenza death rates among men per 100,000, annual"
 lab var influenza_rates_women "Influenza death rates among women per 100,000, annual"

 lab var suicide_rates_all "Suicide rates for total population per 100,000, annual"
 lab var suicide_rates_men "Suicide rates among men per 100,000, annual"
 lab var suicide_rates_women "Suicide rates among women per 100,000, annual"



/// Logarithmic transformation for suicide rates for total population, men, and women
```


For peer review only - http://bmjopen.bmj.com/site/about/guidelines.xhtml

Supplementary material

```
gen ln_suicide_rates_all = ln(suicide_rates_all)
gen ln_suicide_rates_men = ln(suicide_rates_men)
gen ln_suicide_rates_women = ln(suicide_rates_women)

lab var ln_suicide_rates_all "Log-transformed suicide rates for total population per 100,000, annual"
lab var ln_suicide_rates_men "Log-transformed suicide rates among men per 100,000, annual"
lab var ln_suicide_rates_women "Log-transformed suicide rates among women per 100,000, annual"



/// Variables for changes in death registration in Sweden 1910-1978 (as dummy variables)

// based on the Bertillon criteria (prior to 1931)
gen registration_19101930=0
replace registration_19101930=1 if year<=1930
lab var registration_19101930 "Dummy variable for death registration in 1910-1930 (Bertillion)"

// introduced in cooperation with other Nordic countries (1931-1950)
gen registration_19311950=0
replace registration_19311950=1 if year>=1931 & year<=1950
lab var registration_19311950 "Dummy variable for death registration in 1931-1950 (new registration)"

// ICD-6 (1951-1957)
gen registration_19511957=0
replace registration_19511957=1 if year>=1951 & year<=1957
lab var registration_19511957 "Dummy variable for death registration in 1951-1957 (ICD-6)"

// ICD-7 (1958-1968)
gen registration_19581968=0
replace registration_19581968=1 if year>=1958 & year<=1968
lab var registration_19581968 "Dummy variable for death registration in 1958-1968 (ICD-7)"

// ICD-8 (1969-1978)
gen registration_1969after=0
replace registration_1969after=1 if year>=1969
lab var registration_1969after "Dummy variable for death registration in 1969 and after (ICD-8)"
//////////////////////////////////////////////////////////////////////////////



///// 2. CHECKING VARIABLES' PROPERTIES AND TESTING THE CONDITIONS FOR MODELLING


/// Declare data to be time-series data
tsset year, yearly


/// Obtain optimal lags for each variable (for (i) augmentation in ADF and KPSS tests, and (ii) for p and q
parameters in NARDL).
/// Lags obtained for influenza death rates and logarithmically-transformed suicide rates for total population,
men, and women, and for time periods 1910-1978, 1918-1956, and 1957-1978
/// If AIC, HQIC, and SBIC information criteria indicated different lag orders, SBIC was used to select optimal
lags

varsoc influenza_rates_all
varsoc influenza_rates_all if tin(1918, 1956)
varsoc influenza_rates_all if tin(1957, )

varsoc influenza_rates_men
```


For peer review only

Supplementary material

```
varsoc influenza_rates_men if tin(1918, 1956)
varsoc influenza_rates_men if tin(1957, )

varsoc influenza_rates_women
varsoc influenza_rates_women if tin(1918, 1956)
varsoc influenza_rates_women if tin(1957, )

varsoc ln_suicide_rates_all
varsoc ln_suicide_rates_all if tin(1918, 1956)
varsoc ln_suicide_rates_all if tin(1957, )

varsoc ln_suicide_rates_men
varsoc ln_suicide_rates_men if tin(1918, 1956)
varsoc ln_suicide_rates_men if tin(1957, )

varsoc ln_suicide_rates_women
varsoc ln_suicide_rates_women if tin(1918, 1956)
varsoc ln_suicide_rates_women if tin(1957, )


/// Tests for stationarity: ADF and KPSS for influenza death rates and logarithmically-transformed suicide rates variables for total population, men, and women
/// Augmentation by at least one lag was used (for suicide rates in women - by two lags according to SBIC in varsoc)

dfuller influenza_rates_all, lag(1)
dfuller influenza_rates_men, lag(1)
dfuller influenza_rates_women, lag(1)

dfuller ln_suicide_rates_all, lag(1)
dfuller ln_suicide_rates_men, lag(1)
dfuller ln_suicide_rates_women, lag(1)
dfuller ln_suicide_rates_women, lag(2)

dfuller D.influenza_rates_all, lag(1)
dfuller D.influenza_rates_men, lag(1)
dfuller D.influenza_rates_women, lag(1)

dfuller D.ln_suicide_rates_all, lag(1)
dfuller D.ln_suicide_rates_men, lag(1)
dfuller D.ln_suicide_rates_women, lag(1)
dfuller D.ln_suicide_rates_women, lag(2)

kpss influenza_rates_all, maxlag(1) notrend
kpss influenza_rates_men, maxlag(1) notrend
kpss influenza_rates_women, maxlag(1) notrend

kpss ln_suicide_rates_all, maxlag(1) notrend
kpss ln_suicide_rates_men, maxlag(1) notrend
kpss ln_suicide_rates_women, maxlag(2) notrend

kpss D.influenza_rates_all, maxlag(1) notrend
kpss D.influenza_rates_men, maxlag(1) notrend
kpss D.influenza_rates_women, maxlag(1) notrend

kpss D.ln_suicide_rates_all, maxlag(1) notrend
kpss D.ln_suicide_rates_men, maxlag(1) notrend
kpss D.ln_suicide_rates_women, maxlag(2) notrend
```


For peer review only
For peer review only - http://bmjopen.bmj.com/site/about/guidelines.xhtml

Supplementary material

///// 3. ESTIMATION OF NON-LINEAR AUTOREGRESSIVE DISTRIBUTED LAG (NARDL) MODELS: for total population, men, and women, and for time periods 1910-1978, 1918-1956, and 1957-1978
/// The dependent and independent variables are indicated in levels.
/// The covariates (i.e., changes in death registration system 1910-1978) are included with the deterministic option, but these were only kept in the models if statistically significant.
/// The optimal number of lags for dependent and independent variables (p and q parameters, respectively).
/// Since p and q parameters refer to levels, one additional lag is added to p and q to get an optimal lag length in differences (p and q must be at least 2).
/// The model provides an output for a long-term cointegration bounds test and diagnostic tests.

nardl ln_suicide_rates_all influenza_rates_all, p(2) q(2) h(69) plot bootstrap(100) level(95) residuals /* used as a final model */
nardl ln_suicide_rates_all influenza_rates_all, p(2) q(2) deterministic(registration_19101930 registration_19311950 registration_19511957 registration_19581968 registration_1969after) h(69) plot bootstrap(100) level(95) residuals

nardl ln_suicide_rates_men influenza_rates_men, p(2) q(2) h(69) plot bootstrap(100) level(95) residuals /* used as a final model */
nardl ln_suicide_rates_men influenza_rates_men, p(2) q(2) deterministic(registration_19101930 registration_19311950 registration_19511957 registration_19581968 registration_1969after) h(69) plot bootstrap(100) level(95) residuals

nardl ln_suicide_rates_women influenza_rates_women, p(3) q(2) h(69) plot bootstrap(100) level(95) residuals /* used as a final model */
nardl ln_suicide_rates_women influenza_rates_women, p(3) q(2) deterministic(registration_19101930 registration_19311950 registration_19511957 registration_19581968 registration_1969after) h(69) plot bootstrap(100) level(95) residuals

nardl ln_suicide_rates_all influenza_rates_all if tin(1918,1956), p(2) q(2) h(39) plot bootstrap(50) level(95) residuals
nardl ln_suicide_rates_men influenza_rates_men if tin(1918,1956), p(2) q(2) h(39) plot bootstrap(50) level(95) residuals
nardl ln_suicide_rates_women influenza_rates_women if tin(1918,1956), p(5) q(2) h(39) plot bootstrap(50) level(95) residuals

nardl ln_suicide_rates_all influenza_rates_all if tin(1957,), p(2) q(2) h(22) plot bootstrap(50) level(95) residuals
nardl ln_suicide_rates_men influenza_rates_men if tin(1957,), p(2) q(2) h(22) plot bootstrap(50) level(95) residuals
nardl ln_suicide_rates_women influenza_rates_women if tin(1957,), p(2) q(2) h(22) plot bootstrap(50) level(95) residuals

/* REFERENCES
For varsoc and dfuller:
StataCorp. Stata 15 Base Reference Manual. College Station, TX: Stata Press; 2017

For kpss:
Author: Christopher F Baum, Boston College, USA baum@@bc.edu
Kwiatkowski, D., Phillips, P.C.B., Schmidt, P. and Y. Shin. Testing the null hypothesis of stationarity against the alternative of a unit root: How sure are we that economic time series have a unit root? Journal of Econometrics, 54, 1992, 159-178.
Lee, D. and P. Schmidt. On the power of the KPSS test of stationarity against fractionally-integrated alternatives. Journal of Econometrics, 73, 1996, 285-302.
Schwert, G.W. Tests for Unit Roots: A Monte Carlo Investigation. Journal of Business and Economic Statistics, 7, 1989, 147-160.

For nardl:

For peer review only - http://bmjopen.bmj.com/site/about/guidelines.xhtml

Supplementary material

Author: Stata implementation by Marco Sunder (sunder@wifa.uni-leipzig.de). This version was created on 26jan2012.
Shin, Y., Yu, B., Greenwood-Nimmo, M. (2011): Modelling asymmetric cointegration and dynamic multipliers in a nonlinear ARDL framework. Working paper (version of November 2011), http://ssrn.com/abstract=1807745. */

end of do-file

For peer review only

Supplementary material

eTable 2. Unit root tests at the level and first difference by Augmented Dickey-Fuller (ADF) and Kwiatkowski-Phillips-Schmidt-Shin (KPSS) test statistics

	**ADF at level**		**KPSS at level**		**Results for stationarity at the level**	**ADF at first difference**		**KPSS at first difference**		**Results for stationarity at the 1st difference**	**Order of integration**
	**Test statistic**	**p-value**	**Test statistic**	**p-value[1]**		**Test statistic**	**p-value**	**Test statistic**	**p-value[1]**		
**Suicide rate (log-transformed)**											
All	-2.181	0.213	1.980	<0.001	nonstationary	-6.640	<0.001	0.068	>0.1	stationary	1
Males	-3.230	0.018	0.964	<0.001	nonstationary	-6.512	<0.001	0.053	>0.1	stationary	1
Females	-0.517	0.889	3.130	<0.001	nonstationary	-8.692	<0.001	0.104	>0.1	stationary	1
**Influenza death rate**											
All	-4.895	<0.001	0.461	>0.1	stationary	*-8.727*	*<0.001*	*0.016*	>0.1	*stationary*	0
Males	-4.856	<0.001	0.431	>0.1	stationary	*-8.642*	*<0.001*	*0.016*	>0.1	*stationary*	0
Females	-4.940	<0.001	0.491	>0.1	stationary	*-8.824*	*<0.001*	*0.015*	>0.1	*stationary*	0

Note: For the ADF test, the null hypothesis implies that the variable contains a unit root (the alternate hypothesis is that the variable is stationary), whereas for the KPSS test the null hypothesis implies that the variable is stationary (the alternate hypothesis is that there is a unit root). The results for ADF and KPSS tests for stationarity at first difference for influenza death rates are reported as explanatory (written in Italics) since the stationarity at the level has already been established (i.e., integrated of the order zero).

[1] KPSS test results do not indicate the exact p-value, but report the level of significance at which the null hypothesis is rejected (1%, 2.5%, 5%, or 10% significance level).

Abbreviations: ADF, Augmented Dickey–Fuller unit-root test; KPSS, Kwiatkowski-Phillips-Schmidt-Shin test for stationarity

For peer review only

Supplementary material

eTable 3. Full specification for non-linear autoregressive distributed lag models used for the analysis of short-term and long-term relationship of suicide rates with positive and negative changes in influenza death rates among the whole population, men, and women in 1910-1978 in Sweden

	Whole population		Men		Women	
	**Coefficient (95% CI)**	**p-value**	**Coefficient (95% CI)**	**p-value**	**Coefficient (95% CI)**	**p-value**
LnSuicide, lag 1	-0.08466 (-0.23940 to 0.07009)	0.278	-0.11457 (-0.30588 to 0.07674)	0.235	-0.04517 (-0.15832 to 0.06799)	0.427
Influenza +, lag 1	-0.00001 (-0.00761 to 0.00759)	0.998	-0.00150 (-0.01032 to 0.00732)	0.734	0.00508 (-0.00366 to 0.01382)	0.249
Influenza –, lag 1	-0.00018 (-0.00775 to 0.00739)	0.962	-0.00165 (-0.01044 to 0.00714)	0.708	0.00487 (-0.00383 to 0.01358)	0.267
ΔLnSuicide, lag 1	-0.14610 (-0.43113 to 0.13893)	0.309	0.029465 (-0.27204 to 0.33097)	0.846	-0.65184 (-0.91785 to -0.38582)	<0.001
ΔLnSuicide, lag 2	NA	NA	NA	NA	-0.30807 (-0.55722 to -0.05892)	0.016
ΔInfluenza +	0.00002 (-0.00036 to 0.00039)	0.931	0.00004 (-0.00034 to 0.00041)	0.854	-0.00007 (-0.00056 to 0.00041)	0.760
ΔInfluenza +, lag 1	0.00153 (-0.00285 to 0.00590)	0.488	0.00085 (-0.00347 to 0.00518)	0.694	0.00093 (-0.00482 to 0.00668)	0.747
ΔInfluenza –	0.00103 (-0.00579 to 0.00785)	0.764	-0.00192 (-0.01005 to 0.00621)	0.638	0.00780 (0.00015 to 0.01544)	0.046
ΔInfluenza –, lag 1	-0.00070 (-0.00236 to 0.00096)	0.401	-0.00038 (-0.00212 to 0.00136)	0.663	-0.000075 (-0.00289 to 0.00139)	0.488
**Long-term effect**						
Influenza +	-0.00012	0.998	-0.01314	0.745	0.11254	0.538
Influenza –	0.00211	0.962	0.01443	0.722	-0.10789	0.544
**Model diagnostics**						

For peer review only - http://bmjopen.bmj.com/site/about/guidelines.xhtml

Supplementary material

Q-test for autocorrelation, χ^2	40.160	0.125	35.260	0.273	31.850	0.424
Heteroscedasticity, χ^2	3.649	0.056	5.439	0.020	1.280	0.258
Normality, χ^2	2.826	0.243	2.237	0.327	2.372	0.305
RESET, F-statistics	4.656	0.006	5.473	0.023	1.013	0.394
CUSUM	Stable, no structural break	NA	Stable, no structural break	NA	Stable, no structural break	NA
CUSUMQ	Stable	NA	Stable	NA	Stable	NA
Adj. R^2	0.257	NA	0.300	NA	0.374	NA
$Wald_{SR}$, F-statistics	0.054	0.817	0.275	0.602	1.062	0.307
$Wald_{LR}$, F-statistics	1.206	0.277	1.176	0.283	0.771	0.384
**Cointegration test statistics**						
t_BDM	-1.095	NA	-1.199	NA	-0.799	NA
F_PSS	3.789	NA	3.735	NA	2.530	NA
**Critical values for F_PSS**						
5% critical values; I(0), I(1)	k=1: 5.055, 5.915<br>k=2: 3.947, 5.020	NA	k=1: 5.055, 5.915<br>k=2: 3.947, 5.020	NA	k=1: 5.055, 5.915<br>k=2: 3.947, 5.020	NA

Note: F_PSS denotes F-statistics for Pesaran/Shin/Smith bounds test with null hypothesis of no cointegration. Critical values are retrieved from Narayan PK (2005) for a sample size of n=70.[1] The null hypothesis of no cointegration is supported if F_PSS statistics is lower than 5% I(0) critical value; the null hypothesis is rejected if F_PSS statistics is greater than 5% I(1) critical value. We applied a conservative approach to considering the number of exposure variables (k), as suggested by Shin Y, et al (2014) [2] (since influenza death rates represent one exposure (k=1), while the analysis actually partitions the

For peer review only - http://bmjopen.bmj.com/site/about/guidelines.xhtml

exposure to positive and negative changes (k=2)). In a given table, the results of F_PSS for the whole population, men, and women are lower than the reported 5% I(0) critical values that accepts a null hypothesis of no cointegration.

"LnSuicide" indicates that suicide rates were logarithmically-transformed. "Δ" signifies the series is differenced. Signs as "+" and "–" denote the exposure variable being partitioned in positive and negative changes, respectively. "NA" denotes that a certain test or test parameter is not applicable.

Number of lags used for each variable in the model are noted by "lag#". To select the optimal number of lags to be used for choosing p and q parameters for the NARDL (i.e., numbers of lags for dependent and independent variables, respectively), we applied a varsoc command in STATA using the minimal values of Akaike Information Criterion, Schwarz's Bayesian information criterion (SBIC), and the Hannan and Quinn information criterion information criteria. If information criteria indicated different lag orders, SBIC was used to select optimal lags. Thus, for the analysis of the whole population and men, both exposure and outcome time series were lagged once, while for analysis among women to outcome time series were lagged twice and exposure time series were lagged once.

Wald test for asymmetry in a short-term ($Wald_{SR}$) and long-term ($Wald_{LR}$) has a null hypothesis of no cointegration. Q-test for autocorrelation reports the results of Portmanteau test for white noise. Heteroscedasticity is measured by Breusch-Pagan/Cook-Weisberg test. Normality is measured by Jarque-Bera test. RESET statistics refers to the regression specification error tests. CUSUM and CUSUMQ refer to the tests of the cumulative sum of recursive residuals and their squares, respectively. "Stable, no structural break" in the results of CUSUM indicates that the model was found stable and the null hypothesis of no structural break was not rejected (same applies to "Stable" as an output for CUSUMQ).

For peer review only

Supplementary material

eTable 4. Full specification for non-linear autoregressive distributed lag models used for the analysis of short-term and long-term relationship of suicide rates with positive and negative changes in influenza death rates among the whole population, men, and women in 1918-1956 in Sweden

	Whole population		Men		Women	
	Coefficient (95% CI)	p-value	Coefficient (95% CI)	p-value	Coefficient (95% CI)	p-value
LnSuicide, lag 1	-0.32229 (-0.73472 to 0.09015)	0.121	-0.26417 (-0.60333 to 0.07492)	0.122	-0.36067 (-0.97384 to 0.25250)	0.238
Influenza +, lag 1	0.00003 (-0.00851 to 0.00858)	0.994	-0.00129 (-0.011384 to 0.00879)	0.795	0.00506 (-0.00682 to 0.01695)	0.390
Influenza –, lag 1	-0.00033 (-0.00869 to 0.00803)	0.936	-0.00145 (-0.01129 to 0.00840)	0.766	0.00396 (-0.00801 to 0.01593)	0.503
ΔLnSuicide, lag 1	-0.19387 (-0.59955 to 0.21181)	0.337	-0.03045 (-0.44243 to 0.38153)	0.881	-0.58189 (-1.19784 to 0.03406)	0.063
ΔLnSuicide, lag 2	NA	NA	NA	NA	-0.36148 (-0.91643 to 0.19346)	0.193
ΔLnSuicide, lag 3	NA	NA	NA	NA	-0.11520 (-0.61658 to 0.38617)	0.641
ΔLnSuicide, lag 4	NA	NA	NA	NA	0.11306 (-0.26409 to 0.49021)	0.544
ΔInfluenza +	0.00002 (-0.00077 to 0.00082)	0.955	-0.00014 (-0.00102 to 0.00075)	0.755	0.00098 (-0.00068 to 0.00263)	0.237
ΔInfluenza +, lag 1	0.00140 (-0.00347 to 0.00627)	0.561	0.00095 (-0.00389 to 0.00580)	0.691	-0.00063 (-0.00821 to 0.00696)	0.866
ΔInfluenza –	0.00087 (-0.00643 to 0.00817)	0.809	-0.00132 (-0.01001 to 0.00741)	0.760	0.00434 (-0.00595 to 0.01463)	0.394
ΔInfluenza –, lag 1	-0.00076 (-0.00256 to 0.00104)	0.396	-0.00044 (-0.00233 to 0.00145)	0.637	-0.00052 (-0.00328 to 0.00224)	0.704
**Long-term effect**						
Influenza +	0.00011	0.994	-0.00490	0.802	0.01403	0.521

For peer review only - http://bmjopen.bmj.com/site/about/guidelines.xhtml

Supplementary material

Influenza –	0.00103	0.936	0.00548	0.775	-0.01098	0.600
**Model diagnostics**						
Q-test for autocorrelation, χ^2	14.130	0.658	9.674	0.917	9.614	0.919
Heteroscedasticity, χ^2	0.939	0.333	0.768	0.381	5.042	0.025
Normality, χ^2	0.420	0.811	0.132	0.936	2.383	0.304
RESET, F-statistics	0.413	0.745	0.764	0.524	0.505	0.683
CUSUM	Stable, no structural break	NA	Stable, no structural break	NA	Stable, no structural break	NA
CUSUMQ	Stable	NA	Stable	NA	Stable	NA
Adj. R^2	0.309	NA	0.345	NA	0.432	NA
$Wald_{SR}$, F-statistics	0.056	0.815	0.155	0.697	0.183	0.673
$Wald_{LR}$, F-statistics	1.555	0.222	0.217	0.645	4.479	0.044
**Cointegration test statistics**						
t_BDM	-1.596	NA	-1.591	NA	-1.207	NA
F_PSS	1.009	NA	1.019	NA	1.328	NA
**Critical values for F_PSS**						
5% critical values; I(0) to I(1)	k=1: 5.260, 6.160<br>k=2: 4.133, 5.260	NA	k=1: 5.260, 6.160<br>k=2: 4.133, 5.260	NA	k=1: 5.260, 6.160<br>k=2: 4.133, 5.260	NA

For peer review only - http://bmjopen.bmj.com/site/about/guidelines.xhtml
For peer review only

Supplementary material

Note: F_PSS denotes F-statistics for Pesaran/Shin/Smith bounds test with null hypothesis of no cointegration. Critical values are retrieved from Narayan PK (2005) for a sample size of n=40 [1]. The null hypothesis of no cointegration is supported if F_PSS statistics is lower than 5% I(0) critical value; the null hypothesis is rejected if F_PSS statistics is greater than 5% I(1) critical value. We applied a conservative approach to considering the number of exposure variables (k), as suggested by Shin Y, et al (2014) [2] (since influenza death rates represent one exposure (k=1), while the analysis actually partitions the exposure to positive and negative changes (k=2)). In a given table, the results of F_PSS for the whole population, men, and women are lower than the reported 5% I(0) critical values that accepts a null hypothesis of no cointegration.

"LnSuicide" indicates that suicide rates were logarithmically-transformed. "Δ" signifies the series is differenced. Signs as "+" and "–" denote the exposure variable being partitioned in positive and negative changes, respectively. "NA" denotes that a certain test or test parameter is not applicable.

Number of lags used for each variable in the model are noted by "lag#". To select the optimal number of lags to be used for choosing p and q parameters for the NARDL (i.e., numbers of lags for dependent and independent variables, respectively), we applied a varsoc command in STATA using the minimal values of Akaike Information Criterion, Schwarz's Bayesian information criterion (SBIC), and the Hannan and Quinn information criterion information criteria. If information criteria indicated different lag orders, SBIC was used to select optimal lags. Thus, for the analysis of the whole population and men, both exposure and outcome time series were lagged once, while for analysis among women, outcome time series were lagged four times and exposure time series were lagged once.

Wald test for asymmetry in a short-term ($Wald_{SR}$) and long-term ($Wald_{LR}$) has a null hypothesis of no cointegration. Q-test for autocorrelation reports the results of Portmanteau test for white noise. Heteroscedasticity is measured by Breusch-Pagan/Cook-Weisberg test. Normality is measured by Jarque-Bera test. RESET statistics refers to the regression specification error tests. CUSUM and CUSUMQ refer to the tests of the cumulative sum of recursive residuals and their squares, respectively. "Stable, no structural break" in the results of CUSUM indicates that the model was found stable and the null hypothesis of no structural break was not rejected (same applies to "Stable" as an output for CUSUMQ).

For peer review only - http://bmjopen.bmj.com/site/about/guidelines.xhtml

Supplementary material

eTable 5. Full specification for non-linear autoregressive distributed lag models used for the analysis of short-term and long-term relationship of suicide rates with positive and negative changes in influenza death rates among the whole population, men, and women in 1957-1978 in Sweden

	Whole population		Men		Women	
	Coefficient (95% CI)	p-value	Coefficient (95% CI)	p-value	Coefficient (95% CI)	p-value
LnSuicide, lag 1	-0.64009 (-1.15104 to -0.12914)	0.018	-0.52238 (-1.11901 to 0.07425)	0.081	-0.66040 (-1.04522 to -0.27558)	0.003
Influenza +, lag 1	0.008034 (-0.02823 to 0.04429)	0.640	-0.01385 (-0.05324 to 0.02553)	0.461	0.02702 (-0.00364 to 0.05768)	0.079
Influenza –, lag 1	0.00661 (-0.02823 to 0.04429)	0.699	-0.01281 (-0.054045 to 0.02841)	0.514	0.01895 (-0.01066 to 0.04857)	0.190
ΔLnSuicide, lag 1	0.14293 (-0.41621 to 0.70206)	0.590	-0.04565 (-0.70111 to 0.60981)	0.883	0.16936 (-0.25995 to 0.59868)	0.409
ΔInfluenza +	-0.00964 (-0.02252 to 0.00324)	0.883	-0.00876 (-0.02738 to 0.00985)	0.328	-0.00976 (-0.02033 to 0.00081)	0.068
ΔInfluenza +, lag 1	-0.01999 (-0.04573 to 0.00574)	0.117	-0.01837 (-0.04871 to 0.01197)	0.214	-0.01298 (-0.04073 to 0.014772)	0.331
ΔInfluenza –	0.00194 (-0.02614 to 0.03003)	0.130	-0.01531 (-0.04658 to 0.01596)	0.309	0.02155 (-0.00343 to 0.04653)	0.085
ΔInfluenza –, lag 1	0.00864 (-0.00271 to 0.02000)	0.124	0.01033 (-0.00294 to 0.02359)	0.116	0.00585 (-0.00565 to 0.01735)	0.292
**Long-term effect**						
Influenza +	0.01255	0.618	-0.02659	0.541	0.04091	0.064
Influenza –	-0.01033	0.684	0.024538	0.582	-0.02870	0.184
**Model diagnostics**						
Q-test for autocorrelation, χ^2	5.662	0.773	3.790	0.925	5.243	0.813

For peer review only - http://bmjopen.bmj.com/site/about/guidelines.xhtml

Supplementary material

Heteroscedasticity, χ^2	0.102	0.750	0.155	0.694	0.079	0.778
Normality, χ^2	1.569	0.456	1.627	0.443	1.237	0.537
RESET, F-statistics	5.839	0.014	3.348	0.064	1.089	0.398
CUSUM	Stable, no structural break	NA	Stable, no structural break	NA	Stable, no structural break	NA
CUSUMQ	Stable	NA	Stable	NA	Stable	NA
Adj. R^2	0.246	NA	0.337	NA	0.371	NA
$Wald_{SR}$, F-statistics	2.903	0.112	0.536	0.477	7.132	0.019
$Wald_{LR}$, F-statistics	0.922	0.354	0.297	0.595	26.74	<0.001
**Cointegration test statistics**						
t_BDM	-2.706	NA	-1.891	NA	-3.707	NA
F_PSS	2.649	NA	2.075	NA	4.688	NA
**Critical values for F_PSS**						
5% critical values; I(0), I(1)	k=1: 5.395, 6.350<br>k=2: 4.267, 5.473	NA	k=1: 5.395, 6.350<br>k=2: 4.267, 5.473	NA	k=1: 5.395, 6.350<br>k=2: 4.267, 5.473	NA

Note: F_PSS denotes F-statistics for Pesaran/Shin/Smith bounds test with null hypothesis of no cointegration. Critical values are retrieved from Narayan PK (2005) for a sample size of n=30 [1]. The null hypothesis of no cointegration is supported if F_PSS statistics is lower than 5% I(0) critical value; the null hypothesis is rejected if F_PSS statistics is greater than 5% I(1) critical value. We applied a conservative approach to considering the number of exposure variables (k), as suggested by Shin Y, et al (2014) [2] (since influenza death rates represent one exposure (k=1), while the analysis actually partitions the exposure to positive and negative changes (k=2)). In a given table, the results of F_PSS for the whole population, men, and women are lower than the reported 5% I(0) critical values that accepts a null hypothesis of no cointegration.

For peer review only - http://bmjopen.bmj.com/site/about/guidelines.xhtml

Supplementary material

"LnSuicide" indicates that suicide rates were logarithmically-transformed. "Δ" signifies the series is differenced. Signs as "+" and "–" denote the exposure variable being partitioned in positive and negative changes, respectively. "NA" denotes that a certain test or test parameter is not applicable.

Number of lags used for each variable in the model are noted by "lag#". To select the optimal number of lags to be used for choosing p and q parameters for the NARDL (i.e., numbers of lags for dependent and independent variables, respectively), we applied a varsoc command in STATA using the minimal values of Akaike Information Criterion, Schwarz's Bayesian information criterion (SBIC), and the Hannan and Quinn information criterion information criteria. If information criteria indicated different lag orders, SBIC was used to select optimal lags. Thus, for the analysis of the whole population, men, and women, both exposure and outcome time series were lagged once.

Wald test for asymmetry in a short-term ($Wald_{SR}$) and long-term ($Wald_{LR}$) has a null hypothesis of no cointegration. Q-test for autocorrelation reports the results of Portmanteau test for white noise. Heteroscedasticity is measured by Breusch-Pagan/Cook-Weisberg test. Normality is measured by Jarque-Bera test. RESET statistics refers to the regression specification error tests. CUSUM and CUSUMQ refer to the tests of the cumulative sum of recursive residuals and their squares, respectively. "Stable, no structural break" in the results of CUSUM indicates that the model was found stable and the null hypothesis of no structural break was not rejected (same applies to "Stable" as an output for CUSUMQ).

REFERENCES

1 Narayan PK. The saving and investment nexus for China: evidence from cointegration tests. *Applied Economics* 2005; **37**(17): 1979–1990.
2 Shin Y, Yu B, Greenwood-Nimmo M. Modelling Asymmetric Cointegration and Dynamic Multipliers in a Nonlinear ARDL Framework. In: Sickles RC, Horrace WC, eds. Festschrift in Honor of Peter Schmidt: Econometric Methods and Applications. New York: Springer Science+Business Media, 2014: 281–314.

For peer review only - http://bmjopen.bmj.com/site/about/guidelines.xhtml

STROBE Statement—checklist of items that should be included in reports of observational studies

	Item No	Recommendation	Page No
**Title and abstract**	1	(*a*) Indicate the study's design with a commonly used term in the title or the abstract	1
		(*b*) Provide in the abstract an informative and balanced summary of what was done and what was found	2
**Introduction**			
Background/rationale	2	Explain the scientific background and rationale for the investigation being reported	3
Objectives	3	State specific objectives, including any prespecified hypotheses	3
**Methods**			
Study design	4	Present key elements of study design early in the paper	3-4
Setting	5	Describe the setting, locations, and relevant dates, including periods of recruitment, exposure, follow-up, and data collection	3-5
Participants	6	(*a*) *Cohort study*—Give the eligibility criteria, and the sources and methods of selection of participants. Describe methods of follow-up *Case-control study*—Give the eligibility criteria, and the sources and methods of case ascertainment and control selection. Give the rationale for the choice of cases and controls *Cross-sectional study*—Give the eligibility criteria, and the sources and methods of selection of participants	3-5
		(*b*) *Cohort study*—For matched studies, give matching criteria and number of exposed and unexposed *Case-control study*—For matched studies, give matching criteria and the number of controls per case	n/a
Variables	7	Clearly define all outcomes, exposures, predictors, potential confounders, and effect modifiers. Give diagnostic criteria, if applicable	3-4
Data sources/ measurement	8*	For each variable of interest, give sources of data and details of methods of assessment (measurement). Describe comparability of assessment methods if there is more than one group	3-4
Bias	9	Describe any efforts to address potential sources of bias	4
Study size	10	Explain how the study size was arrived at	4
Quantitative variables	11	Explain how quantitative variables were handled in the analyses. If applicable, describe which groupings were chosen and why	5
Statistical methods	12	(*a*) Describe all statistical methods, including those used to control for confounding	5
		(*b*) Describe any methods used to examine subgroups and interactions	5
		(*c*) Explain how missing data were addressed	n/a
		(*d*) *Cohort study*—If applicable, explain how loss to follow-up was addressed *Case-control study*—If applicable, explain how matching of cases and controls was addressed *Cross-sectional study*—If applicable, describe analytical methods taking account of sampling strategy	n/a
		(*e*) Describe any sensitivity analyses	n/a

Continued on next page

For peer review only - http://bmjopen.bmj.com/site/about/guidelines.xhtml

**Results**			
Participants	13*	(a) Report numbers of individuals at each stage of study—eg numbers potentially eligible, examined for eligibility, confirmed eligible, included in the study, completing follow-up, and analysed	n/a
		(b) Give reasons for non-participation at each stage	n/a
		(c) Consider use of a flow diagram	n/a
Descriptive data	14*	(a) Give characteristics of study participants (eg demographic, clinical, social) and information on exposures and potential confounders	5-7
		(b) Indicate number of participants with missing data for each variable of interest	n/a
		(c) *Cohort study*—Summarise follow-up time (eg, average and total amount)	n/a
Outcome data	15*	*Cohort study*—Report numbers of outcome events or summary measures over time	6-7
		Case-control study—Report numbers in each exposure category, or summary measures of exposure	
		Cross-sectional study—Report numbers of outcome events or summary measures	
Main results	16	(*a*) Give unadjusted estimates and, if applicable, confounder-adjusted estimates and their precision (eg, 95% confidence interval). Make clear which confounders were adjusted for and why they were included	5-7, and 13-15
		(*b*) Report category boundaries when continuous variables were categorized	
		(*c*) If relevant, consider translating estimates of relative risk into absolute risk for a meaningful time period	
Other analyses	17	Report other analyses done—eg analyses of subgroups and interactions, and sensitivity analyses	
**Discussion**			
Key results	18	Summarise key results with reference to study objectives	7
Limitations	19	Discuss limitations of the study, taking into account sources of potential bias or imprecision. Discuss both direction and magnitude of any potential bias	8
Interpretation	20	Give a cautious overall interpretation of results considering objectives, limitations, multiplicity of analyses, results from similar studies, and other relevant evidence	7-8
Generalisability	21	Discuss the generalisability (external validity) of the study results	8
**Other information**			
Funding	22	Give the source of funding and the role of the funders for the present study and, if applicable, for the original study on which the present article is based	9

*Give information separately for cases and controls in case-control studies and, if applicable, for exposed and unexposed groups in cohort and cross-sectional studies.

Note: An Explanation and Elaboration article discusses each checklist item and gives methodological background and published examples of transparent reporting. The STROBE checklist is best used in conjunction with this article (freely available on the Web sites of PLoS Medicine at http://www.plosmedicine.org/, Annals of Internal Medicine at http://www.annals.org/, and Epidemiology at http://www.epidem.com/). Information on the STROBE Initiative is available at www.strobe-statement.org.

For peer review only

A Swedish nationwide time series analysis of influenza and suicide deaths from 1910 to 1978

Journal:	*BMJ Open*
Manuscript ID	bmjopen-2021-049302.R1
Article Type:	Original research
Date Submitted by the Author:	13-May-2021
Complete List of Authors:	Rück, Christian; Karolinska Institutet, Department of Clinical Neuroscience, Center for Psychiatry Research<br>Mataix-Cols, David; Karolinska Institutet, Department of Clinical Neuroscience, Center for Psychiatry Research<br>Malki, Kinda; Karolinska Institutet<br>Adler, Mats; Karolinska Institutet<br>Flygare, Oskar; Karolinska Institutet<br>Runeson, Bo; Karolinska Institutet, Clin Neuroscience<br>Sidorchuk, Anna; Karolinska Institutet Department of Clinical Neuroscience,
**Primary Subject Heading**:	Mental health
Secondary Subject Heading:	Epidemiology, Public health
Keywords:	MENTAL HEALTH, COVID-19, Suicide & self-harm < PSYCHIATRY

SCHOLARONE™
Manuscripts

I, the Submitting Author has the right to grant and does grant on behalf of all authors of the Work (as defined in the below author licence), an exclusive licence and/or a non-exclusive licence for contributions from authors who are: i) UK Crown employees; ii) where BMJ has agreed a CC-BY licence shall apply, and/or iii) in accordance with the terms applicable for US Federal Government officers or employees acting as part of their official duties; on a worldwide, perpetual, irrevocable, royalty-free basis to BMJ Publishing Group Ltd ("BMJ") its licensees and where the relevant Journal is co-owned by BMJ to the co-owners of the Journal, to publish the Work in this journal and any other BMJ products and to exploit all rights, as set out in our licence.

The Submitting Author accepts and understands that any supply made under these terms is made by BMJ to the Submitting Author unless you are acting as an employee on behalf of your employer or a postgraduate student of an affiliated institution which is paying any applicable article publishing charge ("APC") for Open Access articles. Where the Submitting Author wishes to make the Work available on an Open Access basis (and intends to pay the relevant APC), the terms of reuse of such Open Access shall be governed by a Creative Commons licence – details of these licences and which Creative Commons licence will apply to this Work are set out in our licence referred to above.

Other than as permitted in any relevant BMJ Author's Self Archiving Policies, I confirm this Work has not been accepted for publication elsewhere, is not being considered for publication elsewhere and does not duplicate material already published. I confirm all authors consent to publication of this Work and authorise the granting of this licence.

For peer review only

Word count

Abstract: 231

Body: 3532

Tables: 3

Figures: 2

A Swedish nationwide time series analysis of influenza and suicide deaths from 1910 to 1978

Christian Rück, M.D.[1], David Mataix-Cols, Ph.D.[1], Kinda Malki, M.D. [1], Mats Adler, M.D. [1], Oskar Flygare, M.Sc.[1], Bo Runeson, M.D. [1], Anna Sidorchuk, M.D.[1]

Corresponding author:
Professor Christian Rück, M46, Karolinska University Hospital Huddinge, SE-14186 Huddinge, Sweden E-mail: Christian.ruck@ki.se, telephone +46704843392.

Affiliation:
1. Centre for Psychiatry Research, Department of Clinical Neuroscience, Karolinska Institutet, & Stockholm Health Care Services, Region Stockholm, Sweden

For peer review only

ABSTRACT

Objectives: There is concern that the COVID-19 pandemic will be associated to a surge of suicides, but evidence supporting a link between pandemics and suicide is limited. Using data from the three influenza pandemics of the 20th century, we aimed to investigate whether an association exists between influenza deaths and suicide deaths.

Design: Time series analysis.

Setting: Sweden.

Participants: Deaths from influenza and suicides extracted from the Statistical Yearbook of Sweden for 1910-1978, covering three pandemics (the Spanish flu, the Asian flu and the Hong-Kong flu).

Main outcome measures: Annual suicide rates in Sweden among the whole population, men, and women. Non-linear autoregressive distributed lag (NARDL) models was implemented to explore if there is a short-term and/or long-term relationship of increases and decreases in influenza death rates with suicide rates during 1910-1978.

Results: Between 1910-1978, there was no evidence of either short-term or long-term significant associations between influenza death rates and changes in suicides (β-coefficients of 0.00002, P=0.931 and β=0.00103, P=0.764 for short-term relationship of increases and decreases in influenza death rates, respectively, with suicide rates, and β=-0.0002, P=0.998 and β=0.00211, P=0.962 for long-term relationship of increases and decreases in influenza death rates, respectively, with suicide rates). The same pattern emerged in separate analyses for men and women.

Conclusions: We found no evidence of short or long-term association between influenza death rates and suicide death rates across three 20th century pandemics.

Key words: pandemics, suicide, mental health, Spanish flu, Asian influenza, Hong-Kong influenza, COVID-19

Strengths and limitations of this study

- To our knowledge, this is the first study to investigate the association between influenza deaths and suicide across several pandemics.
- The large amount of nationwide data on influenza and suicide death rates covering 1910-1978 is a strength of the study.
- To guard against effects of changes in the recording of causes of death, we created a series of dummy variables for each corresponding period but no significant effects were found.
- No historical data with higher temporal resolution than yearly data could affect the results if the time sequence of association between changes in influenza death and suicide differ from the chosen time interval.

For peer review only

INTRODUCTION

Various international surveys have documented a negative impact of the COVID-19 pandemic on the population's mental health, with increased levels of psychological stress, psychiatric symptoms, insomnia, and alcohol consumption.[1–4] Whether these acute impacts will persist long term is currently unknown. The Royal College of Psychiatrists in the UK and the World Health Organization as well as the International Academy of Suicide Research have raised concerns of a possible increase in suicide rates.[5–7] Such concerns originate from a combination of known risk factors for suicide, including the impacts of social distancing and disconnectedness, an economic downturn, the decreased access to mental health services, and increased access to lethal means exemplified by an increase in gun purchases in the US.[8] For example, a study of the economic recession in USA 2007-2009 found that for every percentage point increase in the unemployment rate, there was about a 1.6 per cent increase in the suicide rate.[9,10] These findings have been questioned. A study using an interrupted time-series analysis taking e.g. seasonality and long-term trends into account, found little evidence that the recession resulted in net excess suicides across all age and gender groups.[11] However, the was some evidence of excess suicides among men aged 65 and above 65 and young women. Another study failed to find an increase in suicide rates in Sweden during the two most recent economic recessions.[12]

While the concern is widespread, there is currently little evidence to support a clear association between the ongoing COVID-19 outbreak and an increased risk of suicide. Data from the US Centers for Disease Control and Prevention shows that suicide decreased in 2020, compared to the preceding year.[13] Data from April to October 2020 from the UK did not show an increase of suicides.[14] The first months of the COVID-19 pandemic in 21 countries was studied by Pirkis and collegues and overall there was no support for an increase in suicides.[15] Other reports highlight that the outcomes differ and that certain minorities may be at higher risk.[16]

Historic US mortality data from the largest pandemic in the 20th century, the Spanish flu, showed that the Spanish flu was associated with an increase in suicides but those effects may have been mitigated by a decline in alcohol consumption.[17] Suicide during the same pandemic in Taiwan, where the Japanese Colonial Government implemented physical distancing, school closings and prohibited religious activities, was studied in a paper by Chang and collegues.[18] They reported a small and shortlived increase of suicides in the second wave of the pandemic. A study of the impact of social distancing measures on suicide in 1918 in the US was studied and showed that increasing social distancing was associated with suicide rates independent of the influenza mortality rate.[19] In Sweden, there were measures in place in Sweden during the Spanish Flu to minimize the spread of the disease, such as school closings, and public gatherings, cinemas and religious meetings were temporarily stopped in some places.[20]

The outbreak of the Severe Acute Respiratory Syndrome (SARS) epidemic in 2003 in Hong Kong was according to two studies associated with an increase of suicide in the population above the age of 65.[21,22] In the first of these two reports, the association was only statistically significant in elderly women.[21] Another study of suicides during SARS suggested that disconnectedness and fear of contracting SARS were more prevalent in older

For peer review only - http://bmjopen.bmj.com/site/about/guidelines.xhtml

adult SARS-related suicide victims than non-SARS-related suicide victims. However, this was based on a small number of suicides.[23] To summarise, at present, our understanding of the effects of pandemics on suicide rates is very limited.

The availability of high-quality historical data on mortality due to influenza and suicide in Sweden provides a unique opportunity to examine this important question. While we at present have some short-term data on the rates of suicide during the COVID-19 pandemic, a better understanding of what may be the outcome in the longer run would be valuable. Using historical data from the three major influenza pandemics of the 20th century, we aimed to formally investigate whether an association between influenza deaths and suicide deaths exists. We hypothesize that such association, if any, would likely be weak. Given the substantial sex differences in suicidal behaviour and the studies suggesting sex-specific effects during SARS,[21] we additionally report on associations separately in men and women.

METHODS

Data acquisition

Annual data on influenza death rates and suicide rates were estimated based on information from the Statistical Yearbook of Sweden from 1910-1978.[24] Over this period, Sweden was hit by three influenza pandemics which occurred during different socio-political contexts, namely the Spanish flu (1918-1920, with the first case appearing in Sweden in June 1918), the Asian flu (1957-1958, being first documented in August 1957), and the Hong Kong flu (1968-1969, starting in the autumn of 1968).[20] The yearbooks were produced by Statistics Sweden, a governmental agency responsible for the official statistics in Sweden, with a history of population statistics going back to the 18th century.[24] For each year from 1910 to 1978, we retrieved information on the total population of Sweden, the number of deaths by influenza (if death cause was indicated as "influenza") and the number of suicides (if death cause was indicated as "suicide"), as well as the corresponding data separately for men and women. Since yearbooks reported data retrospectively for several years prior to the year each book was published, we checked the correctness of retrieved data by comparing yearbooks with overlapping reporting periods. We constructed influenza mortality rates and suicide rates per 100 000 inhabitants for each year by dividing the number of deaths by influenza and, separately, suicide, by the total number of individuals registered in Sweden in a corresponding year and then multiplying by 100 000. The corresponding rates for men and women were constructed likewise. In addition, we collected information on the changes in registration of deaths in Sweden that included the cause of death classification based on the Bertillon criteria (prior to 1931), the new classification introduced in cooperation with statistical authorities from other Nordic countries (1931-1950), the International Classification of Diseases (ICD) Sixth Revision (ICD-6; 1951-1957), ICD-7 (1958-1968), and ICD-8 (1969-1978).[25] To capture a potential effect of changes in classification, we created a series of dummy variables for each corresponding period, but these were only kept in the models if statistically significant.

The study protocol was not preregistered. The full dataset used in the analysis of historical data (1910-1978) and the STATA code are available in the online Supplement (**Supplementary eTable 1** and **Supplementary material**).

Statistical Analyses

For peer review only - http://bmjopen.bmj.com/site/about/guidelines.xhtml

We used the historical data 1910-1978 that includes three pandemics and focused on exploring a possible asymmetric short-term effect of influenza death rates on suicide (i.e., immediate or instantaneous effect) and long-term effect (i.e., if the relationship of exposure and outcome has a lag-structure, or in other words, if the effect of exposure on outcome is distributed over a longer period of time) by applying non-linear autoregressive distributed lag (NARDL) models,[26] a technique initially introduced for research in economics. NARDL models split the exposure in partial sum of positive changes (i.e, increases in influenza death rates) and partial sum of negative changes (i.e., decreases in influenza death rates) and explore if an outcome responds differently (i.e., asymmetrically) to an increase and decrease in exposure variable.[26] In other words, NARDL models do not rely on an assumption of symmetrical effects, by which the association of the outcome with a unit of positive change in the exposure is expected to be equal in strength and opposite in direction to the association between the outcome and a unit of negative change in the exposure. We performed the modelling by using the *nardl* command in STATA.

Before the NARDL model is executed, it is important to test that the conditions for the modelling are fulfilled. It starts from testing whether or not the variables are stationary (i.e., their means and variances are constant over time). The advantage of the NARDL modelling is that it can be used regardless of whether the variables are integrated of the order zero, which means that variables are stationary, integrated of the order one, which indicates non-stationarity, or mixed.[26] We examined the stationarity properties of the variables by using the Augmented Dickey–Fuller unit-root test (ADF) and Kwiatkowski-Phillips-Schmidt-Shin test for stationarity (KPSS) to explore the order of the integration for influenza death rates and suicide rates separately for the total population, men, and women. Previous studies on suicides hypothesized that various suicidogenic factors may interplay and reinforce each other and the logarithmic transformation of suicide time series was suggested.[27,28] We made such transformation by expressing suicide rates in natural logarithm. To select the optimal number of lags (i.e., how far in the past the dependency among measurements is examined) to be used in the NARDL for dependent and independent variables, we applied the *varsoc* command in STATA using the minimal values of Akaike Information Criterion, Schwarz's Bayesian information criterion (SBIC), and the Hannan and Quinn information criterion information criteria. If information criteria indicated different lag orders, SBIC was used to select optimal lags. We applied the NARDL model to (i) estimate the coefficients and the corresponding 95% confidence intervals for short-term and long-term association of suicide rates with an increase and decrease in influenza death rates; (ii) explore if there is a long-term cointegration between exposure and outcome by using a bounds test (if variables are cointegrated, it means their positive and negative components do not drift far away from each other in the longer term); and (iii) obtain Wald test statistics that specifies whether or not short-term and/or long-term relation between the exposure and outcome is asymmetrical. Model diagnostic tests are described in the **Supplementary material**.

The modelling was performed, first, for the whole period of 1910-1978, and then for the periods of 1918-1956 (from the beginning of the Spanish influenza pandemic to the year before the Asian influenza pandemic), and for 1957-1978 (from the beginning of Asian pandemic to the end of observation period; with this interval also covering the Hong Kong influenza pandemic).

All statistical analyses were performed using STATA version 15.1 (StataCorp LLC, College Station, TX, USA).

For peer review only - http://bmjopen.bmj.com/site/about/guidelines.xhtml

Patient and public involvement

Patients and members of the public were not directly involved as this study used publically available historical national mortality data.

RESULTS

Over the period 1910-1978, influenza death rates fluctuated considerably in Sweden with the highest rates being observed during the Spanish flu (in 1918, 1919, and 1920, the rates were 470.93, 125.55, and 48.32 per 100 000 inhabitants, respectively) (**Figure 1**). In post-pandemic years, several noticeable peaks in influenza death rates appeared, with the ones in 1922, 1927, 1929, 1931, 1937, and 1941 being particularly high. In the following years, a considerable fluctuation in influenza mortality remained, although the rates during the periods of the Asian flu (in 1957 and 1958: 8.78 and 3.99 per 100 000 inhabitants, respectively) and the Hong Kong flu (in 1968 and 1969: 3.67 and 4.68 per 100 000 inhabitants, respectively) were lower than that in the first half of the century. The influenza death rates were very similar in men and women across the entire observation period (**Figure 2**).

[Figure 1 to be inserted here]

[Figure 2 to be inserted here]

During the same period, a total of 80 058 deaths due to suicide occurred in Sweden (60 713 in men and 19 345 in women). The average suicide rate across 1910-1978 was 16.79 per 100 000 inhabitants (standard deviation [SD] of 2.58), with corresponding rates for men and women as 25.85 (SD=3.37) and 7.91 (SD=2.26) per 100 000, respectively. There was a considerable fluctuation in the suicide rates over time (**Figure 1**). The initial decrease in suicide rates during 1913-1918, with the lowest rate of 9.97 per 100 000 in 1918, was followed by an increase with the highest peak of 22.15 per 100 000 reached in 1970, with a series of intermediate peaks.

Sex-specific suicide rates differed considerably with the rates among men being between twice to over four times higher than that in women (on average, 3.4-times higher) (**Figure 2**). Moreover, suicide rates in men exhibited a sharp dip in 1913-1918, which was first followed by an increase by 1921 and then continued to raise more gradually, although with several distinct peaks and, rarely, dips. Among women, an upward trend in suicide rate was present, although with less fluctuations than that in men.

In our analysis of 1910-1978 data, the first step with the use of the ADF and KPSS test statistics on variables' stationarity indicated that the logarithmically-transformed suicide rates were integrated of the order one (for all, and men and women separately); whereas the influenza death rates were integrated of the order zero (for all and for men and women separately) (**Supplementary eTable 2)**. On this premise, we implemented the NARDL modelling. Dummy variables on changes in death registration did neither appear statistically significant nor affected any parameter estimates in the initial model, and thus were not included in the final models. As reported in **Table 1**, for the observation period of 1910-1978, there were no statistically significant associations (either short-term or long-term) between decreased or increased influenza death rates and suicide rates among the overall population and among men. The corresponding results for women indicated a possible short-term association whereby a decrease in influenza rates seemed to be associated with a borderline

For peer review only - http://bmjopen.bmj.com/site/about/guidelines.xhtml

decrease in suicides; however, the findings were not supported by the Wald test for asymmetry ([$Wald_{SR}$], i.e. the null hypothesis of $Wald_{SR}$ that an increase and decrease in influenza death rates would symmetrically affect suicide rates was not rejected). This suggests that association was likely due to the effect of other unobserved factors that may influence suicides in women. Full specification of the models is reported in **Supplementary eTable 3**. Full specification also includes the results of testing for long-term cointegration between influenza mortality and suicides (as reported by F-statistics for Pesaran/Shin/Smith bounds test [F_PSS]). Long-term cointegration was not found as we were unable to reject the null hypothesis of no cointegration between variables (**Supplementary eTable 3,** see footnotes for details on bounds test for cointegration).

The results of the additional analyses for the periods 1918-1956 and 1957-1978 also failed to provide clear evidence of association between changes in influenza mortality rates and suicide rates in either short-term or long-term. However, in the analysis among women, a long-term asymmetry was suggested in both periods (the Wald test for asymmetry in the long-term [$Wald_{LR}$] $p=0.044$ and $p<0.001$ in the analyses of 1918-1956 and 1957-1978, respectively) and a short-term asymmetry in the analysis of 1957-1978 ($Wald_{SR}$ test $p=0.019$) (**Tables 2** and **3**). We assume that these findings may again reflect the influence of unobserved confounders, as these results were not supported by other coefficients, which correspond to long- and short-term associations between increases and decreases in influenza deaths and suicides among women in 1918-1956 and 1957-1978 (**Tables 2** and **3**). It is important to mention that the results for 1957-1978 should be considered with caution since the analysis included time series with only 22 observations. Full specifications for the models used in the analyses of 1918-1956 and 1957-1978 are reported in **Supplementary eTable 4** and **5**. No evidence of long-term cointegration between influenza and suicide rates were found in either period (F_PSS statistics does not reject the null hypothesis in the analyses of the whole population, men, and women; **Supplementary eTable 4** and **5,** see footnotes for details on bounds test for cointegration). Overall, the model diagnostics for all models for 1910-1978, 1918-1956, and 1957-1978 time series support the validity of the results since, with very few exceptions, all diagnostic tests (for details see Supplementary materials) are insignificant indicating that there is no autocorrelation, heteroscedasticity, misspecification and non-normality In addition, the tests of the cumulative sum of recursive residuals and their squares (CUSUM and CUSUMQ, respectively) for all models indicate stability and absence of structural breaks (for details see footnotes for **Table 1-3** and **Supplementary eTable 3-5**).

For peer review only

DISCUSSION

This study used publicly available Swedish national data from 1910 to 1978 to shed light on the potential association between influenza-related deaths and over 80 000 deaths by suicide across the three 20th century pandemics. To our knowledge, this is the first study of influenza death and suicide that analyses data from several influenza pandemics. The full modelling provided no clear evidence of either a short-term or a long-term relationship between changes in influenza death rates and changes in suicide rates. The year with the highest number of influenza-related deaths by far, 1918, had the lowest number of suicides in the whole time series.

The findings in context

For peer review only - http://bmjopen.bmj.com/site/about/guidelines.xhtml

International historical data on pandemics and suicide are very scarce. A report from the US focusing on the years 1910-1920 and using monthly data suggested a possible association between influenza deaths and suicide but because the data did not go beyond the last year of that pandemic, it does not inform us about any longer term effects.[17] Strengths of that study included the use of monthly data and the use of time series analysis, rather than just comparing suicide rates before and after the pandemic. Chang et al studied suicides during the same pandemic in Taiwan, not using a time series analysis but also using monthly data from 1918-20 and found an increase during the few months of the second vawe of the pandemic but that the effect was short-lived. As our study used yearly and not monthly data, it could not confirm or disconfirm the findings of the Taiwanese study. Our study expands and improves upon previous investigations because it allowed us to examine the long-term associations between influenza and suicide deaths. A study focusing on SARS and suicide found some evidence of sex-specific effects in the short-term.[21] As suicide rates varied considerably in the years preceding SARS in Hong Kong, it is difficult to be certain that SARS, and not other contributing factors, was causally associated with an increase in suicides. In the present study, which uniquely spanned over several decades, we found that while suicide was consistently more prevalent among men throuought the study period, there were no sex-specific associations to influenza death.

Meaning of the study

What do these results mean for our understanding of the short- and long-term consequences of influenza pandemics? Our results do not support the belief that global pandemics necessarily lead to a substantial increase in suicides. There are many factors associated with a global pandemic that may potentially increase suicide risk in the population, but these may be offset by other protective factors that should not be overlooked. A shared sense of belonging and focus, social connectedness and a "pulling together effect" may be one such factor.[1,29] We acknowledge that these historical findincs may not be directly applicable to the current COVID-19 pandemic because there are several important differences between the previous and current pandemics, such as globalisation, a different socio-political context and the impact of social media, to name a few.

Strengths and limitations

The major strengths of this study were the use of a large amount of historical national data across three 20th century pandemics and state of the art time-series analyses. A limitation is that all data comes from a single country and and thus caution is needed when generalizing the study findings to countries with different level of development of clinical and preventive medicine, social support, etc.The study used the Swedish historical public death records and we had no means of verifying the causes of death. The coverage and precision of these records is likely to have improved over time for both variables. To guard against effects of changes in the recording of causes of death, we created a series of dummy variables for each corresponding period but no significant effects of those were found. If there were some other time-varying factors that could affect the coverage and precision of death records, apart from the official changes in registration system, this might have biased our results, in particular if such factors differentially affected the quality of recording deaths due to influenza and suicide. As we did not have access to data with higher temporal resolution than yearly data, that could affect our results if the time sequence of association between changes in influenza death and suicide differ from the chosen time interval. A multitude of factors may vary that impact the resilience of the society with regards to the effect of a pandemic. Such factors may also vary over time and place. However, the fact that we observed no clear associations between influenza and suicide deaths across pandemics, which challenged society with

For peer review only - http://bmjopen.bmj.com/site/about/guidelines.xhtml

various degrees of e.g. economic effects and health care supply issues, does support the stability of our findings.

Conclusion
In this national analysis of historical data spanning across three 20th century pandemics, we found no evidence of a short- or long-term association between influenza death rates and suicide rates. The results challenge the notion that an increase of suicides follows as a certain consequence of a pandemic. Future research on the effect of the current COVID-19 pandemic should explore the possibility of differential short- and long-term effects on suicide rates in different subgroups of the population.

Footnotes

Contributor statement: CR and AS have full access to all the data in this study and take full responsibility as guarantors for the integrity of the data and the accuracy of the data analysis. CR, DMC, and AS conceived and designed the study. CR, KM, MA, and AS performed data collection. AS undertook the statistical analysis. CR, DMC, and AS drafted the manuscript. CR, AS, DMC, KM, MA, BR and OF provided critical input to the analyses, interpreted the data, and revised the manuscript critically. The corresponding author confirms that all listed authors meet authorship criteria and that no others meeting the criteria have been omitted.

Funding: Karolinska Institutet and Region Stockholm, which provide salary for the authors, had no involvement in the study design; collection, analysis, and interpretation of data; in the writing of the report; or in the decision to submit the paper for publication.

Competing interests: All authors have completed the ICMJE uniform disclosure form at www.icmje.org/coi_disclosure.pdf and declare: no grant support from any organisation for the submitted work, salaries of the author were provided from Region Stockholm and Karolinska Institutet but not specifically for this project; no financial relationships with any organisations that might have an interest in the submitted work in the previous three years; no other relationships or activities that could appear to have influenced the submitted work.

Data sharing: The full dataset (numbers of deaths by influenza and suicide and total population for 1910-1978) is published as supplementary material (eTable 1).

Dissemination to participants and patient organizations: since only aggregated mortiality data was used, dissemination to participants is not possible. We intend to disseminate the results to the general public via media and the research groups webpage.

Transparency declaration: The lead author affirms that this manuscript is an honest, accurate, and transparent account of the study being reported; that no important aspects of the study have been omitted; and that any discrepancies from the study as planned have been explained.

Ethical approval: Since only data already available in the public domain were used, ethical approval was not necessary.

For peer review only

References

1 Reger MA, Stanley IH, Joiner TE. Suicide Mortality and Coronavirus Disease 2019—A Perfect Storm? *JAMA Psychiatry* Published Online First: 10 April 2020. doi:10.1001/jamapsychiatry.2020.1060

2 Dutheil F, Mondillon L, Navel V. PTSD as the second tsunami of the SARS-Cov-2 pandemic. *Psychol Med* 2020;:1–2. doi:10.1017/S0033291720001336

3 O'Connor RC, Wetherall K, Cleare S, *et al.* Mental health and well-being during the COVID-19 pandemic: longitudinal analyses of adults in the UK COVID-19 Mental Health & Wellbeing study. *Br J Psychiatry* 2020;:1–8. doi:10.1192/bjp.2020.212

4 Pierce M, Hope H, Ford T, *et al.* Mental health before and during the COVID-19 pandemic: a longitudinal probability sample survey of the UK population. *Lancet Psychiatry* 2020;**7**:883–92. doi:10.1016/S2215-0366(20)30308-4

5 WHO. Substantial investment needed to avert mental health crisis. https://www.who.int/news-room/detail/14-05-2020-substantial-investment-needed-to-avert-mental-health-crisis (accessed 1 Jun 2020).

6 Zalsman G, Stanley B, Szanto K, *et al.* Suicide in the Time of COVID-19: Review and Recommendations. *Arch Suicide Res* 2020;:1–6. doi:10.1080/13811118.2020.1830242

7 Psychiatrists see alarming rise in patients needing urgent and emergency care and forecast a 'tsunami' of mental illness. www.rcpsych.ac.uk. https://www.rcpsych.ac.uk/news-and-features/latest-news/detail/2020/05/15/psychiatrists-see-alarming-rise-in-patients-needing-urgent-and-emergency-care (accessed 1 Jun 2020).

8 Moutier C. Suicide Prevention in the COVID-19 Era: Transforming Threat Into Opportunity. *JAMA Psychiatry* Published Online First: 16 October 2020. doi:10.1001/jamapsychiatry.2020.3746

9 Stuckler D, Basu S, Suhrcke M, *et al.* The public health effect of economic crises and alternative policy responses in Europe: an empirical analysis. *The Lancet* 2009;**374**:315–23. doi:10.1016/S0140-6736(09)61124-7

10 Phillips JA, Nugent CN. Suicide and the Great Recession of 2007–2009: The role of economic factors in the 50 U.S. states. *Soc Sci Med* 2014;**116**:22–31. doi:10.1016/j.socscimed.2014.06.015

11 Harper S, Bruckner TA. Did the Great Recession increase suicides in the USA? Evidence from an interrupted time-series analysis. *Ann Epidemiol* 2017;**27**:409-414.e6. doi:10.1016/j.annepidem.2017.05.017

12 Reeves A, McKee M, Stuckler D. Economic suicides in the Great Recession in Europe and North America. *Br J Psychiatry* 2014;**205**:246–7. doi:10.1192/bjp.bp.114.144766

13 Ahmad FB, Anderson RN. The Leading Causes of Death in the US for 2020. *JAMA* Published Online First: 31 March 2021. doi:10.1001/jama.2021.5469

14 Appleby L, Richards N, Ibrahim S, *et al.* Suicide in England in the COVID-19 pandemic: Early observational data from real time surveillance. *Lancet Reg Health - Eur* 2021;:100110. doi:10.1016/j.lanepe.2021.100110

15 Pirkis J, John A, Shin S, *et al.* Suicide trends in the early months of the COVID-19 pandemic: an interrupted time-series analysis of preliminary data from 21 countries. *Lancet Psychiatry* Published Online First: 13 April 2021. doi:10.1016/S2215-0366(21)00091-2

For peer review only

16 Mitchell TO, Li L. State-Level Data on Suicide Mortality During COVID-19 Quarantine: Early Evidence of a Disproportionate Impact on Racial Minorities. *Psychiatry Res* 2021;**295**:113629. doi:10.1016/j.psychres.2020.113629
17 Wasserman IM. The impact of epidemic, war, prohibition and media on suicide: United States, 1910-1920. *Suicide Life Threat Behav* 1992;**22**:240–54.
18 Chang Y-H, Chang S-S, Hsu C-Y, *et al.* Impact of Pandemic on Suicide: Excess Suicides in Taiwan During the 1918–1920 Influenza Pandemic. *J Clin Psychiatry* 2020;**81**. doi:10.4088/JCP.20l13454
19 Stack S, Rockett IRH. Social distancing predicts suicide rates: Analysis of the 1918 flu pandemic in 43 large cities, research note. *Suicide Life Threat Behav* 2021;:sltb.12729. doi:10.1111/sltb.12729
20 Holmberg M. The ghost of pandemics past: revisiting two centuries of influenza in Sweden. *Med Humanit* 2017;**43**:141–7. doi:10.1136/medhum-2016-011023
21 Chan SMS, Chiu FKH, Lam CWL, *et al.* Elderly suicide and the 2003 SARS epidemic in Hong Kong. *Int J Geriatr Psychiatry* 2006;**21**:113–8. doi:10.1002/gps.1432
22 Cheung YT, Chau PH, Yip PSF. A revisit on older adults suicides and Severe Acute Respiratory Syndrome (SARS) epidemic in Hong Kong. *Int J Geriatr Psychiatry* 2008;**23**:1231–8. doi:10.1002/gps.2056
23 Yip PSF, Cheung YT, Chau PH, *et al.* The Impact of Epidemic Outbreak: The Case of Severe Acute Respiratory Syndrome (SARS) and Suicide Among Older Adults in Hong Kong. *Crisis* 2010;**31**:86–92. doi:10.1027/0227-5910/a000015
24 *Statistics Sweden. Statistical yearbook of Sweden [Statistisk årsbok för Sverige 1914–2014]. https://www.scb.se/hitta-statistik/aldre-statistik/innehall/statistisk-arsbok-1914-2014/.*
25 The National Board of Health and Welfare. Cause of death statistics. History, production methods and reliability [Dödsorsaksstatistik. Historik, produktionsmetoder och tillförlitlighet]. www.socialstyrelsen.se. 2010.
26 Shin Y, Yu B, Greenwood-Nimmo M. Modelling Asymmetric Cointegration and Dynamic Multipliers in a Nonlinear ARDL Framework. In: Sickles RC, Horrace WC, eds. *Festschrift in Honor of Peter Schmidt: Econometric Methods and Applications*. New York: : Springer Science+Business Media 2014. 281–314.
27 Norstrom T, Stickley A, Shibuya K. The importance of alcoholic beverage type for suicide in Japan: a time-series analysis, 1963-2007. *Drug Alcohol Rev* 2012;**31**:251–6. doi:10.1111/j.1465-3362.2011.00300.x
28 Gunnell D, Lopatatzidis A, Dorling D, *et al.* Suicide and unemployment in young people. Analysis of trends in England and Wales, 1921-1995. *Br J Psychiatry* 1999;**175**:263–70. doi:10.1192/bjp.175.3.263
29 Zortea TC, Brenna CTA, Joyce M, *et al.* The Impact of Infectious Disease-Related Public Health Emergencies on Suicide, Suicidal Behavior, and Suicidal Thoughts: A Systematic Review. *Crisis* 2020;:1–14. doi:10.1027/0227-5910/a000753

For peer review only

Figure legends

Figure 1. Annual suicide rate (scaled on the left arithmetic Y-axis) and influenza mortality (scaled on the right logarithmic Y-axis) in Sweden in 1910-1978 per 100 000 inhabitants. *Note*: To ease visualization, influenza death rates are reported on the logarithmic scale, while for the analysis, the logarithmic transformation was performed for suicide rates. Grey areas denote the time periods corresponding to the Spanish influenza pandemic ('S.flu', 1918-1920), the Asian influenza pandemic ('A.flu', 1957-1958), and the Hong Kong influenza pandemic ('HK.flu', 1968-1969) in Sweden.

Figure 2. Sex-specific annual suicide rate (scaled on the left arithmetic Y-axis) and influenza mortality (scaled on the right logarithmic Y-axis) in Sweden in 1910-1978 per 100 000 inhabitants of corresponding sex. *Note*: To ease visualization, influenza death rates are reported on the logarithmic scale, while for the analysis, the logarithmic transformation was performed for suicide rates. Grey areas denote the time periods corresponding to the Spanish influenza pandemic ('S.flu', 1918-1920), the Asian influenza pandemic ('A.flu', 1957-1958), and the Hong Kong influenza pandemic ('HK.flu', 1968-1969) in Sweden.

For peer review only

For peer review only - http://bmjopen.bmj.com/site/about/guidelines.xhtml

Table 1. Short-term and long-term relationship of suicide rates with positive and negative changes in influenza death rates among the whole population, men, and women in 1910-1978 in Sweden

	Whole population		Men		Women	
	Coeff. (95% CI)	p-value	Coeff. (95% CI)	p-value	Coeff. (95% CI)	p-value
**Short-term coefficients**						
Influenza +	0.00002 (-0.00036 to 0.00039)	0.931	0.00004 (-0.00034 to 0.00041)	0.854	-0.00007 (-0.00056 to 0.00041)	0.760
Influenza –	0.00103 (-0.00579 to 0.00785)	0.764	-0.00192 (-0.01005 to 0.00621)	0.638	0.00780 (0.00015 to 0.01544)	0.046
**Long-term coefficients**						
Influenza +	-0.00012	0.998	-0.01314	0.745	0.11254	0.538
Influenza –	0.00211	0.962	0.01443	0.722	-0.10789	0.544
**Model diagnostics**						
Q-test for autocorrelation, χ^2	40.160	0.125	35.260	0.273	31.850	0.424
Heteroscedasticity, χ^2	3.649	0.056	5.439	0.020	1.280	0.258
Normality, χ^2	2.826	0.243	2.237	0.327	2.372	0.305
RESET, F-statistics	4.656	0.006	5.473	0.023	1.013	0.394
CUSUM	Stabl., no str. break	NA	Stabl., no str. break	NA	Stabl., no str. break	NA
CUSUMQ	Stabl.	NA	Stabl.	NA	Stabl.	NA
Adj. R^2	0.257	NA	0.300	NA	0.374	NA
$Wald_{SR}$, F-statistics	0.054	0.817	0.275	0.602	1.062	0.307
$Wald_{LR}$, F-statistics	1.206	0.277	1.176	0.283	0.771	0.384

Note: Signs as "+" and "–" denote the exposure variable being partitioned in positive and negative changes, respectively. Wald test for asymmetry in the short-term ($Wald_{SR}$) and long-term ($Wald_{LR}$) has a null hypothesis of no cointegration. Q-test for autocorrelation reports the results of Portmanteau test for white noise. Heteroscedasticity is measured by Breusch-Pagan/Cook-Weisberg test. Normality is measured by Jarque-Bera test. RESET statistics refers to the regression specification error tests. CUSUM and CUSUMQ refer to the tests of the cumulative sum of recursive residuals and their squares, respectively. "NA" denotes that a certain test parameter was not applicable. Model diagnostics support the validity of the results since, with very few exceptions (RESET statistics for the whole population and men, and heteroscedasticity for men), all diagnostic tests are insignificant indicating that there is no autocorrelation, heteroscedasticity, misspecification and non-normality In

For peer review only - http://bmjopen.bmj.com/site/about/guidelines.xhtml

addition, all CUSUM and CUSUMQ tests all models indicate stability and absence of structural breaks (“Stabl., no str. break” in the results of CUSUM indicates that the model was found stable and the null hypothesis of no structural break was not rejected [same applies to “Stabl.” as an output for CUSUMQ]).

For peer review only

Table 2. Short-term and long-term relationship of suicide rates with positive and negative changes in influenza death rates among the whole population, men, and women in 1918-1956 in Sweden

	Whole population		Men		Women	
	Coeff. (95% CI)	p-value	Coeff. (95% CI)	p-value	Coeff. (95% CI)	p-value
**Short-term coefficients**						
Influenza +	0.00002 (-0.00077 to 0.00082)	0.955	-0.00014 (-0.00102 to 0.00075)	0.755	0.00098 (-0.00068 to 0.00263)	0.237
Influenza –	0.00087 (-0.00643 to 0.00817)	0.809	-0.00132 (-0.01001 to 0.00741)	0.760	0.00434 (-0.00595 to 0.01463)	0.394
**Long-term coefficients**						
Influenza +	0.00011	0.994	-0.00490	0.802	0.01403	0.521
Influenza –	0.00103	0.936	0.00548	0.775	-0.01098	0.600
**Model diagnostics**						
Q-test for autocorrelation, χ^2	14.130	0.658	9.674	0.917	9.614	0.919
Heteroscedasticity, χ^2	0.939	0.333	0.768	0.381	5.042	0.025
Normality, χ^2	0.420	0.811	0.132	0.936	2.383	0.304
RESET, F-statistics	0.413	0.745	0.764	0.524	0.505	0.683
CUSUM	Stabl., no str. break	NA	Stabl., no str. break	NA	Stabl., no str. break	NA
CUSUMQ	Stabl.	NA	Stabl.	NA	Stabl.	NA
Adj. R^2	0.309	NA	0.345	NA	0.432	NA
$Wald_{SR}$, F-statistics	0.056	0.815	0.155	0.697	0.183	0.673
$Wald_{LR}$, F-statistics	1.555	0.222	0.217	0.645	4.479	0.044

Note: Signs as "+" and "–" denote the exposure variable being partitioned in positive and negative changes, respectively. Wald test for asymmetry in the short-term ($Wald_{SR}$) and long-term ($Wald_{LR}$) has a null hypothesis of no cointegration. Q-test for autocorrelation reports the results of Portmanteau test for white noise. Heteroscedasticity is measured by Breusch-Pagan/Cook-Weisberg test. Normality is measured by Jarque-Bera test. RESET statistics refers to the regression specification error tests. CUSUM and CUSUMQ refer to the tests of the cumulative sum of recursive residuals and their squares, respectively. "NA" denotes that a certain test parameter was not applicable. Model diagnostics support the validity of the results since, with one exception (heteroscedasticity for women), all diagnostic tests are insignificant indicating that ere is no autocorrelation, heteroscedasticity, misspecification and non-normality In addition, all CUSUM and CUSUMQ tests all models indicate

For peer review only - http://bmjopen.bmj.com/site/about/guidelines.xhtml

stability and absence of structural breaks ("Stabl., no str. break" in the results of CUSUM indicates that the model was found stable and the null hypothesis of no structural break was not rejected [same applies to "Stabl." as an output for CUSUMQ]).

For peer review only

Table 3. Short-term and long-term relationship of suicide rates with positive and negative changes in influenza death rates among the whole population, men, and women in 1957-1978 in Sweden

	Whole population		Men		Women	
	**Coeff. (95% CI)**	**p-value**	**Coeff. (95% CI)**	**p-value**	**Coeff. (95% CI)**	**p-value**
**Short-term coefficients**						
Influenza +	-0.00964 (-0.02252 to 0.00324)	0.883	-0.00876 (-0.02738 to 0.00985)	0.328	-0.00976 (-0.02033 to 0.00081)	0.068
Influenza –	0.00194 (-0.02614 to 0.03003)	0.130	-0.01531 (-0.04658 to 0.01596)	0.309	0.02155 (-0.00343 to 0.04653)	0.085
**Long-term coefficients**						
Influenza +	0.01255	0.618	-0.02659	0.541	0.04091	0.064
Influenza –	-0.01033	0.684	0.024538	0.582	-0.02870	0.184
**Model diagnostics**						
Q-test for autocorrelation, χ^2	5.662	0.773	3.790	0.925	5.243	0.813
Heteroscedasticity, χ^2	0.102	0.750	0.155	0.694	0.079	0.778
Normality, χ^2	1.569	0.456	1.627	0.443	1.237	0.537
RESET, F-statistics	5.839	0.014	3.348	0.064	1.089	0.398
CUSUM	Stabl., no str. break	NA	Stabl., no str. break	NA	Stabl., no str. break	NA
CUSUMQ	Stabl.	NA	Stabl.	NA	Stabl.	NA
Adj. R^2	0.246	NA	0.337	NA	0.371	NA
$Wald_{SR}$, F-statistics	2.903	0.112	0.536	0.477	7.132	0.019
$Wald_{LR}$, F-statistics	0.922	0.354	0.297	0.595	26.74	<0.001

Note: Signs as "+" and "–" denote the exposure variable being partitioned in positive and negative changes, respectively. Wald test for asymmetry in the short-term ($Wald_{SR}$) and long-term ($Wald_{LR}$) has a null hypothesis of no cointegration. Q-test for autocorrelation reports the results of Portmanteau test for white noise. Heteroscedasticity is measured by Breusch-Pagan/Cook-Weisberg test. Normality is measured by Jarque-Bera test. RESET statistics refers to the regression specification error tests. CUSUM and CUSUMQ refer to the tests of the cumulative sum of recursive residuals and their squares, respectively. "NA" denotes that a certain test parameter was not applicable. Model diagnostics support the validity of the results since, with one exception (RESET statistics for the whole population), all diagnostic tests are insignificant indicating that there is no autocorrelation, heteroscedasticity, misspecification and non-normality In addition, all CUSUM and CUSUMQ tests all

For peer review only - http://bmjopen.bmj.com/site/about/guidelines.xhtml

models indicate stability and absence of structural breaks ("Stabl., no str. break" in the results of CUSUM indicates that the model was found stable and the null hypothesis of no structural break was not rejected [same applies to "Stabl." as an output for CUSUMQ]).

For peer review only

For peer review only - http://bmjopen.bmj.com/site/about/guidelines.xhtml

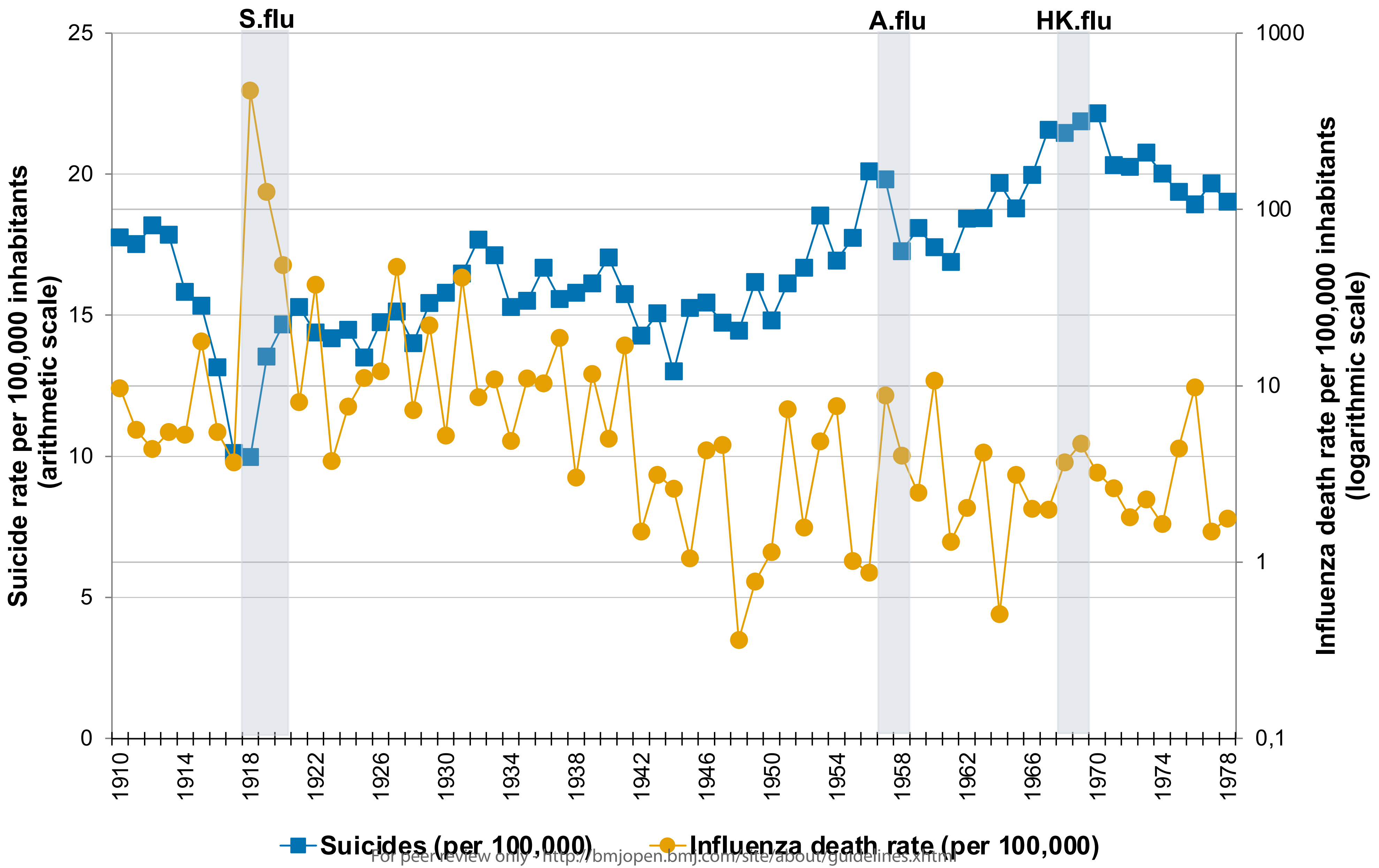

For peer review only - http://bmjopen.bmj.com/site/about/guidelines.xhtml

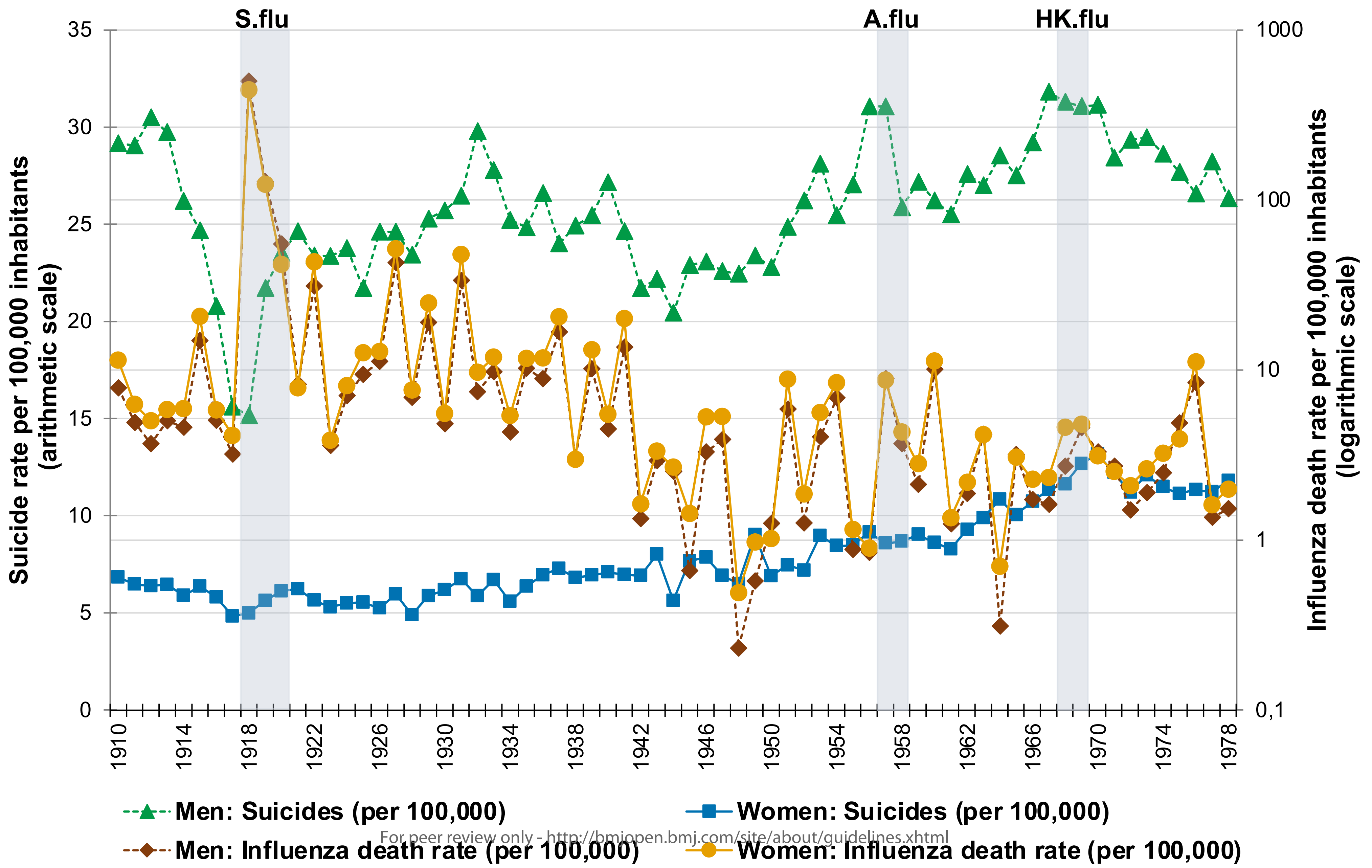
S.flu
A.flu
HK.flu
35
30
25
20
15
10
5
0
1000
100
10
1
0,1
Suicide rate per 100,000 inhabitants
(arithmetic scale)
Influenza death rate per 100,000 inhabitants
(logarithmic scale)
1910
1914
1918
1922
1926
1930
1934
1938
1942
1946
1950
1954
1958
1962
1966
1970
1974
1978
Men: Suicides (per 100,000)
Women: Suicides (per 100,000)
Men: Influenza death rate (per 100,000)
Women: Influenza death rate (per 100,000)

Supplementary material

eTable 1. Original data on the number of deaths by influenza and suicide, and population size for total population, men, and women in 1910-1978, retrieved from the Statistical Yearbook of Sweden from 1910-1978

Year	Total population, N	Men, N	Women, N	Deaths by influenza in total population, N	Deaths by influenza among men, N	Deaths by influenza among women, N	Deaths by suicide in total population, N	Deaths by suicide among men, N	Deaths by suicide among women, N
1910	5522403	2698729	2823674	534	212	322	980	787	193
1911	5561799	2718638	2843161	312	134	178	974	790	184
1912	5604192	2740737	2863455	245	101	144	1019	836	183
1913	5638583	2756946	2881637	307	139	168	1006	820	186
1914	5679607	2777447	2902160	300	128	172	899	728	171
1915	5712740	2794552	2918188	1018	417	601	876	690	186
1916	5757566	2817950	2939616	314	143	171	757	586	171
1917	5800847	2841554	2959293	213	91	122	587	444	143
1918	5813850	2849205	2964645	27379	14245	13134	580	432	148
1919	5847037	2868395	2978642	7341	3667	3674	791	623	168
1920	5904489	2898256	3006233	2853	1596	1257	866	682	184
1921	5954316	2925988	3028328	479	241	238	910	721	189
1922	5987520	2944031	3043489	2235	918	1317	861	689	172
1923	6005759	2948508	3057251	224	106	118	851	689	162
1924	6036118	2964230	3071888	458	210	248	874	705	169
1925	6053562	2972554	3081008	669	280	389	817	646	171
1926	6074368	2982625	3091743	731	335	396	896	734	162
1927	6087923	2990205	3097718	2875	1277	1598	921	736	185
1928	6105190	2999562	3105628	444	207	237	855	703	152
1929	6120080	3007946	3112134	1345	573	772	944	761	183
1930	6142191	3020848	3121343	319	146	173	970	777	193
1931	6162446	3037064	3125382	2518	1025	1493	1015	804	211
1932	6190364	3053528	3136836	531	228	303	1094	910	184
1933	6211566	3066888	3144678	673	300	373	1063	852	211
1934	6233090	3079690	3153400	303	133	170	953	777	176
1935	6250506	3090451	3160055	685	316	369	969	768	201
1936	6266888	3100534	3166354	646	275	371	1045	825	220
1937	6284722	3111256	3173466	1173	522	651	978	747	231
1938	6310214	3125000	3185214	190	95	95	996	779	217
1939	6341303	3142356	3198947	740	319	421	1022	800	222
1940	6371432	3160128	3211304	318	142	176	1086	858	228
1941	6406474	3180535	3225939	1082	434	648	1009	784	225
1942	6458200	3207756	3250444	96	43	53	922	697	225
1943	6522827	3240631	3282196	204	95	109	982	719	263
1944	6597348	3279723	3317625	172	83	89	858	671	187
1945	6673749	3321502	3352247	70	22	48	1018	761	257
1946	6763685	3366694	3396991	291	111	180	1044	777	267
1947	6842046	3407577	3434469	316	133	183	1008	770	238

For peer review only - http://bmjopen.bmj.com/site/about/guidelines.xhtml

Supplementary material

1948	6924888	3448122	3476766	25	8	17	1000	774	226
1949	6986181	3479079	3507102	54	20	34	1130	814	316
1950	7041829	3506442	3535387	80	44	36	1043	799	244
1951	7098740	3535736	3563004	522	208	314	1145	879	266
1952	7150606	3562475	3588131	112	45	67	1192	934	258
1953	7192316	3583598	3608718	347	145	202	1332	1008	324
1954	7234664	3605013	3629651	553	248	305	1225	918	307
1955	7290112	3633983	3656129	74	32	42	1293	983	310
1956	7338991	3659917	3679074	64	31	33	1474	1137	337
1957	7388611	3685654	3702957	649	327	322	1463	1145	318
1958	7429675	3706039	3723636	297	137	160	1282	959	323
1959	7462823	3722867	3739956	184	79	105	1350	1012	338
1960	7497967	3740119	3757848	801	378	423	1305	981	324
1961	7542028	3763040	3778988	98	47	51	1273	960	313
1962	7581148	3782252	3798896	154	71	83	1396	1043	353
1963	7627507	3805699	3821808	319	160	159	1406	1028	378
1964	7695200	3840897	3854303	39	12	27	1514	1096	418
1965	7772506	3882473	3890033	243	124	119	1459	1068	391
1966	7843088	3919170	3923918	157	68	89	1566	1145	421
1967	7892774	3942223	3950551	156	64	92	1702	1254	448
1968	7934996	3961414	3973582	291	108	183	1702	1240	462

For peer review only

For peer review only - http://bmjopen.bmj.com/site/about/guidelines.xhtml

Supplementary material

Supplementary material. Model diagnostics

We carried out several types of model diagnostics, including Portmanteau test for white noise to check autocorrelation in residuals, Breusch-Pagan/Cook-Weisberg test for heteroscedasticity, Jarque-Bera test on normality, and the regression specification error tests that indicates whether there is a misspecification in the model. We also performed tests for stability of the models by using the cumulative sum of recursive residuals and their squares to test whether there is a structural break due to changes in regression coefficients over time.

Supplementary material. STATA codes for the non-linear autoregressive distributed lag (NARDL) modelling of association between between influenza death rates and suicide rates

```
clear
use "Z:\Influenza_deaths_suicides\ALL19101978.dta"


///// 1. DATA MANAGEMENT: generate and label variables to be used in the analyses


/// Labels for original variables (retrieved from the Statistical Yearbooks 1910-1978)

 lab var year "Years 1910-1978"

 lab var number_population_all "Total number of inhabitants (population size) in Sweden in corresponding year"
 lab var number_population_men "Total number of male population in Sweden in corresponding year"
 lab var number_population_women "Total number of female population in Sweden in corresponding year"

 lab var number_influenza_all "Number of deaths from influenza in total population in corresponding year"
 lab var number_influenza_men "Number of deaths from influenza among men in corresponding year"
 lab var number_influenza_women "Number of deaths from influenza among women in corresponding year"

 lab var number_suicide_all "Number of suicides in total population in corresponding year"
 lab var number_suicide_men "Number of suicides among men in corresponding year"
 lab var number_suicide_women "Number of suicides among women in corresponding year"



/// Calculation of annual influenza death rates and suicide rates per 100,000 for total population, men, and
women

 gen influenza_rates_all = (number_influenza_all/number_population_all)*100000
 gen influenza_rates_men = (number_influenza_men/number_population_men)*100000
 gen influenza_rates_women = (number_influenza_women/number_population_women)*100000

 gen suicide_rates_all = (number_suicide_all/number_population_all)*100000
 gen suicide_rates_men = (number_suicide_men/number_population_men)*100000
 gen suicide_rates_women = (number_suicide_women/number_population_women)*100000

 lab var influenza_rates_all "Influenza death rates for total population per 100,000, annual"
 lab var influenza_rates_men "Influenza death rates among men per 100,000, annual"
 lab var influenza_rates_women "Influenza death rates among women per 100,000, annual"

 lab var suicide_rates_all "Suicide rates for total population per 100,000, annual"
 lab var suicide_rates_men "Suicide rates among men per 100,000, annual"
 lab var suicide_rates_women "Suicide rates among women per 100,000, annual"



/// Logarithmic transformation for suicide rates for total population, men, and women
```


For peer review only - http://bmjopen.bmj.com/site/about/guidelines.xhtml

Supplementary material

```
gen ln_suicide_rates_all = ln(suicide_rates_all)
gen ln_suicide_rates_men = ln(suicide_rates_men)
gen ln_suicide_rates_women = ln(suicide_rates_women)

lab var ln_suicide_rates_all "Log-transformed suicide rates for total population per 100,000, annual"
lab var ln_suicide_rates_men "Log-transformed suicide rates among men per 100,000, annual"
lab var ln_suicide_rates_women "Log-transformed suicide rates among women per 100,000, annual"



/// Variables for changes in death registration in Sweden 1910-1978 (as dummy variables)

// based on the Bertillon criteria (prior to 1931)
gen registration_19101930=0
replace registration_19101930=1 if year<=1930
lab var registration_19101930 "Dummy variable for death registration in 1910-1930 (Bertillion)"

// introduced in cooperation with other Nordic countries (1931-1950)
gen registration_19311950=0
replace registration_19311950=1 if year>=1931 & year<=1950
lab var registration_19311950 "Dummy variable for death registration in 1931-1950 (new registration)"

// ICD-6 (1951-1957)
gen registration_19511957=0
replace registration_19511957=1 if year>=1951 & year<=1957
lab var registration_19511957 "Dummy variable for death registration in 1951-1957 (ICD-6)"

// ICD-7 (1958-1968)
gen registration_19581968=0
replace registration_19581968=1 if year>=1958 & year<=1968
lab var registration_19581968 "Dummy variable for death registration in 1958-1968 (ICD-7)"

// ICD-8 (1969-1978)
gen registration_1969after=0
replace registration_1969after=1 if year>=1969
lab var registration_1969after "Dummy variable for death registration in 1969 and after (ICD-8)"
//////////////////////////////////////////////////////////////////////////////////



///// 2. CHECKING VARIABLES' PROPERTIES AND TESTING THE CONDITIONS FOR MODELLING


/// Declare data to be time-series data
tsset year, yearly


/// Obtain optimal lags for each variable (for (i) augmentation in ADF and KPSS tests, and (ii) for p and q parameters in NARDL).
/// Lags obtained for influenza death rates and logarithmically-transformed suicide rates for total population, men, and women, and for time periods 1910-1978, 1918-1956, and 1957-1978
/// If AIC, HQIC, and SBIC information criteria indicated different lag orders, SBIC was used to select optimal lags

varsoc influenza_rates_all
varsoc influenza_rates_all if tin(1918, 1956)
varsoc influenza_rates_all if tin(1957, )

varsoc influenza_rates_men
```


For peer review only

Supplementary material

```
varsoc influenza_rates_men if tin(1918, 1956)
varsoc influenza_rates_men if tin(1957, )

varsoc influenza_rates_women
varsoc influenza_rates_women if tin(1918, 1956)
varsoc influenza_rates_women if tin(1957, )

varsoc ln_suicide_rates_all
varsoc ln_suicide_rates_all if tin(1918, 1956)
varsoc ln_suicide_rates_all if tin(1957, )

varsoc ln_suicide_rates_men
varsoc ln_suicide_rates_men if tin(1918, 1956)
varsoc ln_suicide_rates_men if tin(1957, )

varsoc ln_suicide_rates_women
varsoc ln_suicide_rates_women if tin(1918, 1956)
varsoc ln_suicide_rates_women if tin(1957, )


/// Tests for stationarity: ADF and KPSS for influenza death rates and logarithmically-transformed suicide rates variables for total population, men, and women
/// Augmentation by at least one lag was used (for suicide rates in women - by two lags according to SBIC in varsoc)

dfuller influenza_rates_all, lag(1)
dfuller influenza_rates_men, lag(1)
dfuller influenza_rates_women, lag(1)

dfuller ln_suicide_rates_all, lag(1)
dfuller ln_suicide_rates_men, lag(1)
dfuller ln_suicide_rates_women, lag(1)
dfuller ln_suicide_rates_women, lag(2)

dfuller D.influenza_rates_all, lag(1)
dfuller D.influenza_rates_men, lag(1)
dfuller D.influenza_rates_women, lag(1)

dfuller D.ln_suicide_rates_all, lag(1)
dfuller D.ln_suicide_rates_men, lag(1)
dfuller D.ln_suicide_rates_women, lag(1)
dfuller D.ln_suicide_rates_women, lag(2)

kpss influenza_rates_all, maxlag(1) notrend
kpss influenza_rates_men, maxlag(1) notrend
kpss influenza_rates_women, maxlag(1) notrend

kpss ln_suicide_rates_all, maxlag(1) notrend
kpss ln_suicide_rates_men, maxlag(1) notrend
kpss ln_suicide_rates_women, maxlag(2) notrend

kpss D.influenza_rates_all, maxlag(1) notrend
kpss D.influenza_rates_men, maxlag(1) notrend
kpss D.influenza_rates_women, maxlag(1) notrend

kpss D.ln_suicide_rates_all, maxlag(1) notrend
kpss D.ln_suicide_rates_men, maxlag(1) notrend
kpss D.ln_suicide_rates_women, maxlag(2) notrend
```


For peer review only

Supplementary material

///// 3. ESTIMATION OF NON-LINEAR AUTOREGRESSIVE DISTRIBUTED LAG (NARDL) MODELS: for total population, men, and women, and for time periods 1910-1978, 1918-1956, and 1957-1978
/// The dependent and independent variables are indicated in levels.
/// The covariates (i.e., changes in death registration system 1910-1978) are included with the deterministic option, but these were only kept in the models if statistically significant.
/// The optimal number of lags for dependent and independent variables (p and q parameters, respectively).
/// Since p and q parameters refer to levels, one additional lag is added to p and q to get an optimal lag length in differences (p and q must be at least 2).
/// The model provides an output for a long-term cointegration bounds test and diagnostic tests.

```
nardl ln_suicide_rates_all influenza_rates_all, p(2) q(2) h(69) plot bootstrap(100) level(95) residuals /* used as a final model */
nardl ln_suicide_rates_all influenza_rates_all, p(2) q(2) deterministic(registration_19101930 registration_19311950 registration_19511957 registration_19581968 registration_1969after) h(69) plot bootstrap(100) level(95) residuals

nardl ln_suicide_rates_men influenza_rates_men, p(2) q(2) h(69) plot bootstrap(100) level(95) residuals /* used as a final model */
nardl ln_suicide_rates_men influenza_rates_men, p(2) q(2) deterministic(registration_19101930 registration_19311950 registration_19511957 registration_19581968 registration_1969after) h(69) plot bootstrap(100) level(95) residuals

nardl ln_suicide_rates_women influenza_rates_women, p(3) q(2) h(69) plot bootstrap(100) level(95) residuals /* used as a final model */
nardl ln_suicide_rates_women influenza_rates_women, p(3) q(2) deterministic(registration_19101930 registration_19311950 registration_19511957 registration_19581968 registration_1969after) h(69) plot bootstrap(100) level(95) residuals

nardl ln_suicide_rates_all influenza_rates_all if tin(1918,1956), p(2) q(2) h(39) plot bootstrap(50) level(95) residuals
nardl ln_suicide_rates_men influenza_rates_men if tin(1918,1956), p(2) q(2) h(39) plot bootstrap(50) level(95) residuals
nardl ln_suicide_rates_women influenza_rates_women if tin(1918,1956), p(5) q(2) h(39) plot bootstrap(50) level(95) residuals

nardl ln_suicide_rates_all influenza_rates_all if tin(1957, ), p(2) q(2) h(22) plot bootstrap(50) level(95) residuals
nardl ln_suicide_rates_men influenza_rates_men if tin(1957, ), p(2) q(2) h(22) plot bootstrap(50) level(95) residuals
nardl ln_suicide_rates_women influenza_rates_women if tin(1957, ), p(2) q(2) h(22) plot bootstrap(50) level(95) residuals
```

/* REFERENCES

For varsoc and dfuller:
StataCorp. Stata 15 Base Reference Manual. College Station, TX: Stata Press; 2017

For kpss:
Author: Christopher F Baum, Boston College, USA baum@@bc.edu
Kwiatkowski, D., Phillips, P.C.B., Schmidt, P. and Y. Shin. Testing the null hypothesis of stationarity against the alternative of a unit root: How sure are we that economic time series have a unit root? Journal of Econometrics, 54, 1992, 159-178.
Lee, D. and P. Schmidt. On the power of the KPSS test of stationarity against fractionally-integrated alternatives. Journal of Econometrics, 73, 1996, 285-302.
Schwert, G.W. Tests for Unit Roots: A Monte Carlo Investigation. Journal of Business and Economic Statistics, 7, 1989, 147-160.

For nardl:

For peer review only - http://bmjopen.bmj.com/site/about/guidelines.xhtml

Supplementary material

Author: Stata implementation by Marco Sunder (sunder@wifa.uni-leipzig.de). This version was created on 26jan2012.
Shin, Y., Yu, B., Greenwood-Nimmo, M. (2011): Modelling asymmetric cointegration and dynamic multipliers in a nonlinear ARDL framework. Working paper (version of November 2011), http://ssrn.com/abstract=1807745. */

end of do-file

For peer review only

Supplementary material

eTable 2. Unit root tests at the level and first difference by Augmented Dickey-Fuller (ADF) and Kwiatkowski-Phillips-Schmidt-Shin (KPSS) test statistics

	ADF at level		KPSS at level		Results for stationarity at the level	ADF at first difference		KPSS at first difference		Results for stationarity at the 1st difference	Order of integration
	Test statistic	p-value	Test statistic	p-value[1]		Test statistic	p-value	Test statistic	p-value[1]		
**Suicide rate (log-transformed)**											
All	-2.181	0.213	1.980	<0.001	nonstationary	-6.640	<0.001	0.068	>0.1	stationary	1
Males	-3.230	0.018	0.964	<0.001	nonstationary	-6.512	<0.001	0.053	>0.1	stationary	1
Females	-0.517	0.889	3.130	<0.001	nonstationary	-8.692	<0.001	0.104	>0.1	stationary	1
**Influenza death rate**											
All	-4.895	<0.001	0.461	>0.1	stationary	*-8.727*	*<0.001*	*0.016*	>0.1	*stationary*	0
Males	-4.856	<0.001	0.431	>0.1	stationary	*-8.642*	*<0.001*	*0.016*	>0.1	*stationary*	0
Females	-4.940	<0.001	0.491	>0.1	stationary	*-8.824*	*<0.001*	*0.015*	>0.1	*stationary*	0

Note: For the ADF test, the null hypothesis implies that the variable contains a unit root (the alternate hypothesis is that the variable is stationary), whereas for the KPSS test the null hypothesis implies that the variable is stationary (the alternate hypothesis is that there is a unit root). The results for ADF and KPSS tests for stationarity at first difference for influenza death rates are reported as explanatory (written in Italics) since the stationarity at the level has already been established (i.e., integrated of the order zero).

[1] KPSS test results do not indicate the exact p-value, but report the level of significance at which the null hypothesis is rejected (1%, 2.5%, 5%, or 10% significance level).

Abbreviations: ADF, Augmented Dickey–Fuller unit-root test; KPSS, Kwiatkowski-Phillips-Schmidt-Shin test for stationarity

For peer review only

Supplementary material

eTable 3. **Full specification for non-linear autoregressive distributed lag models used for the analysis of short-term and long-term relationship of suicide rates with positive and negative changes in influenza death rates among the whole population, men, and women in 1910-1978 in Sweden**

	Whole population		Men		Women	
	Coefficient (95% CI)	p-value	Coefficient (95% CI)	p-value	Coefficient (95% CI)	p-value
LnSuicide, lag 1	-0.08466 (-0.23940 to 0.07009)	0.278	-0.11457 (-0.30588 to 0.07674)	0.235	-0.04517 (-0.15832 to 0.06799)	0.427
Influenza +, lag 1	-0.00001 (-0.00761 to 0.00759)	0.998	-0.00150 (-0.01032 to 0.00732)	0.734	0.00508 (-0.00366 to 0.01382)	0.249
Influenza –, lag 1	-0.00018 (-0.00775 to 0.00739)	0.962	-0.00165 (-0.01044 to 0.00714)	0.708	0.00487 (-0.00383 to 0.01358)	0.267
ΔLnSuicide, lag 1	-0.14610 (-0.43113 to 0.13893)	0.309	0.029465 (-0.27204 to 0.33097)	0.846	-0.65184 (-0.91785 to -0.38582)	<0.001
ΔLnSuicide, lag 2	NA	NA	NA	NA	-0.30807 (-0.55722 to -0.05892)	0.016
ΔInfluenza +	0.00002 (-0.00036 to 0.00039)	0.931	0.00004 (-0.00034 to 0.00041)	0.854	-0.00007 (-0.00056 to 0.00041)	0.760
ΔInfluenza +, lag 1	0.00153 (-0.00285 to 0.00590)	0.488	0.00085 (-0.00347 to 0.00518)	0.694	0.00093 (-0.00482 to 0.00668)	0.747
ΔInfluenza –	0.00103 (-0.00579 to 0.00785)	0.764	-0.00192 (-0.01005 to 0.00621)	0.638	0.00780 (0.00015 to 0.01544)	0.046
ΔInfluenza –, lag 1	-0.00070 (-0.00236 to 0.00096)	0.401	-0.00038 (-0.00212 to 0.00136)	0.663	-0.000075 (-0.00289 to 0.00139)	0.488
**Long-term effect**						
Influenza +	-0.00012	0.998	-0.01314	0.745	0.11254	0.538
Influenza –	0.00211	0.962	0.01443	0.722	-0.10789	0.544
**Model diagnostics**						

For peer review only - http://bmjopen.bmj.com/site/about/guidelines.xhtml

Supplementary material

Q-test for autocorrelation, χ^2	40.160	0.125	35.260	0.273	31.850	0.424
Heteroscedasticity, χ^2	3.649	0.056	5.439	0.020	1.280	0.258
Normality, χ^2	2.826	0.243	2.237	0.327	2.372	0.305
RESET, F-statistics	4.656	0.006	5.473	0.023	1.013	0.394
CUSUM	Stable, no structural break	NA	Stable, no structural break	NA	Stable, no structural break	NA
CUSUMQ	Stable	NA	Stable	NA	Stable	NA
Adj. R^2	0.257	NA	0.300	NA	0.374	NA
$Wald_{SR}$, F-statistics	0.054	0.817	0.275	0.602	1.062	0.307
$Wald_{LR}$, F-statistics	1.206	0.277	1.176	0.283	0.771	0.384
**Cointegration test statistics**						
t_BDM	-1.095	NA	-1.199	NA	-0.799	NA
F_PSS	3.789	NA	3.735	NA	2.530	NA
**Critical values for F_PSS**						
5% critical values; I(0), I(1)	k=1: 5.055, 5.915<br>k=2: 3.947, 5.020	NA	k=1: 5.055, 5.915<br>k=2: 3.947, 5.020	NA	k=1: 5.055, 5.915<br>k=2: 3.947, 5.020	NA

Note: F_PSS denotes F-statistics for Pesaran/Shin/Smith bounds test with null hypothesis of no cointegration. Critical values are retrieved from Narayan PK (2005) for a sample size of n=70.[1] The null hypothesis of no cointegration is supported if F_PSS statistics is lower than 5% I(0) critical value; the null hypothesis is rejected if F_PSS statistics is greater than 5% I(1) critical value. We applied a conservative approach to considering the number of exposure variables (k), as suggested by Shin Y, et al (2014) [2] (since influenza death rates represent one exposure (k=1), while the analysis actually partitions the

For peer review only - http://bmjopen.bmj.com/site/about/guidelines.xhtml

Supplementary material

exposure to positive and negative changes (k=2)). In a given table, the results of F_PSS for the whole population, men, and women are lower than the reported 5% I(0) critical values that accepts a null hypothesis of no cointegration.

"LnSuicide" indicates that suicide rates were logarithmically-transformed. "Δ" signifies the series is differenced. Signs as "+" and "–" denote the exposure variable being partitioned in positive and negative changes, respectively. "NA" denotes that a certain test or test parameter is not applicable.

Number of lags used for each variable in the model are noted by "lag#". To select the optimal number of lags to be used for choosing p and q parameters for the NARDL (i.e., numbers of lags for dependent and independent variables, respectively), we applied a varsoc command in STATA using the minimal values of Akaike Information Criterion, Schwarz's Bayesian information criterion (SBIC), and the Hannan and Quinn information criterion information criteria. If information criteria indicated different lag orders, SBIC was used to select optimal lags. Thus, for the analysis of the whole population and men, both exposure and outcome time series were lagged once, while for analysis among women to outcome time series were lagged twice and exposure time series were lagged once.

Wald test for asymmetry in a short-term ($Wald_{SR}$) and long-term ($Wald_{LR}$) has a null hypothesis of no cointegration. Q-test for autocorrelation reports the results of Portmanteau test for white noise. Heteroscedasticity is measured by Breusch-Pagan/Cook-Weisberg test. Normality is measured by Jarque-Bera test. RESET statistics refers to the regression specification error tests. CUSUM and CUSUMQ refer to the tests of the cumulative sum of recursive residuals and their squares, respectively. "Stable, no structural break" in the results of CUSUM indicates that the model was found stable and the null hypothesis of no structural break was not rejected (same applies to "Stable" as an output for CUSUMQ).

For peer review only - http://bmjopen.bmj.com/site/about/guidelines.xhtml

Supplementary material

eTable 4. Full specification for non-linear autoregressive distributed lag models used for the analysis of short-term and long-term relationship of suicide rates with positive and negative changes in influenza death rates among the whole population, men, and women in 1918-1956 in Sweden

	Whole population		Men		Women	
	Coefficient (95% CI)	p-value	Coefficient (95% CI)	p-value	Coefficient (95% CI)	p-value
LnSuicide, lag 1	-0.32229 (-0.73472 to 0.09015)	0.121	-0.26417 (-0.60333 to 0.07492)	0.122	-0.36067 (-0.97384 to 0.25250)	0.238
Influenza +, lag 1	0.00003 (-0.00851 to 0.00858)	0.994	-0.00129 (-0.011384 to 0.00879)	0.795	0.00506 (-0.00682 to 0.01695)	0.390
Influenza –, lag 1	-0.00033 (-0.00869 to 0.00803)	0.936	-0.00145 (-0.01129 to 0.00840)	0.766	0.00396 (-0.00801 to 0.01593)	0.503
ΔLnSuicide, lag 1	-0.19387 (-0.59955 to 0.21181)	0.337	-0.03045 (-0.44243 to 0.38153)	0.881	-0.58189 (-1.19784 to 0.03406)	0.063
ΔLnSuicide, lag 2	NA	NA	NA	NA	-0.36148 (-0.91643 to 0.19346)	0.193
ΔLnSuicide, lag 3	NA	NA	NA	NA	-0.11520 (-0.61658 to 0.38617)	0.641
ΔLnSuicide, lag 4	NA	NA	NA	NA	0.11306 (-0.26409 to 0.49021)	0.544
ΔInfluenza +	0.00002 (-0.00077 to 0.00082)	0.955	-0.00014 (-0.00102 to 0.00075)	0.755	0.00098 (-0.00068 to 0.00263)	0.237
ΔInfluenza +, lag 1	0.00140 (-0.00347 to 0.00627)	0.561	0.00095 (-0.00389 to 0.00580)	0.691	-0.00063 (-0.00821 to 0.00696)	0.866
ΔInfluenza –	0.00087 (-0.00643 to 0.00817)	0.809	-0.00132 (-0.01001 to 0.00741)	0.760	0.00434 (-0.00595 to 0.01463)	0.394
ΔInfluenza –, lag 1	-0.00076 (-0.00256 to 0.00104)	0.396	-0.00044 (-0.00233 to 0.00145)	0.637	-0.00052 (-0.00328 to 0.00224)	0.704
**Long-term effect**						
Influenza +	0.00011	0.994	-0.00490	0.802	0.01403	0.521

For peer review only - http://bmjopen.bmj.com/site/about/guidelines.xhtml

Supplementary material

Influenza –	0.00103	0.936	0.00548	0.775	-0.01098	0.600
**Model diagnostics**						
Q-test for autocorrelation, χ^2	14.130	0.658	9.674	0.917	9.614	0.919
Heteroscedasticity, χ^2	0.939	0.333	0.768	0.381	5.042	0.025
Normality, χ^2	0.420	0.811	0.132	0.936	2.383	0.304
RESET, F-statistics	0.413	0.745	0.764	0.524	0.505	0.683
CUSUM	Stable, no structural break	NA	Stable, no structural break	NA	Stable, no structural break	NA
CUSUMQ	Stable	NA	Stable	NA	Stable	NA
Adj. R^2	0.309	NA	0.345	NA	0.432	NA
$Wald_{SR}$, F-statistics	0.056	0.815	0.155	0.697	0.183	0.673
$Wald_{LR}$, F-statistics	1.555	0.222	0.217	0.645	4.479	0.044
**Cointegration test statistics**						
t_BDM	-1.596	NA	-1.591	NA	-1.207	NA
F_PSS	1.009	NA	1.019	NA	1.328	NA
**Critical values for F_PSS**						
5% critical values; I(0) to I(1)	k=1: 5.260, 6.160<br>k=2: 4.133, 5.260	NA	k=1: 5.260, 6.160<br>k=2: 4.133, 5.260	NA	k=1: 5.260, 6.160<br>k=2: 4.133, 5.260	NA

For peer review only

Supplementary material

Note: F_PSS denotes F-statistics for Pesaran/Shin/Smith bounds test with null hypothesis of no cointegration. Critical values are retrieved from Narayan PK (2005) for a sample size of n=40 [1]. The null hypothesis of no cointegration is supported if F_PSS statistics is lower than 5% I(0) critical value; the null hypothesis is rejected if F_PSS statistics is greater than 5% I(1) critical value. We applied a conservative approach to considering the number of exposure variables (k), as suggested by Shin Y, et al (2014) [2] (since influenza death rates represent one exposure (k=1), while the analysis actually partitions the exposure to positive and negative changes (k=2)). In a given table, the results of F_PSS for the whole population, men, and women are lower than the reported 5% I(0) critical values that accepts a null hypothesis of no cointegration.

"LnSuicide" indicates that suicide rates were logarithmically-transformed. "Δ" signifies the series is differenced. Signs as "+" and "–" denote the exposure variable being partitioned in positive and negative changes, respectively. "NA" denotes that a certain test or test parameter is not applicable.

Number of lags used for each variable in the model are noted by "lag#". To select the optimal number of lags to be used for choosing p and q parameters for the NARDL (i.e., numbers of lags for dependent and independent variables, respectively), we applied a varsoc command in STATA using the minimal values of Akaike Information Criterion, Schwarz's Bayesian information criterion (SBIC), and the Hannan and Quinn information criterion information criteria. If information criteria indicated different lag orders, SBIC was used to select optimal lags. Thus, for the analysis of the whole population and men, both exposure and outcome time series were lagged once, while for analysis among women, outcome time series were lagged four times and exposure time series were lagged once.

Wald test for asymmetry in a short-term ($Wald_{SR}$) and long-term ($Wald_{LR}$) has a null hypothesis of no cointegration. Q-test for autocorrelation reports the results of Portmanteau test for white noise. Heteroscedasticity is measured by Breusch-Pagan/Cook-Weisberg test. Normality is measured by Jarque-Bera test. RESET statistics refers to the regression specification error tests. CUSUM and CUSUMQ refer to the tests of the cumulative sum of recursive residuals and their squares, respectively. "Stable, no structural break" in the results of CUSUM indicates that the model was found stable and the null hypothesis of no structural break was not rejected (same applies to "Stable" as an output for CUSUMQ).

For peer review only

Supplementary material

eTable 5. Full specification for non-linear autoregressive distributed lag models used for the analysis of short-term and long-term relationship of suicide rates with positive and negative changes in influenza death rates among the whole population, men, and women in 1957-1978 in Sweden

	Whole population		Men		Women	
	Coefficient (95% CI)	p-value	Coefficient (95% CI)	p-value	Coefficient (95% CI)	p-value
LnSuicide, lag 1	-0.64009 (-1.15104 to -0.12914)	0.018	-0.52238 (-1.11901 to 0.07425)	0.081	-0.66040 (-1.04522 to -0.27558)	0.003
Influenza +, lag 1	0.008034 (-0.02823 to 0.04429)	0.640	-0.01385 (-0.05324 to 0.02553)	0.461	0.02702 (-0.00364 to 0.05768)	0.079
Influenza –, lag 1	0.00661 (-0.02823 to 0.04429)	0.699	-0.01281 (-0.054045 to 0.02841)	0.514	0.01895 (-0.01066 to 0.04857)	0.190
ΔLnSuicide, lag 1	0.14293 (-0.41621 to 0.70206)	0.590	-0.04565 (-0.70111 to 0.60981)	0.883	0.16936 (-0.25995 to 0.59868)	0.409
ΔInfluenza +	-0.00964 (-0.02252 to 0.00324)	0.883	-0.00876 (-0.02738 to 0.00985)	0.328	-0.00976 (-0.02033 to 0.00081)	0.068
ΔInfluenza +, lag 1	-0.01999 (-0.04573 to 0.00574)	0.117	-0.01837 (-0.04871 to 0.01197)	0.214	-0.01298 (-0.04073 to 0.014772)	0.331
ΔInfluenza –	0.00194 (-0.02614 to 0.03003)	0.130	-0.01531 (-0.04658 to 0.01596)	0.309	0.02155 (-0.00343 to 0.04653)	0.085
ΔInfluenza –, lag 1	0.00864 (-0.00271 to 0.02000)	0.124	0.01033 (-0.00294 to 0.02359)	0.116	0.00585 (-0.00565 to 0.01735)	0.292
**Long-term effect**						
Influenza +	0.01255	0.618	-0.02659	0.541	0.04091	0.064
Influenza –	-0.01033	0.684	0.024538	0.582	-0.02870	0.184
**Model diagnostics**						
Q-test for autocorrelation, χ^2	5.662	0.773	3.790	0.925	5.243	0.813

For peer review only - http://bmjopen.bmj.com/site/about/guidelines.xhtml

Supplementary material

Heteroscedasticity, χ^2	0.102	0.750	0.155	0.694	0.079	0.778
Normality, χ^2	1.569	0.456	1.627	0.443	1.237	0.537
RESET, F-statistics	5.839	0.014	3.348	0.064	1.089	0.398
CUSUM	Stable, no structural break	NA	Stable, no structural break	NA	Stable, no structural break	NA
CUSUMQ	Stable	NA	Stable	NA	Stable	NA
Adj. R^2	0.246	NA	0.337	NA	0.371	NA
$Wald_{SR}$, F-statistics	2.903	0.112	0.536	0.477	7.132	0.019
$Wald_{LR}$, F-statistics	0.922	0.354	0.297	0.595	26.74	<0.001
**Cointegration test statistics**						
t_BDM	-2.706	NA	-1.891	NA	-3.707	NA
F_PSS	2.649	NA	2.075	NA	4.688	NA
**Critical values for F_PSS**						
5% critical values; I(0), I(1)	k=1: 5.395, 6.350<br>k=2: 4.267, 5.473	NA	k=1: 5.395, 6.350<br>k=2: 4.267, 5.473	NA	k=1: 5.395, 6.350<br>k=2: 4.267, 5.473	NA

Note: F_PSS denotes F-statistics for Pesaran/Shin/Smith bounds test with null hypothesis of no cointegration. Critical values are retrieved from Narayan PK (2005) for a sample size of n=30 [1]. The null hypothesis of no cointegration is supported if F_PSS statistics is lower than 5% I(0) critical value; the null hypothesis is rejected if F_PSS statistics is greater than 5% I(1) critical value. We applied a conservative approach to considering the number of exposure variables (k), as suggested by Shin Y, et al (2014) [2] (since influenza death rates represent one exposure (k=1), while the analysis actually partitions the exposure to positive and negative changes (k=2)). In a given table, the results of F_PSS for the whole population, men, and women are lower than the reported 5% I(0) critical values that accepts a null hypothesis of no cointegration.

For peer review only - http://bmjopen.bmj.com/site/about/guidelines.xhtml

Supplementary material

"LnSuicide" indicates that suicide rates were logarithmically-transformed. "Δ" signifies the series is differenced. Signs as "+" and "–" denote the exposure variable being partitioned in positive and negative changes, respectively. "NA" denotes that a certain test or test parameter is not applicable.

Number of lags used for each variable in the model are noted by "lag#". To select the optimal number of lags to be used for choosing p and q parameters for the NARDL (i.e., numbers of lags for dependent and independent variables, respectively), we applied a varsoc command in STATA using the minimal values of Akaike Information Criterion, Schwarz's Bayesian information criterion (SBIC), and the Hannan and Quinn information criterion information criteria. If information criteria indicated different lag orders, SBIC was used to select optimal lags. Thus, for the analysis of the whole population, men, and women, both exposure and outcome time series were lagged once.

Wald test for asymmetry in a short-term ($Wald_{SR}$) and long-term ($Wald_{LR}$) has a null hypothesis of no cointegration. Q-test for autocorrelation reports the results of Portmanteau test for white noise. Heteroscedasticity is measured by Breusch-Pagan/Cook-Weisberg test. Normality is measured by Jarque-Bera test. RESET statistics refers to the regression specification error tests. CUSUM and CUSUMQ refer to the tests of the cumulative sum of recursive residuals and their squares, respectively. "Stable, no structural break" in the results of CUSUM indicates that the model was found stable and the null hypothesis of no structural break was not rejected (same applies to "Stable" as an output for CUSUMQ).

REFERENCES

1 Narayan PK. The saving and investment nexus for China: evidence from cointegration tests. *Applied Economics* 2005; **37**(17): 1979–1990.

2 Shin Y, Yu B, Greenwood-Nimmo M. Modelling Asymmetric Cointegration and Dynamic Multipliers in a Nonlinear ARDL Framework. In: Sickles RC, Horrace WC, eds. Festschrift in Honor of Peter Schmidt: Econometric Methods and Applications. New York: Springer Science+Business Media, 2014: 281–314.

For peer review only - http://bmjopen.bmj.com/site/about/guidelines.xhtml

STROBE Statement—checklist of items that should be included in reports of observational studies

	Item No	Recommendation	Page No
**Title and abstract**	1	(*a*) Indicate the study's design with a commonly used term in the title or the abstract	1
		(*b*) Provide in the abstract an informative and balanced summary of what was done and what was found	2
**Introduction**			
Background/rationale	2	Explain the scientific background and rationale for the investigation being reported	3
Objectives	3	State specific objectives, including any prespecified hypotheses	3
**Methods**			
Study design	4	Present key elements of study design early in the paper	3-4
Setting	5	Describe the setting, locations, and relevant dates, including periods of recruitment, exposure, follow-up, and data collection	3-5
Participants	6	(*a*) *Cohort study*—Give the eligibility criteria, and the sources and methods of selection of participants. Describe methods of follow-up<br>*Case-control study*—Give the eligibility criteria, and the sources and methods of case ascertainment and control selection. Give the rationale for the choice of cases and controls<br>*Cross-sectional study*—Give the eligibility criteria, and the sources and methods of selection of participants	3-5
		(*b*) *Cohort study*—For matched studies, give matching criteria and number of exposed and unexposed<br>*Case-control study*—For matched studies, give matching criteria and the number of controls per case	n/a
Variables	7	Clearly define all outcomes, exposures, predictors, potential confounders, and effect modifiers. Give diagnostic criteria, if applicable	3-4
Data sources/ measurement	8*	For each variable of interest, give sources of data and details of methods of assessment (measurement). Describe comparability of assessment methods if there is more than one group	3-4
Bias	9	Describe any efforts to address potential sources of bias	4
Study size	10	Explain how the study size was arrived at	4
Quantitative variables	11	Explain how quantitative variables were handled in the analyses. If applicable, describe which groupings were chosen and why	5
Statistical methods	12	(*a*) Describe all statistical methods, including those used to control for confounding	5
		(*b*) Describe any methods used to examine subgroups and interactions	5
		(*c*) Explain how missing data were addressed	n/a
		(*d*) *Cohort study*—If applicable, explain how loss to follow-up was addressed<br>*Case-control study*—If applicable, explain how matching of cases and controls was addressed<br>*Cross-sectional study*—If applicable, describe analytical methods taking account of sampling strategy	n/a
		(*e*) Describe any sensitivity analyses	n/a

Continued on next page

For peer review only - http://bmjopen.bmj.com/site/about/guidelines.xhtml

Results			
Participants	13*	(a) Report numbers of individuals at each stage of study—eg numbers potentially eligible, examined for eligibility, confirmed eligible, included in the study, completing follow-up, and analysed	n/a
		(b) Give reasons for non-participation at each stage	n/a
		(c) Consider use of a flow diagram	n/a
Descriptive data	14*	(a) Give characteristics of study participants (eg demographic, clinical, social) and information on exposures and potential confounders	5-7
		(b) Indicate number of participants with missing data for each variable of interest	n/a
		(c) *Cohort study*—Summarise follow-up time (eg, average and total amount)	n/a
Outcome data	15*	*Cohort study*—Report numbers of outcome events or summary measures over time	6-7
		Case-control study—Report numbers in each exposure category, or summary measures of exposure	
		Cross-sectional study—Report numbers of outcome events or summary measures	
Main results	16	(*a*) Give unadjusted estimates and, if applicable, confounder-adjusted estimates and their precision (eg, 95% confidence interval). Make clear which confounders were adjusted for and why they were included	5-7, and 13-15
		(*b*) Report category boundaries when continuous variables were categorized	
		(*c*) If relevant, consider translating estimates of relative risk into absolute risk for a meaningful time period	
Other analyses	17	Report other analyses done—eg analyses of subgroups and interactions, and sensitivity analyses	
**Discussion**			
Key results	18	Summarise key results with reference to study objectives	7
Limitations	19	Discuss limitations of the study, taking into account sources of potential bias or imprecision. Discuss both direction and magnitude of any potential bias	8
Interpretation	20	Give a cautious overall interpretation of results considering objectives, limitations, multiplicity of analyses, results from similar studies, and other relevant evidence	7-8
Generalisability	21	Discuss the generalisability (external validity) of the study results	8
**Other information**			
Funding	22	Give the source of funding and the role of the funders for the present study and, if applicable, for the original study on which the present article is based	9

*Give information separately for cases and controls in case-control studies and, if applicable, for exposed and unexposed groups in cohort and cross-sectional studies.

Note: An Explanation and Elaboration article discusses each checklist item and gives methodological background and published examples of transparent reporting. The STROBE checklist is best used in conjunction with this article (freely available on the Web sites of PLoS Medicine at http://www.plosmedicine.org/, Annals of Internal Medicine at http://www.annals.org/, and Epidemiology at http://www.epidem.com/). Information on the STROBE Initiative is available at www.strobe-statement.org.

For peer review only

BMJ Open

A Swedish nationwide time series analysis of influenza and suicide deaths from 1910 to 1978

Journal:	*BMJ Open*
Manuscript ID	bmjopen-2021-049302.R2
Article Type:	Original research
Date Submitted by the Author:	17-Jun-2021
Complete List of Authors:	Rück, Christian; Karolinska Institutet, Department of Clinical Neuroscience, Center for Psychiatry Research<br>Mataix-Cols, David; Karolinska Institutet, Department of Clinical Neuroscience, Center for Psychiatry Research<br>Malki, Kinda; Karolinska Institutet<br>Adler, Mats; Karolinska Institutet<br>Flygare, Oskar; Karolinska Institutet<br>Runeson, Bo; Karolinska Institutet, Clin Neuroscience<br>Sidorchuk, Anna; Karolinska Institutet Department of Clinical Neuroscience,
**Primary Subject Heading**:	Mental health
Secondary Subject Heading:	Epidemiology, Public health
Keywords:	MENTAL HEALTH, COVID-19, Suicide & self-harm < PSYCHIATRY

SCHOLARONE™
Manuscripts

I, the Submitting Author has the right to grant and does grant on behalf of all authors of the Work (as defined in the below author licence), an exclusive licence and/or a non-exclusive licence for contributions from authors who are: i) UK Crown employees; ii) where BMJ has agreed a CC-BY licence shall apply, and/or iii) in accordance with the terms applicable for US Federal Government officers or employees acting as part of their official duties; on a worldwide, perpetual, irrevocable, royalty-free basis to BMJ Publishing Group Ltd ("BMJ") its licensees and where the relevant Journal is co-owned by BMJ to the co-owners of the Journal, to publish the Work in this journal and any other BMJ products and to exploit all rights, as set out in our licence.

The Submitting Author accepts and understands that any supply made under these terms is made by BMJ to the Submitting Author unless you are acting as an employee on behalf of your employer or a postgraduate student of an affiliated institution which is paying any applicable article publishing charge ("APC") for Open Access articles. Where the Submitting Author wishes to make the Work available on an Open Access basis (and intends to pay the relevant APC), the terms of reuse of such Open Access shall be governed by a Creative Commons licence – details of these licences and which Creative Commons licence will apply to this Work are set out in our licence referred to above.

Other than as permitted in any relevant BMJ Author's Self Archiving Policies, I confirm this Work has not been accepted for publication elsewhere, is not being considered for publication elsewhere and does not duplicate material already published. I confirm all authors consent to publication of this Work and authorise the granting of this licence.

For peer review only

Word count

Abstract: 231
Body: 3532
Tables: 3
Figures: 2

A Swedish nationwide time series analysis of influenza and suicide deaths from 1910 to 1978

Christian Rück, M.D.[1], David Mataix-Cols, Ph.D.[1], Kinda Malki, M.D. [1], Mats Adler, M.D. [1], Oskar Flygare, M.Sc.[1], Bo Runeson, M.D. [1], Anna Sidorchuk, M.D.[1]

Corresponding author:
Professor Christian Rück, M46, Karolinska University Hospital Huddinge, SE-14186 Huddinge, Sweden E-mail: Christian.ruck@ki.se, telephone +46704843392.

Affiliation:
1. Centre for Psychiatry Research, Department of Clinical Neuroscience,
Karolinska Institutet, & Stockholm Health Care Services, Region Stockholm, Sweden

For peer review only

ABSTRACT

Objectives: There is concern that the COVID-19 pandemic will be associated with an increase in suicides, but evidence supporting a link between pandemics and suicide is limited. Using data from the three influenza pandemics of the 20th century, we aimed to investigate whether an association exists between influenza deaths and suicide deaths.

Design: Time series analysis.

Setting: Sweden.

Participants: Deaths from influenza and suicides extracted from the Statistical Yearbook of Sweden for 1910-1978, covering three pandemics (the Spanish flu, the Asian flu and the Hong-Kong flu).

Main outcome measures: Annual suicide rates in Sweden among the whole population, men, and women. Non-linear autoregressive distributed lag (NARDL) models was implemented to explore if there is a short-term and/or long-term relationship of increases and decreases in influenza death rates with suicide rates during 1910-1978.

Results: Between 1910-1978, there was no evidence of either short-term or long-term significant associations between influenza death rates and changes in suicides (β-coefficients of 0.00002, P=0.931 and β=0.00103, P=0.764 for short-term relationship of increases and decreases in influenza death rates, respectively, with suicide rates, and β=-0.0002, P=0.998 and β=0.00211, P=0.962 for long-term relationship of increases and decreases in influenza death rates, respectively, with suicide rates). The same pattern emerged in separate analyses for men and women.

Conclusions: We found no evidence of short or long-term association between influenza death rates and suicide death rates across three 20th century pandemics.

Key words: pandemics, suicide, mental health, Spanish flu, Asian influenza, Hong-Kong influenza, COVID-19

Strengths and limitations of this study

- To our knowledge, this was the first study to investigate the association between influenza deaths and suicide across several pandemics.
- The large amount of nationwide data on influenza and suicide death rates covering 1910-1978 is a strength of the study.
- To guard against effects of changes in the recording of causes of death, we created a series of dummy variables for each corresponding period but no significant effects were found.
- No historical data with higher temporal resolution than yearly data could affect the results if the time sequence of association between changes in influenza death and suicide differ from the chosen time interval.

For peer review only

INTRODUCTION

Various international surveys have documented a negative impact of the COVID-19 pandemic on the population's mental health, with increased levels of psychological stress, psychiatric symptoms, insomnia, and alcohol consumption.[1–4] Whether these acute impacts will persist long term is currently unknown. The Royal College of Psychiatrists in the UK and the World Health Organization as well as the International Academy of Suicide Research have raised concerns of a possible increase in suicide rates.[5–7] Such concerns originate from a combination of known risk factors for suicide, including the impacts of social distancing and disconnectedness, an economic downturn, the decreased access to mental health services, and increased access to lethal means exemplified by an increase in gun purchases in the US.[8] For example, a study of the economic recession in USA 2007-2009 found that for every percentage point increase in the unemployment rate, there was about a 1.6 per cent increase in the suicide rate.[9,10] These findings have been questioned. A study using an interrupted time-series analysis taking e.g. seasonality and long-term trends into account, found little evidence that the recession resulted in net excess suicides across all age and gender groups.[11] However, the was some evidence of excess suicides among men aged 65 and above 65 and young women. Another study failed to find an increase in suicide rates in Sweden during the two most recent economic recessions.[12]

While the concern is widespread, there is currently little evidence to support a clear association between the ongoing COVID-19 outbreak and an increased risk of suicide. Data from the US Centers for Disease Control and Prevention shows that suicide decreased in 2020, compared to the preceding year.[13] Data from April to October 2020 from the UK did not show an increase of suicides.[14] The first months of the COVID-19 pandemic in 21 countries was studied by Pirkis and collegues and overall there was little support for an increase in suicides.[15] Other reports highlight that the outcomes differ and that certain minorities may be at higher risk.[16]

Historic US mortality data from the largest pandemic in the 20th century, the Spanish flu, showed that the Spanish flu was associated with an increase in suicides but those effects may have been mitigated by a decline in alcohol consumption.[17] Suicide during the same pandemic in Taiwan, where the Japanese Colonial Government implemented physical distancing, school closings and prohibited religious activities, was studied in a paper by Chang and collegues.[18] They reported a small and shortlived increase in suicides in the second wave of the pandemic. A study of the impact of social distancing measures on suicide in 1918 in the US was studied and showed that increasing social distancing was associated with suicide rates independent of the influenza mortality rate.[19] In Sweden, there were measures in place in Sweden during the Spanish Flu to minimize the spread of the disease, such as school closings, and public gatherings, cinemas and religious meetings were temporarily stopped in some places.[20]

The outbreak of the Severe Acute Respiratory Syndrome (SARS) epidemic in 2003 in Hong Kong was according to two studies associated with an increase of suicide in the population above the age of 65.[21,22] In the first of these two reports, the association was only statistically significant in elderly women.[21] Another study of suicides during SARS suggested that disconnectedness and fear of contracting SARS were more prevalent in older

For peer review only - http://bmjopen.bmj.com/site/about/guidelines.xhtml

adult SARS-related suicide victims than non-SARS-related suicide victims. However, this was based on a small number of suicides.[23] To summarise, at present, our understanding of the effects of pandemics on suicide rates is very limited.

The availability of high-quality historical data on mortality due to influenza and suicide in Sweden provides a unique opportunity to examine this important question. While we at present have some short-term data on the rates of suicide during the COVID-19 pandemic, a better understanding of what may be the outcome in the longer run would be valuable. Using historical data from the three major influenza pandemics of the 20th century, we aimed to formally investigate whether an association between influenza deaths and suicide deaths exists. We hypothesize that such association, if any, would likely be weak. Given the substantial sex differences in suicidal behaviour and the studies suggesting sex-specific effects during SARS,[21] we additionally report on associations separately in men and women.

METHODS

Data acquisition

Annual data on influenza death rates and suicide rates were estimated based on information from the Statistical Yearbook of Sweden from 1910-1978.[24] Over this period, Sweden experienced three influenza pandemics which occurred during different socio-political contexts, namely the Spanish flu (1918-1920, with the first case appearing in Sweden in June 1918), the Asian flu (1957-1958, being first documented in August 1957), and the Hong Kong flu (1968-1969, starting in the autumn of 1968).[20] The yearbooks were produced by Statistics Sweden, a governmental agency responsible for the official statistics in Sweden, with a history of population statistics going back to the 18th century.[24] For each year from 1910 to 1978, we retrieved information on the total population of Sweden, the number of deaths by influenza (if death cause was indicated as "influenza") and the number of suicides (if death cause was indicated as "suicide"), as well as the corresponding data separately for men and women. Since yearbooks reported data retrospectively for several years prior to the year each book was published, we checked the correctness of retrieved data by comparing yearbooks with overlapping reporting periods. We constructed influenza mortality rates and suicide rates per 100 000 inhabitants for each year by dividing the number of deaths by influenza and, separately, suicide, by the total number of individuals registered in Sweden in a corresponding year and then multiplying by 100 000. The corresponding rates for men and women were constructed likewise. In addition, we collected information on the changes in registration of deaths in Sweden that included the cause of death classification based on the Bertillon criteria (prior to 1931), the new classification introduced in cooperation with statistical authorities from other Nordic countries (1931-1950), the International Classification of Diseases (ICD) Sixth Revision (ICD-6; 1951-1957), ICD-7 (1958-1968), and ICD-8 (1969-1978).[25] To capture a potential effect of changes in classification, we created a series of dummy variables for each corresponding period, but these were only kept in the models if statistically significant.

The study protocol was not preregistered. The full dataset used in the analysis of historical data (1910-1978) and the STATA code are available in the online Supplement (**Supplementary eTable 1** and **Supplementary material**).

Statistical Analyses

For peer review only - http://bmjopen.bmj.com/site/about/guidelines.xhtml

We used the historical data 1910-1978 that includes three pandemics and focused on exploring a possible asymmetric short-term effect of influenza death rates on suicide (i.e., immediate or instantaneous effect) and long-term effect (i.e., if the relationship of exposure and outcome has a lag-structure, or in other words, if the effect of exposure on outcome is distributed over a longer period of time) by applying non-linear autoregressive distributed lag (NARDL) models,[26] a technique initially introduced for research in economics. NARDL models split the exposure in partial sum of positive changes (i.e, increases in influenza death rates) and partial sum of negative changes (i.e., decreases in influenza death rates) and explore if an outcome responds differently (i.e., asymmetrically) to an increase and decrease in exposure variable.[26] In other words, NARDL models do not rely on an assumption of symmetrical effects, by which the association of the outcome with a unit of positive change in the exposure is expected to be equal in strength and opposite in direction to the association between the outcome and a unit of negative change in the exposure. We performed the modelling by using the *nardl* command in STATA.

Before the NARDL model is executed, it is important to test that the conditions for the modelling are fulfilled. It starts from testing whether or not the variables are stationary (i.e., their means and variances are constant over time). The advantage of the NARDL modelling is that it can be used regardless of whether the variables are integrated of the order zero, which means that variables are stationary, integrated of the order one, which indicates non-stationarity, or mixed.[26] We examined the stationarity properties of the variables by using the Augmented Dickey–Fuller unit-root test (ADF) and Kwiatkowski-Phillips-Schmidt-Shin test for stationarity (KPSS) to explore the order of the integration for influenza death rates and suicide rates separately for the total population, men, and women. Previous studies on suicides hypothesized that various suicidogenic factors may interplay and reinforce each other and the logarithmic transformation of suicide time series was suggested.[27,28] We made such transformation by expressing suicide rates in natural logarithm. To select the optimal number of lags (i.e., how far in the past the dependency among measurements is examined) to be used in the NARDL for dependent and independent variables, we applied the *varsoc* command in STATA using the minimal values of Akaike Information Criterion, Schwarz's Bayesian information criterion (SBIC), and the Hannan and Quinn information criterion information criteria. If information criteria indicated different lag orders, SBIC was used to select optimal lags. We applied the NARDL model to (i) estimate the coefficients and the corresponding 95% confidence intervals for short-term and long-term association of suicide rates with an increase and decrease in influenza death rates; (ii) explore if there is a long-term cointegration between exposure and outcome by using a bounds test (if variables are cointegrated, it means their positive and negative components do not drift far away from each other in the longer term); and (iii) obtain Wald test statistics that specifies whether or not short-term and/or long-term relation between the exposure and outcome is asymmetrical. Model diagnostic tests are described in the **Supplementary material**.

The modelling was performed, first, for the whole period of 1910-1978, and then for the periods of 1918-1956 (from the beginning of the Spanish influenza pandemic to the year before the Asian influenza pandemic), and for 1957-1978 (from the beginning of Asian pandemic to the end of observation period; with this interval also covering the Hong Kong influenza pandemic).

All statistical analyses were performed using STATA version 15.1 (StataCorp LLC, College Station, TX, USA).

For peer review only - http://bmjopen.bmj.com/site/about/guidelines.xhtml

Patient and public involvement
Patients and members of the public were not directly involved as this study used publically available historical national mortality data.

RESULTS

Over the period 1910-1978, influenza death rates fluctuated considerably in Sweden with the highest rates being observed during the Spanish flu (in 1918, 1919, and 1920, the rates were 470.93, 125.55, and 48.32 per 100 000 inhabitants, respectively) (**Figure 1**). In post-pandemic years, several noticeable peaks in influenza death rates appeared, with the ones in 1922, 1927, 1929, 1931, 1937, and 1941 being particularly high. In the following years, a considerable fluctuation in influenza mortality remained, although the rates during the periods of the Asian flu (in 1957 and 1958: 8.78 and 3.99 per 100 000 inhabitants, respectively) and the Hong Kong flu (in 1968 and 1969: 3.67 and 4.68 per 100 000 inhabitants, respectively) were lower than that in the first half of the century. The influenza death rates were very similar in men and women across the entire observation period (**Figure 2**).

[Figure 1 to be inserted here]

[Figure 2 to be inserted here]

During the same period, a total of 80 058 deaths due to suicide occurred in Sweden (60 713 in men and 19 345 in women). The average suicide rate across 1910-1978 was 16.79 per 100 000 inhabitants (standard deviation [SD] of 2.58), with corresponding rates for men and women as 25.85 (SD=3.37) and 7.91 (SD=2.26) per 100 000, respectively. There was a considerable fluctuation in the suicide rates over time (**Figure 1**). The initial decrease in suicide rates during 1913-1918, with the lowest rate of 9.97 per 100 000 in 1918, was followed by an increase with the highest peak of 22.15 per 100 000 reached in 1970, with a series of intermediate peaks.

Sex-specific suicide rates differed considerably with the rates among men being between twice to over four times higher than that in women (on average, 3.4-times higher) (**Figure 2**). Moreover, suicide rates in men exhibited a sharp dip in 1913-1918, which was first followed by an increase by 1921 and then continued to raise more gradually, although with several distinct peaks and, rarely, dips. Among women, an upward trend in suicide rate was present, although with less fluctuations than that in men.

In our analysis of 1910-1978 data, the first step with the use of the ADF and KPSS test statistics on variables' stationarity indicated that the logarithmically-transformed suicide rates were integrated of the order one (for all, and men and women separately); whereas the influenza death rates were integrated of the order zero (for all and for men and women separately) (**Supplementary eTable 2)**. On this premise, we implemented the NARDL modelling. Dummy variables on changes in death registration did neither appear statistically significant nor affected any parameter estimates in the initial model, and thus were not included in the final models. As reported in **Table 1**, for the observation period of 1910-1978, there were no statistically significant associations (either short-term or long-term) between decreased or increased influenza death rates and suicide rates among the overall population and among men. The corresponding results for women indicated a possible short-term association whereby a decrease in influenza rates seemed to be associated with a borderline

For peer review only - http://bmjopen.bmj.com/site/about/guidelines.xhtml

decrease in suicides; however, the findings were not supported by the Wald test for asymmetry ([$Wald_{SR}$], i.e. the null hypothesis of $Wald_{SR}$ that an increase and decrease in influenza death rates would symmetrically affect suicide rates was not rejected). This suggests that association was likely due to the effect of other unobserved factors that may influence suicides in women. Full specification of the models is reported in **Supplementary eTable 3**. Full specification also includes the results of testing for long-term cointegration between influenza mortality and suicides (as reported by F-statistics for Pesaran/Shin/Smith bounds test [F_PSS]). Long-term cointegration was not found as we were unable to reject the null hypothesis of no cointegration between variables (**Supplementary eTable 3,** see footnotes for details on bounds test for cointegration).

The results of the additional analyses for the periods 1918-1956 and 1957-1978 also failed to provide clear evidence of association between changes in influenza mortality rates and suicide rates in either short-term or long-term. However, in the analysis among women, a long-term asymmetry was suggested in both periods (the Wald test for asymmetry in the long-term [$Wald_{LR}$] $p=0.044$ and $p<0.001$ in the analyses of 1918-1956 and 1957-1978, respectively) and a short-term asymmetry in the analysis of 1957-1978 ($Wald_{SR}$ test $p=0.019$) (**Tables 2** and **3**). We assume that these findings may again reflect the influence of unobserved confounders, as these results were not supported by other coefficients, which correspond to long- and short-term associations between increases and decreases in influenza deaths and suicides among women in 1918-1956 and 1957-1978 (**Tables 2** and **3**). It is important to mention that the results for 1957-1978 should be considered with caution since the analysis included time series with only 22 observations. Full specifications for the models used in the analyses of 1918-1956 and 1957-1978 are reported in **Supplementary eTable 4** and **5**. No evidence of long-term cointegration between influenza and suicide rates were found in either period (F_PSS statistics does not reject the null hypothesis in the analyses of the whole population, men, and women; **Supplementary eTable 4** and **5,** see footnotes for details on bounds test for cointegration). Overall, the model diagnostics for all models for 1910-1978, 1918-1956, and 1957-1978 time series support the validity of the results since, with very few exceptions, all diagnostic tests (for details see Supplementary materials) are insignificant indicating that there is no autocorrelation, heteroscedasticity, misspecification and non-normality In addition, the tests of the cumulative sum of recursive residuals and their squares (CUSUM and CUSUMQ, respectively) for all models indicate stability and absence of structural breaks (for details see footnotes for **Table 1-3** and **Supplementary eTable 3-5**).

For peer review only

DISCUSSION

This study used publicly available Swedish national data from 1910 to 1978 to shed light on the potential association between influenza-related deaths and over 80 000 deaths by suicide across the three 20th century pandemics. To our knowledge, this is the first study of influenza death and suicide that analyses data from several influenza pandemics. The full modelling provided no clear evidence of either a short-term or a long-term relationship between changes in influenza death rates and changes in suicide rates. The year with the highest number of influenza-related deaths by far, 1918, had the lowest number of suicides in the whole time series.

The findings in context

For peer review only - http://bmjopen.bmj.com/site/about/guidelines.xhtml

International historical data on pandemics and suicide are very scarce. A report from the US focusing on the years 1910-1920 and using monthly data suggested a possible association between influenza deaths and suicide but because the data did not go beyond the last year of that pandemic, it does not inform us about any longer term effects.[17] Strengths of that study included the use of monthly data and the use of time series analysis, rather than just comparing suicide rates before and after the pandemic. Chang et al studied suicides during the same pandemic in Taiwan, not using a time series analysis but also using monthly data from 1918-20 and found an increase during the few months of the second vawe of the pandemic but that the effect was short-lived. As our study used yearly and not monthly data, it could not confirm or disconfirm the findings of the Taiwanese study. Our study expands and improves upon previous investigations because it allowed us to examine the long-term associations between influenza and suicide deaths. A study focusing on SARS and suicide found some evidence of sex-specific effects in the short-term.[21] As suicide rates varied considerably in the years preceding SARS in Hong Kong, it is difficult to be certain that SARS, and not other contributing factors, was causally associated with an increase in suicides. In the present study, which uniquely spanned over several decades, we found that while suicide was consistently more prevalent among men throuought the study period, there were no sex-specific associations to influenza death.

Meaning of the study

What do these results mean for our understanding of the short- and long-term consequences of influenza pandemics? Our results do not support the belief that global pandemics necessarily lead to a substantial increase in suicides. There are many factors associated with a global pandemic that may potentially increase suicide risk in the population, but these may be offset by other protective factors that should not be overlooked. A shared sense of belonging and focus, social connectedness and a "pulling together effect" may be one such factor.[1,29] We acknowledge that these historical findincs may not be directly applicable to the current COVID-19 pandemic because there are several important differences between the previous and current pandemics, such as globalisation, mortality rates, a different socio-political context and the impact of social media, to name a few.

Strengths and limitations

The major strengths of this study were the use of a large amount of historical national data across three 20th century pandemics and state of the art time-series analyses. A limitation is that all data comes from a single country and and thus caution is needed when generalising the study findings to countries with different level of development of clinical and preventive medicine, social support, etc.The study used the Swedish historical public death records and we had no means of verifying the causes of death. The coverage and precision of these records is likely to have improved over time for both variables. To guard against effects of changes in the recording of causes of death, we created a series of dummy variables for each corresponding period but no significant effects of those were found. If there were some other time-varying factors that could affect the coverage and precision of death records, apart from the official changes in registration system, this might have biased our results, in particular if such factors differentially affected the quality of recording deaths due to influenza and suicide. As we did not have access to data with higher temporal resolution than yearly data, that could affect our results if the time sequence of association between changes in influenza death and suicide differ from the chosen time interval. A multitude of factors may vary that impact the resilience of the society with regards to the effect of a pandemic. Such factors may also vary over time and place. However, the fact that we observed no clear associations between influenza and suicide deaths across pandemics, which challenged society with

For peer review only - http://bmjopen.bmj.com/site/about/guidelines.xhtml

various degrees of e.g. economic effects and health care supply issues, does support the stability of our findings.

Conclusion

In this national analysis of historical data spanning across three 20th century pandemics, we found no evidence of a short- or long-term association between influenza death rates and suicide rates. The results challenge the notion that an increase of suicides follows as a certain consequence of a pandemic. Future research on the effect of the current COVID-19 pandemic should explore the possibility of differential short- and long-term effects on suicide rates in different subgroups of the population.

Footnotes

Contributor statement: CR and AS have full access to all the data in this study and take full responsibility as guarantors for the integrity of the data and the accuracy of the data analysis. CR, DMC, and AS conceived and designed the study. CR, KM, MA, and AS performed data collection. AS undertook the statistical analysis. CR, DMC, and AS drafted the manuscript. CR, AS, DMC, KM, MA, BR and OF provided critical input to the analyses, interpreted the data, and revised the manuscript critically. The corresponding author confirms that all listed authors meet authorship criteria and that no others meeting the criteria have been omitted.

Funding: Karolinska Institutet and Region Stockholm, which provide salary for the authors, had no involvement in the study design; collection, analysis, and interpretation of data; in the writing of the report; or in the decision to submit the paper for publication.

Competing interests: All authors have completed the ICMJE uniform disclosure form at www.icmje.org/coi_disclosure.pdf and declare: no grant support from any organisation for the submitted work, salaries of the author were provided from Region Stockholm and Karolinska Institutet but not specifically for this project; no financial relationships with any organisations that might have an interest in the submitted work in the previous three years; no other relationships or activities that could appear to have influenced the submitted work.

Data sharing: The full dataset (numbers of deaths by influenza and suicide and total population for 1910-1978) is published as supplementary material (eTable 1).

Dissemination to participants and patient organisations: since only aggregated mortality data was used, dissemination to participants is not possible. We intend to disseminate the results to the general public via media and the research group's webpage.

Transparency declaration: The lead author affirms that this manuscript is an honest, accurate, and transparent account of the study being reported; that no important aspects of the study have been omitted; and that any discrepancies from the study as planned have been explained.

Ethical approval: Since only data already available in the public domain were used, ethical approval was not necessary.

For peer review only

References

1 Reger MA, Stanley IH, Joiner TE. Suicide Mortality and Coronavirus Disease 2019—A Perfect Storm? *JAMA Psychiatry* Published Online First: 10 April 2020. doi:10.1001/jamapsychiatry.2020.1060

2 Dutheil F, Mondillon L, Navel V. PTSD as the second tsunami of the SARS-Cov-2 pandemic. *Psychol Med* 2020;:1–2. doi:10.1017/S0033291720001336

3 O'Connor RC, Wetherall K, Cleare S, *et al.* Mental health and well-being during the COVID-19 pandemic: longitudinal analyses of adults in the UK COVID-19 Mental Health & Wellbeing study. *Br J Psychiatry* 2020;:1–8. doi:10.1192/bjp.2020.212

4 Pierce M, Hope H, Ford T, *et al.* Mental health before and during the COVID-19 pandemic: a longitudinal probability sample survey of the UK population. *Lancet Psychiatry* 2020;**7**:883–92. doi:10.1016/S2215-0366(20)30308-4

5 WHO. Substantial investment needed to avert mental health crisis. https://www.who.int/news-room/detail/14-05-2020-substantial-investment-needed-to-avert-mental-health-crisis (accessed 1 Jun 2020).

6 Zalsman G, Stanley B, Szanto K, *et al.* Suicide in the Time of COVID-19: Review and Recommendations. *Arch Suicide Res* 2020;:1–6. doi:10.1080/13811118.2020.1830242

7 Psychiatrists see alarming rise in patients needing urgent and emergency care and forecast a 'tsunami' of mental illness. www.rcpsych.ac.uk. https://www.rcpsych.ac.uk/news-and-features/latest-news/detail/2020/05/15/psychiatrists-see-alarming-rise-in-patients-needing-urgent-and-emergency-care (accessed 1 Jun 2020).

8 Moutier C. Suicide Prevention in the COVID-19 Era: Transforming Threat Into Opportunity. *JAMA Psychiatry* Published Online First: 16 October 2020. doi:10.1001/jamapsychiatry.2020.3746

9 Stuckler D, Basu S, Suhrcke M, *et al.* The public health effect of economic crises and alternative policy responses in Europe: an empirical analysis. *The Lancet* 2009;**374**:315–23. doi:10.1016/S0140-6736(09)61124-7

10 Phillips JA, Nugent CN. Suicide and the Great Recession of 2007–2009: The role of economic factors in the 50 U.S. states. *Soc Sci Med* 2014;**116**:22–31. doi:10.1016/j.socscimed.2014.06.015

11 Harper S, Bruckner TA. Did the Great Recession increase suicides in the USA? Evidence from an interrupted time-series analysis. *Ann Epidemiol* 2017;**27**:409-414.e6. doi:10.1016/j.annepidem.2017.05.017

12 Reeves A, McKee M, Stuckler D. Economic suicides in the Great Recession in Europe and North America. *Br J Psychiatry* 2014;**205**:246–7. doi:10.1192/bjp.bp.114.144766

13 Ahmad FB, Anderson RN. The Leading Causes of Death in the US for 2020. *JAMA* Published Online First: 31 March 2021. doi:10.1001/jama.2021.5469

14 Appleby L, Richards N, Ibrahim S, *et al.* Suicide in England in the COVID-19 pandemic: Early observational data from real time surveillance. *Lancet Reg Health - Eur* 2021;:100110. doi:10.1016/j.lanepe.2021.100110

15 Pirkis J, John A, Shin S, *et al.* Suicide trends in the early months of the COVID-19 pandemic: an interrupted time-series analysis of preliminary data from 21 countries. *Lancet Psychiatry* Published Online First: 13 April 2021. doi:10.1016/S2215-0366(21)00091-2

For peer review only - http://bmjopen.bmj.com/site/about/guidelines.xhtml

16 Mitchell TO, Li L. State-Level Data on Suicide Mortality During COVID-19 Quarantine: Early Evidence of a Disproportionate Impact on Racial Minorities. *Psychiatry Res* 2021;**295**:113629. doi:10.1016/j.psychres.2020.113629
17 Wasserman IM. The impact of epidemic, war, prohibition and media on suicide: United States, 1910-1920. *Suicide Life Threat Behav* 1992;**22**:240–54.
18 Chang Y-H, Chang S-S, Hsu C-Y, *et al.* Impact of Pandemic on Suicide: Excess Suicides in Taiwan During the 1918–1920 Influenza Pandemic. *J Clin Psychiatry* 2020;**81**. doi:10.4088/JCP.20l13454
19 Stack S, Rockett IRH. Social distancing predicts suicide rates: Analysis of the 1918 flu pandemic in 43 large cities, research note. *Suicide Life Threat Behav* 2021;:sltb.12729. doi:10.1111/sltb.12729
20 Holmberg M. The ghost of pandemics past: revisiting two centuries of influenza in Sweden. *Med Humanit* 2017;**43**:141–7. doi:10.1136/medhum-2016-011023
21 Chan SMS, Chiu FKH, Lam CWL, *et al.* Elderly suicide and the 2003 SARS epidemic in Hong Kong. *Int J Geriatr Psychiatry* 2006;**21**:113–8. doi:10.1002/gps.1432
22 Cheung YT, Chau PH, Yip PSF. A revisit on older adults suicides and Severe Acute Respiratory Syndrome (SARS) epidemic in Hong Kong. *Int J Geriatr Psychiatry* 2008;**23**:1231–8. doi:10.1002/gps.2056
23 Yip PSF, Cheung YT, Chau PH, *et al.* The Impact of Epidemic Outbreak: The Case of Severe Acute Respiratory Syndrome (SARS) and Suicide Among Older Adults in Hong Kong. *Crisis* 2010;**31**:86–92. doi:10.1027/0227-5910/a000015
24 *Statistics Sweden. Statistical yearbook of Sweden [Statistisk årsbok för Sverige 1914–2014]. https://www.scb.se/hitta-statistik/aldre-statistik/innehall/statistisk-arsbok-1914-2014/.*
25 The National Board of Health and Welfare. Cause of death statistics. History, production methods and reliability [Dödsorsaksstatistik. Historik, produktionsmetoder och tillförlitlighet]. www.socialstyrelsen.se. 2010.
26 Shin Y, Yu B, Greenwood-Nimmo M. Modelling Asymmetric Cointegration and Dynamic Multipliers in a Nonlinear ARDL Framework. In: Sickles RC, Horrace WC, eds. *Festschrift in Honor of Peter Schmidt: Econometric Methods and Applications*. New York: : Springer Science+Business Media 2014. 281–314.
27 Norstrom T, Stickley A, Shibuya K. The importance of alcoholic beverage type for suicide in Japan: a time-series analysis, 1963-2007. *Drug Alcohol Rev* 2012;**31**:251–6. doi:10.1111/j.1465-3362.2011.00300.x
28 Gunnell D, Lopatatzidis A, Dorling D, *et al.* Suicide and unemployment in young people. Analysis of trends in England and Wales, 1921-1995. *Br J Psychiatry* 1999;**175**:263–70. doi:10.1192/bjp.175.3.263
29 Zortea TC, Brenna CTA, Joyce M, *et al.* The Impact of Infectious Disease-Related Public Health Emergencies on Suicide, Suicidal Behavior, and Suicidal Thoughts: A Systematic Review. *Crisis* 2020;:1–14. doi:10.1027/0227-5910/a000753

For peer review only

Figure legends

Figure 1. Annual suicide rate (scaled on the left arithmetic Y-axis) and influenza mortality (scaled on the right logarithmic Y-axis) in Sweden in 1910-1978 per 100 000 inhabitants. *Note*: To ease visualisation, influenza death rates are reported on the logarithmic scale, while for the analysis, the logarithmic transformation was performed for suicide rates. Grey areas denote the time periods corresponding to the Spanish influenza pandemic ('S.flu', 1918-1920), the Asian influenza pandemic ('A.flu', 1957-1958), and the Hong Kong influenza pandemic ('HK.flu', 1968-1969) in Sweden.

Figure 2. Sex-specific annual suicide rate (scaled on the left arithmetic Y-axis) and influenza mortality (scaled on the right logarithmic Y-axis) in Sweden in 1910-1978 per 100 000 inhabitants of corresponding sex. *Note*: To ease visualisation, influenza death rates are reported on the logarithmic scale, while for the analysis, the logarithmic transformation was performed for suicide rates. Grey areas denote the time periods corresponding to the Spanish influenza pandemic ('S.flu', 1918-1920), the Asian influenza pandemic ('A.flu', 1957-1958), and the Hong Kong influenza pandemic ('HK.flu', 1968-1969) in Sweden.

For peer review only

Table 1. Short-term and long-term relationship of suicide rates with positive and negative changes in influenza death rates among the whole population, men, and women in 1910-1978 in Sweden

	Whole population		Men		Women	
	**Coeff. (95% CI)**	**p-value**	**Coeff. (95% CI)**	**p-value**	**Coeff. (95% CI)**	**p-value**
**Short-term coefficients**						
Influenza +	0.00002 (-0.00036 to 0.00039)	0.931	0.00004 (-0.00034 to 0.00041)	0.854	-0.00007 (-0.00056 to 0.00041)	0.760
Influenza –	0.00103 (-0.00579 to 0.00785)	0.764	-0.00192 (-0.01005 to 0.00621)	0.638	0.00780 (0.00015 to 0.01544)	0.046
**Long-term coefficients**						
Influenza +	-0.00012	0.998	-0.01314	0.745	0.11254	0.538
Influenza –	0.00211	0.962	0.01443	0.722	-0.10789	0.544
**Model diagnostics**						
Q-test for autocorrelation, χ^2	40.160	0.125	35.260	0.273	31.850	0.424
Heteroscedasticity, χ^2	3.649	0.056	5.439	0.020	1.280	0.258
Normality, χ^2	2.826	0.243	2.237	0.327	2.372	0.305
RESET, F-statistics	4.656	0.006	5.473	0.023	1.013	0.394
CUSUM	Stabl., no str. break	NA	Stabl., no str. break	NA	Stabl., no str. break	NA
CUSUMQ	Stabl.	NA	Stabl.	NA	Stabl.	NA
Adj. R^2	0.257	NA	0.300	NA	0.374	NA
$Wald_{SR}$, F-statistics	0.054	0.817	0.275	0.602	1.062	0.307
$Wald_{LR}$, F-statistics	1.206	0.277	1.176	0.283	0.771	0.384

Note: Signs as "+" and "–" denote the exposure variable being partitioned in positive and negative changes, respectively. Wald test for asymmetry in the short-term ($Wald_{SR}$) and long-term ($Wald_{LR}$) has a null hypothesis of no cointegration. Q-test for autocorrelation reports the results of Portmanteau test for white noise. Heteroscedasticity is measured by Breusch-Pagan/Cook-Weisberg test. Normality is measured by Jarque-Bera test. RESET statistics refers to the regression specification error tests. CUSUM and CUSUMQ refer to the tests of the cumulative sum of recursive residuals and their squares, respectively. "NA" denotes that a certain test parameter was not applicable. Model diagnostics support the validity of the results since, with very few exceptions (RESET statistics for the whole population and men, and heteroscedasticity for men), all diagnostic tests are insignificant indicating that there is no autocorrelation, heteroscedasticity, misspecification and non-normality In

For peer review only - http://bmjopen.bmj.com/site/about/guidelines.xhtml

addition, all CUSUM and CUSUMQ tests all models indicate stability and absence of structural breaks ("Stabl., no str. break" in the results of CUSUM indicates that the model was found stable and the null hypothesis of no structural break was not rejected [same applies to "Stabl." as an output for CUSUMQ]).

For peer review only

Table 2. Short-term and long-term relationship of suicide rates with positive and negative changes in influenza death rates among the whole population, men, and women in 1918-1956 in Sweden

	Whole population		Men		Women	
	Coeff. (95% CI)	p-value	Coeff. (95% CI)	p-value	Coeff. (95% CI)	p-value
**Short-term coefficients**						
Influenza +	0.00002 (-0.00077 to 0.00082)	0.955	-0.00014 (-0.00102 to 0.00075)	0.755	0.00098 (-0.00068 to 0.00263)	0.237
Influenza –	0.00087 (-0.00643 to 0.00817)	0.809	-0.00132 (-0.01001 to 0.00741)	0.760	0.00434 (-0.00595 to 0.01463)	0.394
**Long-term coefficients**						
Influenza +	0.00011	0.994	-0.00490	0.802	0.01403	0.521
Influenza –	0.00103	0.936	0.00548	0.775	-0.01098	0.600
**Model diagnostics**						
Q-test for autocorrelation, χ^2	14.130	0.658	9.674	0.917	9.614	0.919
Heteroscedasticity, χ^2	0.939	0.333	0.768	0.381	5.042	0.025
Normality, χ^2	0.420	0.811	0.132	0.936	2.383	0.304
RESET, F-statistics	0.413	0.745	0.764	0.524	0.505	0.683
CUSUM	Stabl., no str. break	NA	Stabl., no str. break	NA	Stabl., no str. break	NA
CUSUMQ	Stabl.	NA	Stabl.	NA	Stabl.	NA
Adj. R^2	0.309	NA	0.345	NA	0.432	NA
$Wald_{SR}$, F-statistics	0.056	0.815	0.155	0.697	0.183	0.673
$Wald_{LR}$, F-statistics	1.555	0.222	0.217	0.645	4.479	0.044

Note: Signs as "+" and "–" denote the exposure variable being partitioned in positive and negative changes, respectively. Wald test for asymmetry in the short-term ($Wald_{SR}$) and long-term ($Wald_{LR}$) has a null hypothesis of no cointegration. Q-test for autocorrelation reports the results of Portmanteau test for white noise. Heteroscedasticity is measured by Breusch-Pagan/Cook-Weisberg test. Normality is measured by Jarque-Bera test. RESET statistics refers to the regression specification error tests. CUSUM and CUSUMQ refer to the tests of the cumulative sum of recursive residuals and their squares, respectively. "NA" denotes that a certain test parameter was not applicable. Model diagnostics support the validity of the results since, with one exception (heteroscedasticity for women), all diagnostic tests are insignificant indicating that ere is no autocorrelation, heteroscedasticity, misspecification and non-normality In addition, all CUSUM and CUSUMQ tests all models indicate

For peer review only - http://bmjopen.bmj.com/site/about/guidelines.xhtml

stability and absence of structural breaks ("Stabl., no str. break" in the results of CUSUM indicates that the model was found stable and the null hypothesis of no structural break was not rejected [same applies to "Stabl." as an output for CUSUMQ]).

For peer review only

Table 3. Short-term and long-term relationship of suicide rates with positive and negative changes in influenza death rates among the whole population, men, and women in 1957-1978 in Sweden

	Whole population		Men		Women	
	**Coeff. (95% CI)**	**p-value**	**Coeff. (95% CI)**	**p-value**	**Coeff. (95% CI)**	**p-value**
**Short-term coefficients**						
Influenza +	-0.00964 (-0.02252 to 0.00324)	0.883	-0.00876 (-0.02738 to 0.00985)	0.328	-0.00976 (-0.02033 to 0.00081)	0.068
Influenza –	0.00194 (-0.02614 to 0.03003)	0.130	-0.01531 (-0.04658 to 0.01596)	0.309	0.02155 (-0.00343 to 0.04653)	0.085
**Long-term coefficients**						
Influenza +	0.01255	0.618	-0.02659	0.541	0.04091	0.064
Influenza –	-0.01033	0.684	0.024538	0.582	-0.02870	0.184
**Model diagnostics**						
Q-test for autocorrelation, χ^2	5.662	0.773	3.790	0.925	5.243	0.813
Heteroscedasticity, χ^2	0.102	0.750	0.155	0.694	0.079	0.778
Normality, χ^2	1.569	0.456	1.627	0.443	1.237	0.537
RESET, F-statistics	5.839	0.014	3.348	0.064	1.089	0.398
CUSUM	Stabl., no str. break	NA	Stabl., no str. break	NA	Stabl., no str. break	NA
CUSUMQ	Stabl.	NA	Stabl.	NA	Stabl.	NA
Adj. R^2	0.246	NA	0.337	NA	0.371	NA
$Wald_{SR}$, F-statistics	2.903	0.112	0.536	0.477	7.132	0.019
$Wald_{LR}$, F-statistics	0.922	0.354	0.297	0.595	26.74	<0.001

Note: Signs as "+" and "–" denote the exposure variable being partitioned in positive and negative changes, respectively. Wald test for asymmetry in the short-term ($Wald_{SR}$) and long-term ($Wald_{LR}$) has a null hypothesis of no cointegration. Q-test for autocorrelation reports the results of Portmanteau test for white noise. Heteroscedasticity is measured by Breusch-Pagan/Cook-Weisberg test. Normality is measured by Jarque-Bera test. RESET statistics refers to the regression specification error tests. CUSUM and CUSUMQ refer to the tests of the cumulative sum of recursive residuals and their squares, respectively. "NA" denotes that a certain test parameter was not applicable. Model diagnostics support the validity of the results since, with one exception (RESET statistics for the whole population), all diagnostic tests are insignificant indicating that there is no autocorrelation, heteroscedasticity, misspecification and non-normality In addition, all CUSUM and CUSUMQ tests all

For peer review only - http://bmjopen.bmj.com/site/about/guidelines.xhtml

models indicate stability and absence of structural breaks (“Stabl., no str. break” in the results of CUSUM indicates that the model was found stable and the null hypothesis of no structural break was not rejected [same applies to “Stabl.” as an output for CUSUMQ]).

For peer review only

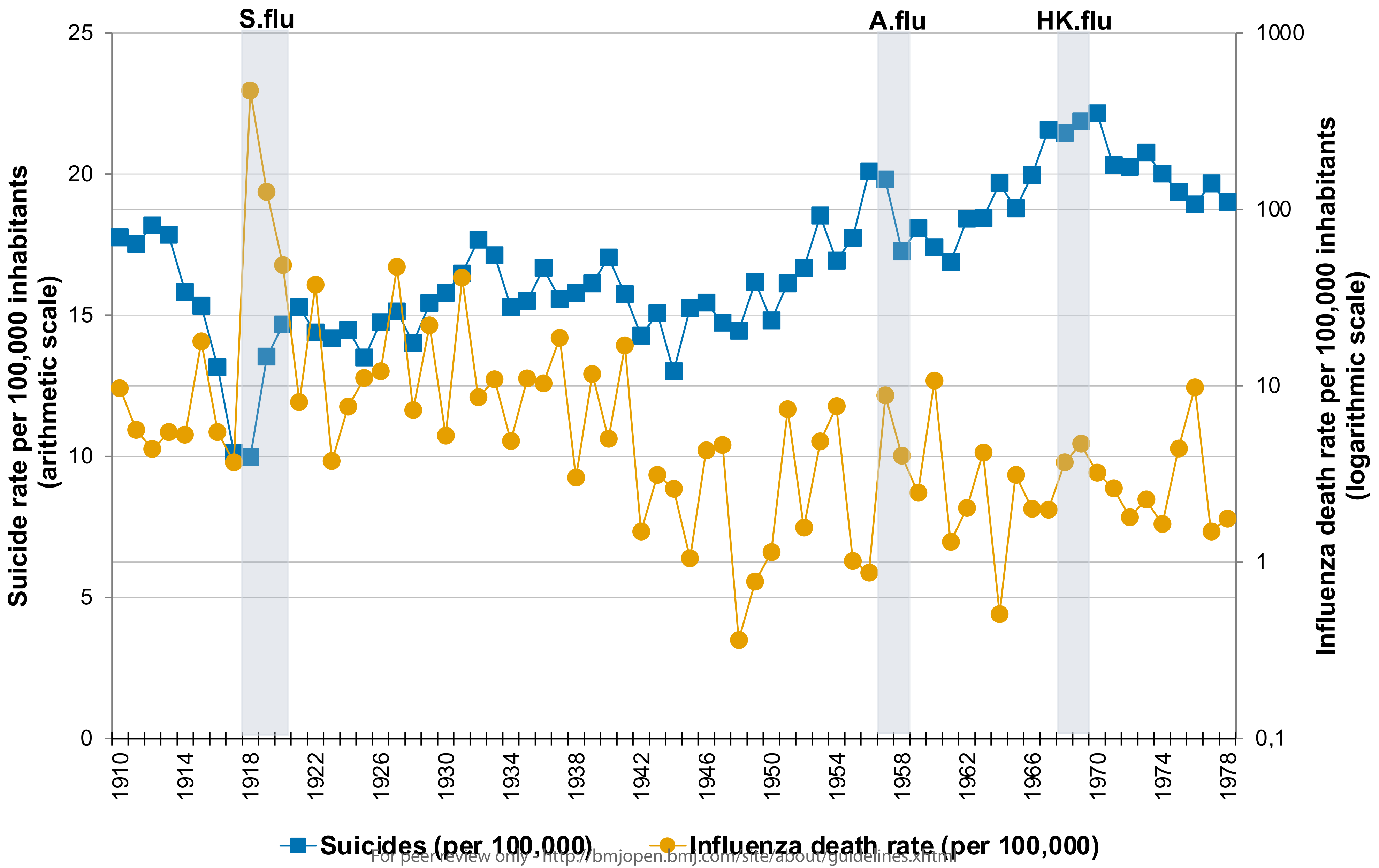

For peer review only - http://bmjopen.bmj.com/site/about/guidelines.xhtml

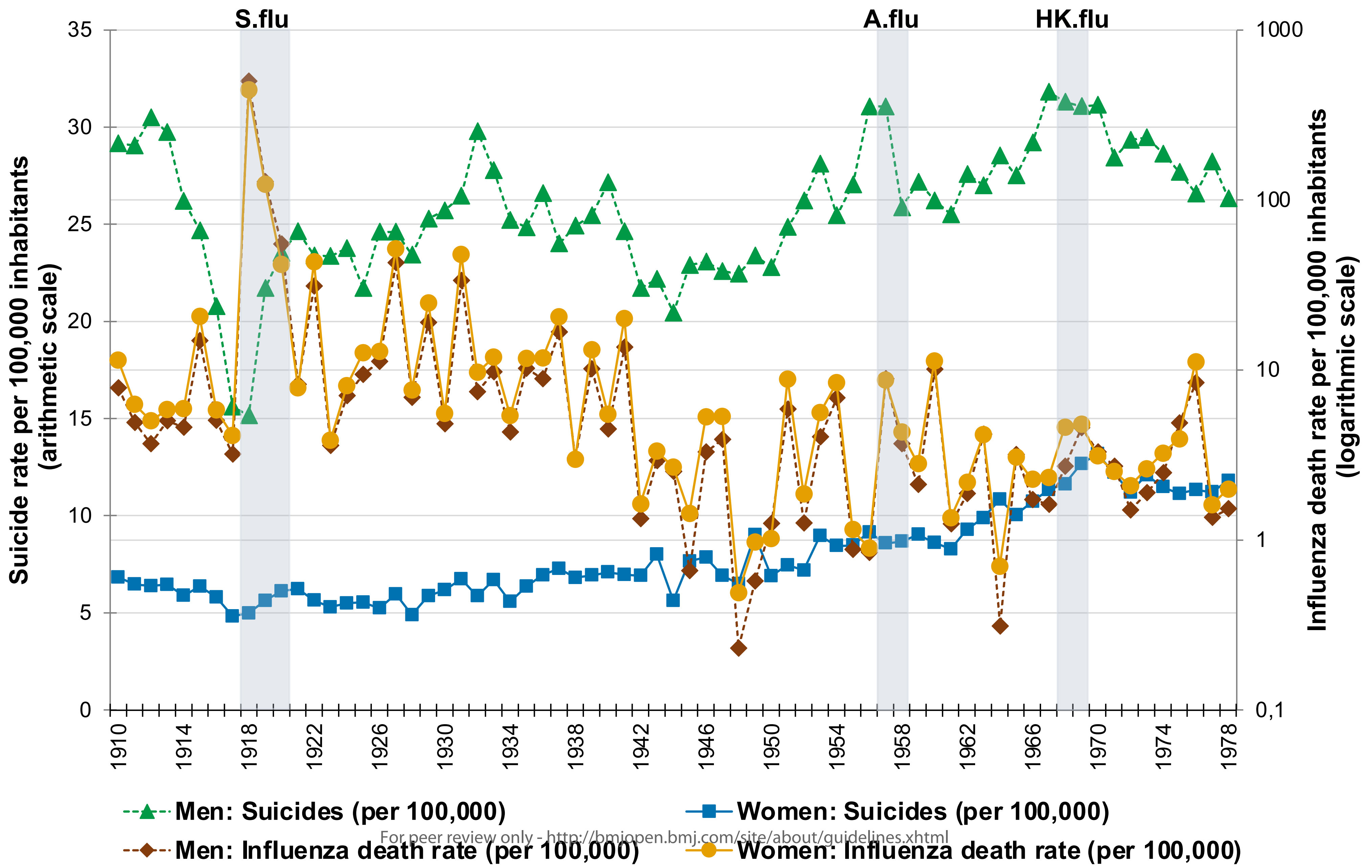

For peer review only - http://bmjopen.bmj.com/site/about/guidelines.xhtml

Supplementary material

eTable 1. Original data on the number of deaths by influenza and suicide, and population size for total population, men, and women in 1910-1978, retrieved from the Statistical Yearbook of Sweden from 1910-1978

Year	Total population, N	Men, N	Women, N	Deaths by influenza in total population, N	Deaths by influenza among men, N	Deaths by influenza among women, N	Deaths by suicide in total population, N	Deaths by suicide among men, N	Deaths by suicide among women, N
1910	5522403	2698729	2823674	534	212	322	980	787	193
1911	5561799	2718638	2843161	312	134	178	974	790	184
1912	5604192	2740737	2863455	245	101	144	1019	836	183
1913	5638583	2756946	2881637	307	139	168	1006	820	186
1914	5679607	2777447	2902160	300	128	172	899	728	171
1915	5712740	2794552	2918188	1018	417	601	876	690	186
1916	5757566	2817950	2939616	314	143	171	757	586	171
1917	5800847	2841554	2959293	213	91	122	587	444	143
1918	5813850	2849205	2964645	27379	14245	13134	580	432	148
1919	5847037	2868395	2978642	7341	3667	3674	791	623	168
1920	5904489	2898256	3006233	2853	1596	1257	866	682	184
1921	5954316	2925988	3028328	479	241	238	910	721	189
1922	5987520	2944031	3043489	2235	918	1317	861	689	172
1923	6005759	2948508	3057251	224	106	118	851	689	162
1924	6036118	2964230	3071888	458	210	248	874	705	169
1925	6053562	2972554	3081008	669	280	389	817	646	171
1926	6074368	2982625	3091743	731	335	396	896	734	162
1927	6087923	2990205	3097718	2875	1277	1598	921	736	185
1928	6105190	2999562	3105628	444	207	237	855	703	152
1929	6120080	3007946	3112134	1345	573	772	944	761	183
1930	6142191	3020848	3121343	319	146	173	970	777	193
1931	6162446	3037064	3125382	2518	1025	1493	1015	804	211
1932	6190364	3053528	3136836	531	228	303	1094	910	184
1933	6211566	3066888	3144678	673	300	373	1063	852	211
1934	6233090	3079690	3153400	303	133	170	953	777	176
1935	6250506	3090451	3160055	685	316	369	969	768	201
1936	6266888	3100534	3166354	646	275	371	1045	825	220
1937	6284722	3111256	3173466	1173	522	651	978	747	231
1938	6310214	3125000	3185214	190	95	95	996	779	217
1939	6341303	3142356	3198947	740	319	421	1022	800	222
1940	6371432	3160128	3211304	318	142	176	1086	858	228
1941	6406474	3180535	3225939	1082	434	648	1009	784	225
1942	6458200	3207756	3250444	96	43	53	922	697	225
1943	6522827	3240631	3282196	204	95	109	982	719	263
1944	6597348	3279723	3317625	172	83	89	858	671	187
1945	6673749	3321502	3352247	70	22	48	1018	761	257
1946	6763685	3366694	3396991	291	111	180	1044	777	267
1947	6842046	3407577	3434469	316	133	183	1008	770	238

For peer review only
For peer review only - http://bmjopen.bmj.com/site/about/guidelines.xhtml

Supplementary material

1948	6924888	3448122	3476766	25	8	17	1000	774	226
1949	6986181	3479079	3507102	54	20	34	1130	814	316
1950	7041829	3506442	3535387	80	44	36	1043	799	244
1951	7098740	3535736	3563004	522	208	314	1145	879	266
1952	7150606	3562475	3588131	112	45	67	1192	934	258
1953	7192316	3583598	3608718	347	145	202	1332	1008	324
1954	7234664	3605013	3629651	553	248	305	1225	918	307
1955	7290112	3633983	3656129	74	32	42	1293	983	310
1956	7338991	3659917	3679074	64	31	33	1474	1137	337
1957	7388611	3685654	3702957	649	327	322	1463	1145	318
1958	7429675	3706039	3723636	297	137	160	1282	959	323
1959	7462823	3722867	3739956	184	79	105	1350	1012	338
1960	7497967	3740119	3757848	801	378	423	1305	981	324
1961	7542028	3763040	3778988	98	47	51	1273	960	313
1962	7581148	3782252	3798896	154	71	83	1396	1043	353
1963	7627507	3805699	3821808	319	160	159	1406	1028	378
1964	7695200	3840897	3854303	39	12	27	1514	1096	418
1965	7772506	3882473	3890033	243	124	119	1459	1068	391
1966	7843088	3919170	3923918	157	68	89	1566	1145	421
1967	7892774	3942223	3950551	156	64	92	1702	1254	448
1968	7934996	3961414	3973582	291	108	183	1702	1240	462

For peer review only

Supplementary material

Supplementary material. Model diagnostics

We carried out several types of model diagnostics, including Portmanteau test for white noise to check autocorrelation in residuals, Breusch-Pagan/Cook-Weisberg test for heteroscedasticity, Jarque-Bera test on normality, and the regression specification error tests that indicates whether there is a misspecification in the model. We also performed tests for stability of the models by using the cumulative sum of recursive residuals and their squares to test whether there is a structural break due to changes in regression coefficients over time.

Supplementary material. STATA codes for the non-linear autoregressive distributed lag (NARDL) modelling of association between between influenza death rates and suicide rates

```
clear
use "Z:\Influenza_deaths_suicides\ALL19101978.dta"


///// 1. DATA MANAGEMENT: generate and label variables to be used in the analyses


/// Labels for original variables (retrieved from the Statistical Yearbooks 1910-1978)

 lab var year "Years 1910-1978"

 lab var number_population_all "Total number of inhabitants (population size) in Sweden in corresponding year"
 lab var number_population_men "Total number of male population in Sweden in corresponding year"
 lab var number_population_women "Total number of female population in Sweden in corresponding year"

 lab var number_influenza_all "Number of deaths from influenza in total population in corresponding year"
 lab var number_influenza_men "Number of deaths from influenza among men in corresponding year"
 lab var number_influenza_women "Number of deaths from influenza among women in corresponding year"

 lab var number_suicide_all "Number of suicides in total population in corresponding year"
 lab var number_suicide_men "Number of suicides among men in corresponding year"
 lab var number_suicide_women "Number of suicides among women in corresponding year"



/// Calculation of annual influenza death rates and suicide rates per 100,000 for total population, men, and
women

 gen influenza_rates_all = (number_influenza_all/number_population_all)*100000
 gen influenza_rates_men = (number_influenza_men/number_population_men)*100000
 gen influenza_rates_women = (number_influenza_women/number_population_women)*100000

 gen suicide_rates_all = (number_suicide_all/number_population_all)*100000
 gen suicide_rates_men = (number_suicide_men/number_population_men)*100000
 gen suicide_rates_women = (number_suicide_women/number_population_women)*100000

 lab var influenza_rates_all "Influenza death rates for total population per 100,000, annual"
 lab var influenza_rates_men "Influenza death rates among men per 100,000, annual"
 lab var influenza_rates_women "Influenza death rates among women per 100,000, annual"

 lab var suicide_rates_all "Suicide rates for total population per 100,000, annual"
 lab var suicide_rates_men "Suicide rates among men per 100,000, annual"
 lab var suicide_rates_women "Suicide rates among women per 100,000, annual"



/// Logarithmic transformation for suicide rates for total population, men, and women
```


For peer review only

Supplementary material

```
gen ln_suicide_rates_all = ln(suicide_rates_all)
gen ln_suicide_rates_men = ln(suicide_rates_men)
gen ln_suicide_rates_women = ln(suicide_rates_women)

lab var ln_suicide_rates_all "Log-transformed suicide rates for total population per 100,000, annual"
lab var ln_suicide_rates_men "Log-transformed suicide rates among men per 100,000, annual"
lab var ln_suicide_rates_women "Log-transformed suicide rates among women per 100,000, annual"



/// Variables for changes in death registration in Sweden 1910-1978 (as dummy variables)

// based on the Bertillon criteria (prior to 1931)
gen registration_19101930=0
replace registration_19101930=1 if year<=1930
lab var registration_19101930 "Dummy variable for death registration in 1910-1930 (Bertillion)"

// introduced in cooperation with other Nordic countries (1931-1950)
gen registration_19311950=0
replace registration_19311950=1 if year>=1931 & year<=1950
lab var registration_19311950 "Dummy variable for death registration in 1931-1950 (new registration)"

// ICD-6 (1951-1957)
gen registration_19511957=0
replace registration_19511957=1 if year>=1951 & year<=1957
lab var registration_19511957 "Dummy variable for death registration in 1951-1957 (ICD-6)"

// ICD-7 (1958-1968)
gen registration_19581968=0
replace registration_19581968=1 if year>=1958 & year<=1968
lab var registration_19581968 "Dummy variable for death registration in 1958-1968 (ICD-7)"

// ICD-8 (1969-1978)
gen registration_1969after=0
replace registration_1969after=1 if year>=1969
lab var registration_1969after "Dummy variable for death registration in 1969 and after (ICD-8)"
////////////////////////////////////////////////////////////////////////////////



///// 2. CHECKING VARIABLES' PROPERTIES AND TESTING THE CONDITIONS FOR MODELLING


/// Declare data to be time-series data
tsset year, yearly


/// Obtain optimal lags for each variable (for (i) augmentation in ADF and KPSS tests, and (ii) for p and q
parameters in NARDL).
/// Lags obtained for influenza death rates and logarithmically-transformed suicide rates for total population,
men, and women, and for time periods 1910-1978, 1918-1956, and 1957-1978
/// If AIC, HQIC, and SBIC information criteria indicated different lag orders, SBIC was used to select optimal
lags

varsoc influenza_rates_all
varsoc influenza_rates_all if tin(1918, 1956)
varsoc influenza_rates_all if tin(1957, )

varsoc influenza_rates_men
```


For peer review only - http://bmjopen.bmj.com/site/about/guidelines.xhtml

Supplementary material

```
varsoc influenza_rates_men if tin(1918, 1956)
varsoc influenza_rates_men if tin(1957, )

varsoc influenza_rates_women
varsoc influenza_rates_women if tin(1918, 1956)
varsoc influenza_rates_women if tin(1957, )

varsoc ln_suicide_rates_all
varsoc ln_suicide_rates_all if tin(1918, 1956)
varsoc ln_suicide_rates_all if tin(1957, )

varsoc ln_suicide_rates_men
varsoc ln_suicide_rates_men if tin(1918, 1956)
varsoc ln_suicide_rates_men if tin(1957, )

varsoc ln_suicide_rates_women
varsoc ln_suicide_rates_women if tin(1918, 1956)
varsoc ln_suicide_rates_women if tin(1957, )


/// Tests for stationarity: ADF and KPSS for influenza death rates and logarithmically-transformed suicide rates variables for total population, men, and women
/// Augmentation by at least one lag was used (for suicide rates in women - by two lags according to SBIC in varsoc)

dfuller influenza_rates_all, lag(1)
dfuller influenza_rates_men, lag(1)
dfuller influenza_rates_women, lag(1)

dfuller ln_suicide_rates_all, lag(1)
dfuller ln_suicide_rates_men, lag(1)
dfuller ln_suicide_rates_women, lag(1)
dfuller ln_suicide_rates_women, lag(2)

dfuller D.influenza_rates_all, lag(1)
dfuller D.influenza_rates_men, lag(1)
dfuller D.influenza_rates_women, lag(1)

dfuller D.ln_suicide_rates_all, lag(1)
dfuller D.ln_suicide_rates_men, lag(1)
dfuller D.ln_suicide_rates_women, lag(1)
dfuller D.ln_suicide_rates_women, lag(2)

kpss influenza_rates_all, maxlag(1) notrend
kpss influenza_rates_men, maxlag(1) notrend
kpss influenza_rates_women, maxlag(1) notrend

kpss ln_suicide_rates_all, maxlag(1) notrend
kpss ln_suicide_rates_men, maxlag(1) notrend
kpss ln_suicide_rates_women, maxlag(2) notrend

kpss D.influenza_rates_all, maxlag(1) notrend
kpss D.influenza_rates_men, maxlag(1) notrend
kpss D.influenza_rates_women, maxlag(1) notrend

kpss D.ln_suicide_rates_all, maxlag(1) notrend
kpss D.ln_suicide_rates_men, maxlag(1) notrend
kpss D.ln_suicide_rates_women, maxlag(2) notrend
```


For peer review only

Supplementary material

```
///// 3. ESTIMATION OF NON-LINEAR AUTOREGRESSIVE DISTRIBUTED LAG (NARDL) MODELS:
for total population, men, and women, and for time periods 1910-1978, 1918-1956, and 1957-1978
/// The dependent and independent variables are indicated in levels.
/// The covariates (i.e., changes in death registration system 1910-1978) are included with the deterministic option, but these were only kept in the models if statistically significant.
/// The optimal number of lags for dependent and independent variables (p and q parameters, respectively).
/// Since p and q parameters refer to levels, one additional lag is added to p and q to get an optimal lag length in differences (p and q must be at least 2).
/// The model provides an output for a long-term cointegration bounds test and diagnostic tests.

nardl ln_suicide_rates_all influenza_rates_all, p(2) q(2) h(69) plot bootstrap(100) level(95) residuals /* used as a final model */
nardl ln_suicide_rates_all influenza_rates_all, p(2) q(2) deterministic(registration_19101930 registration_19311950 registration_19511957 registration_19581968 registration_1969after) h(69) plot bootstrap(100) level(95) residuals

nardl ln_suicide_rates_men influenza_rates_men, p(2) q(2) h(69) plot bootstrap(100) level(95) residuals /* used as a final model */
nardl ln_suicide_rates_men influenza_rates_men, p(2) q(2) deterministic(registration_19101930 registration_19311950 registration_19511957 registration_19581968 registration_1969after) h(69) plot bootstrap(100) level(95) residuals

nardl ln_suicide_rates_women influenza_rates_women, p(3) q(2) h(69) plot bootstrap(100) level(95) residuals /* used as a final model */
nardl ln_suicide_rates_women influenza_rates_women, p(3) q(2) deterministic(registration_19101930 registration_19311950 registration_19511957 registration_19581968 registration_1969after) h(69) plot bootstrap(100) level(95) residuals

nardl ln_suicide_rates_all influenza_rates_all if tin(1918,1956), p(2) q(2) h(39) plot bootstrap(50) level(95) residuals
nardl ln_suicide_rates_men influenza_rates_men if tin(1918,1956), p(2) q(2) h(39) plot bootstrap(50) level(95) residuals
nardl ln_suicide_rates_women influenza_rates_women if tin(1918,1956), p(5) q(2) h(39) plot bootstrap(50) level(95) residuals

nardl ln_suicide_rates_all influenza_rates_all if tin(1957, ), p(2) q(2) h(22) plot bootstrap(50) level(95) residuals
nardl ln_suicide_rates_men influenza_rates_men if tin(1957, ), p(2) q(2) h(22) plot bootstrap(50) level(95) residuals
nardl ln_suicide_rates_women influenza_rates_women if tin(1957, ), p(2) q(2) h(22) plot bootstrap(50) level(95) residuals
```

/* REFERENCES

For varsoc and dfuller:
StataCorp. Stata 15 Base Reference Manual. College Station, TX: Stata Press; 2017

For kpss:
Author: Christopher F Baum, Boston College, USA baum@@bc.edu
Kwiatkowski, D., Phillips, P.C.B., Schmidt, P. and Y. Shin. Testing the null hypothesis of stationarity against the alternative of a unit root: How sure are we that economic time series have a unit root? Journal of Econometrics, 54, 1992, 159-178.
Lee, D. and P. Schmidt. On the power of the KPSS test of stationarity against fractionally-integrated alternatives. Journal of Econometrics, 73, 1996, 285-302.
Schwert, G.W. Tests for Unit Roots: A Monte Carlo Investigation. Journal of Business and Economic Statistics, 7, 1989, 147-160.

For nardl:

For peer review only

Supplementary material

Author: Stata implementation by Marco Sunder (sunder@wifa.uni-leipzig.de). This version was created on 26jan2012.
Shin, Y., Yu, B., Greenwood-Nimmo, M. (2011): Modelling asymmetric cointegration and dynamic multipliers in a nonlinear ARDL framework. Working paper (version of November 2011), http://ssrn.com/abstract=1807745. */

end of do-file

For peer review only

For peer review only - http://bmjopen.bmj.com/site/about/guidelines.xhtml

Supplementary material

eTable 2. Unit root tests at the level and first difference by Augmented Dickey-Fuller (ADF) and Kwiatkowski-Phillips-Schmidt-Shin (KPSS) test statistics

	ADF at level		KPSS at level		Results for stationarity at the level	ADF at first difference		KPSS at first difference		Results for stationarity at the 1st difference	Order of integration
	Test statistic	p-value	Test statistic	p-value[1]		Test statistic	p-value	Test statistic	p-value[1]		
**Suicide rate (log-transformed)**											
All	-2.181	0.213	1.980	<0.001	nonstationary	-6.640	<0.001	0.068	>0.1	stationary	1
Males	-3.230	0.018	0.964	<0.001	nonstationary	-6.512	<0.001	0.053	>0.1	stationary	1
Females	-0.517	0.889	3.130	<0.001	nonstationary	-8.692	<0.001	0.104	>0.1	stationary	1
**Influenza death rate**											
All	-4.895	<0.001	0.461	>0.1	stationary	*-8.727*	*<0.001*	*0.016*	>0.1	*stationary*	0
Males	-4.856	<0.001	0.431	>0.1	stationary	*-8.642*	*<0.001*	*0.016*	>0.1	*stationary*	0
Females	-4.940	<0.001	0.491	>0.1	stationary	*-8.824*	*<0.001*	*0.015*	>0.1	*stationary*	0

Note: For the ADF test, the null hypothesis implies that the variable contains a unit root (the alternate hypothesis is that the variable is stationary), whereas for the KPSS test the null hypothesis implies that the variable is stationary (the alternate hypothesis is that there is a unit root). The results for ADF and KPSS tests for stationarity at first difference for influenza death rates are reported as explanatory (written in Italics) since the stationarity at the level has already been established (i.e., integrated of the order zero).

[1] KPSS test results do not indicate the exact p-value, but report the level of significance at which the null hypothesis is rejected (1%, 2.5%, 5%, or 10% significance level).

Abbreviations: ADF, Augmented Dickey–Fuller unit-root test; KPSS, Kwiatkowski-Phillips-Schmidt-Shin test for stationarity

For peer review only - http://bmjopen.bmj.com/site/about/guidelines.xhtml

Supplementary material

eTable 3. Full specification for non-linear autoregressive distributed lag models used for the analysis of short-term and long-term relationship of suicide rates with positive and negative changes in influenza death rates among the whole population, men, and women in 1910-1978 in Sweden

	Whole population		Men		Women	
	Coefficient (95% CI)	p-value	Coefficient (95% CI)	p-value	Coefficient (95% CI)	p-value
LnSuicide, lag 1	-0.08466 (-0.23940 to 0.07009)	0.278	-0.11457 (-0.30588 to 0.07674)	0.235	-0.04517 (-0.15832 to 0.06799)	0.427
Influenza +, lag 1	-0.00001 (-0.00761 to 0.00759)	0.998	-0.00150 (-0.01032 to 0.00732)	0.734	0.00508 (-0.00366 to 0.01382)	0.249
Influenza –, lag 1	-0.00018 (-0.00775 to 0.00739)	0.962	-0.00165 (-0.01044 to 0.00714)	0.708	0.00487 (-0.00383 to 0.01358)	0.267
ΔLnSuicide, lag 1	-0.14610 (-0.43113 to 0.13893)	0.309	0.029465 (-0.27204 to 0.33097)	0.846	-0.65184 (-0.91785 to -0.38582)	<0.001
ΔLnSuicide, lag 2	NA	NA	NA	NA	-0.30807 (-0.55722 to -0.05892)	0.016
ΔInfluenza +	0.00002 (-0.00036 to 0.00039)	0.931	0.00004 (-0.00034 to 0.00041)	0.854	-0.00007 (-0.00056 to 0.00041)	0.760
ΔInfluenza +, lag 1	0.00153 (-0.00285 to 0.00590)	0.488	0.00085 (-0.00347 to 0.00518)	0.694	0.00093 (-0.00482 to 0.00668)	0.747
ΔInfluenza –	0.00103 (-0.00579 to 0.00785)	0.764	-0.00192 (-0.01005 to 0.00621)	0.638	0.00780 (0.00015 to 0.01544)	0.046
ΔInfluenza –, lag 1	-0.00070 (-0.00236 to 0.00096)	0.401	-0.00038 (-0.00212 to 0.00136)	0.663	-0.000075 (-0.00289 to 0.00139)	0.488
**Long-term effect**						
Influenza +	-0.00012	0.998	-0.01314	0.745	0.11254	0.538
Influenza –	0.00211	0.962	0.01443	0.722	-0.10789	0.544
**Model diagnostics**						

For peer review only - http://bmjopen.bmj.com/site/about/guidelines.xhtml

Supplementary material

Q-test for autocorrelation, χ^2	40.160	0.125	35.260	0.273	31.850	0.424
Heteroscedasticity, χ^2	3.649	0.056	5.439	0.020	1.280	0.258
Normality, χ^2	2.826	0.243	2.237	0.327	2.372	0.305
RESET, F-statistics	4.656	0.006	5.473	0.023	1.013	0.394
CUSUM	Stable, no structural break	NA	Stable, no structural break	NA	Stable, no structural break	NA
CUSUMQ	Stable	NA	Stable	NA	Stable	NA
Adj. R^2	0.257	NA	0.300	NA	0.374	NA
$Wald_{SR}$, F-statistics	0.054	0.817	0.275	0.602	1.062	0.307
$Wald_{LR}$, F-statistics	1.206	0.277	1.176	0.283	0.771	0.384
**Cointegration test statistics**						
t_BDM	-1.095	NA	-1.199	NA	-0.799	NA
F_PSS	3.789	NA	3.735	NA	2.530	NA
**Critical values for F_PSS**						
5% critical values; I(0), I(1)	k=1: 5.055, 5.915<br>k=2: 3.947, 5.020	NA	k=1: 5.055, 5.915<br>k=2: 3.947, 5.020	NA	k=1: 5.055, 5.915<br>k=2: 3.947, 5.020	NA

Note: F_PSS denotes F-statistics for Pesaran/Shin/Smith bounds test with null hypothesis of no cointegration. Critical values are retrieved from Narayan PK (2005) for a sample size of n=70.[1] The null hypothesis of no cointegration is supported if F_PSS statistics is lower than 5% I(0) critical value; the null hypothesis is rejected if F_PSS statistics is greater than 5% I(1) critical value. We applied a conservative approach to considering the number of exposure variables (k), as suggested by Shin Y, et al (2014) [2] (since influenza death rates represent one exposure (k=1), while the analysis actually partitions the

For peer review only - http://bmjopen.bmj.com/site/about/guidelines.xhtml

Supplementary material

exposure to positive and negative changes (k=2)). In a given table, the results of F_PSS for the whole population, men, and women are lower than the reported 5% I(0) critical values that accepts a null hypothesis of no cointegration.

"LnSuicide" indicates that suicide rates were logarithmically-transformed. "Δ" signifies the series is differenced. Signs as "+" and "–" denote the exposure variable being partitioned in positive and negative changes, respectively. "NA" denotes that a certain test or test parameter is not applicable.

Number of lags used for each variable in the model are noted by "lag#". To select the optimal number of lags to be used for choosing p and q parameters for the NARDL (i.e., numbers of lags for dependent and independent variables, respectively), we applied a varsoc command in STATA using the minimal values of Akaike Information Criterion, Schwarz's Bayesian information criterion (SBIC), and the Hannan and Quinn information criterion information criteria. If information criteria indicated different lag orders, SBIC was used to select optimal lags. Thus, for the analysis of the whole population and men, both exposure and outcome time series were lagged once, while for analysis among women to outcome time series were lagged twice and exposure time series were lagged once.

Wald test for asymmetry in a short-term ($Wald_{SR}$) and long-term ($Wald_{LR}$) has a null hypothesis of no cointegration. Q-test for autocorrelation reports the results of Portmanteau test for white noise. Heteroscedasticity is measured by Breusch-Pagan/Cook-Weisberg test. Normality is measured by Jarque-Bera test. RESET statistics refers to the regression specification error tests. CUSUM and CUSUMQ refer to the tests of the cumulative sum of recursive residuals and their squares, respectively. "Stable, no structural break" in the results of CUSUM indicates that the model was found stable and the null hypothesis of no structural break was not rejected (same applies to "Stable" as an output for CUSUMQ).

For peer review only

Supplementary material

eTable 4. Full specification for non-linear autoregressive distributed lag models used for the analysis of short-term and long-term relationship of suicide rates with positive and negative changes in influenza death rates among the whole population, men, and women in 1918-1956 in Sweden

	Whole population		Men		Women	
	Coefficient (95% CI)	p-value	Coefficient (95% CI)	p-value	Coefficient (95% CI)	p-value
LnSuicide, lag 1	-0.32229 (-0.73472 to 0.09015)	0.121	-0.26417 (-0.60333 to 0.07492)	0.122	-0.36067 (-0.97384 to 0.25250)	0.238
Influenza +, lag 1	0.00003 (-0.00851 to 0.00858)	0.994	-0.00129 (-0.011384 to 0.00879)	0.795	0.00506 (-0.00682 to 0.01695)	0.390
Influenza –, lag 1	-0.00033 (-0.00869 to 0.00803)	0.936	-0.00145 (-0.01129 to 0.00840)	0.766	0.00396 (-0.00801 to 0.01593)	0.503
ΔLnSuicide, lag 1	-0.19387 (-0.59955 to 0.21181)	0.337	-0.03045 (-0.44243 to 0.38153)	0.881	-0.58189 (-1.19784 to 0.03406)	0.063
ΔLnSuicide, lag 2	NA	NA	NA	NA	-0.36148 (-0.91643 to 0.19346)	0.193
ΔLnSuicide, lag 3	NA	NA	NA	NA	-0.11520 (-0.61658 to 0.38617)	0.641
ΔLnSuicide, lag 4	NA	NA	NA	NA	0.11306 (-0.26409 to 0.49021)	0.544
ΔInfluenza +	0.00002 (-0.00077 to 0.00082)	0.955	-0.00014 (-0.00102 to 0.00075)	0.755	0.00098 (-0.00068 to 0.00263)	0.237
ΔInfluenza +, lag 1	0.00140 (-0.00347 to 0.00627)	0.561	0.00095 (-0.00389 to 0.00580)	0.691	-0.00063 (-0.00821 to 0.00696)	0.866
ΔInfluenza –	0.00087 (-0.00643 to 0.00817)	0.809	-0.00132 (-0.01001 to 0.00741)	0.760	0.00434 (-0.00595 to 0.01463)	0.394
ΔInfluenza –, lag 1	-0.00076 (-0.00256 to 0.00104)	0.396	-0.00044 (-0.00233 to 0.00145)	0.637	-0.00052 (-0.00328 to 0.00224)	0.704
**Long-term effect**						
Influenza +	0.00011	0.994	-0.00490	0.802	0.01403	0.521

For peer review only - http://bmjopen.bmj.com/site/about/guidelines.xhtml

Supplementary material

Influenza –	0.00103	0.936	0.00548	0.775	-0.01098	0.600
**Model diagnostics**						
Q-test for autocorrelation, χ^2	14.130	0.658	9.674	0.917	9.614	0.919
Heteroscedasticity, χ^2	0.939	0.333	0.768	0.381	5.042	0.025
Normality, χ^2	0.420	0.811	0.132	0.936	2.383	0.304
RESET, F-statistics	0.413	0.745	0.764	0.524	0.505	0.683
CUSUM	Stable, no structural break	NA	Stable, no structural break	NA	Stable, no structural break	NA
CUSUMQ	Stable	NA	Stable	NA	Stable	NA
Adj. R^2	0.309	NA	0.345	NA	0.432	NA
$Wald_{SR}$, F-statistics	0.056	0.815	0.155	0.697	0.183	0.673
$Wald_{LR}$, F-statistics	1.555	0.222	0.217	0.645	4.479	0.044
**Cointegration test statistics**						
t_BDM	-1.596	NA	-1.591	NA	-1.207	NA
F_PSS	1.009	NA	1.019	NA	1.328	NA
**Critical values for F_PSS**						
5% critical values; I(0) to I(1)	k=1: 5.260, 6.160<br>k=2: 4.133, 5.260	NA	k=1: 5.260, 6.160<br>k=2: 4.133, 5.260	NA	k=1: 5.260, 6.160<br>k=2: 4.133, 5.260	NA

For peer review only

For peer review only - http://bmjopen.bmj.com/site/about/guidelines.xhtml

Supplementary material

Note: F_PSS denotes F-statistics for Pesaran/Shin/Smith bounds test with null hypothesis of no cointegration. Critical values are retrieved from Narayan PK (2005) for a sample size of n=40 [1]. The null hypothesis of no cointegration is supported if F_PSS statistics is lower than 5% I(0) critical value; the null hypothesis is rejected if F_PSS statistics is greater than 5% I(1) critical value. We applied a conservative approach to considering the number of exposure variables (k), as suggested by Shin Y, et al (2014) [2] (since influenza death rates represent one exposure (k=1), while the analysis actually partitions the exposure to positive and negative changes (k=2)). In a given table, the results of F_PSS for the whole population, men, and women are lower than the reported 5% I(0) critical values that accepts a null hypothesis of no cointegration.

"LnSuicide" indicates that suicide rates were logarithmically-transformed. "Δ" signifies the series is differenced. Signs as "+" and "–" denote the exposure variable being partitioned in positive and negative changes, respectively. "NA" denotes that a certain test or test parameter is not applicable.

Number of lags used for each variable in the model are noted by "lag#". To select the optimal number of lags to be used for choosing p and q parameters for the NARDL (i.e., numbers of lags for dependent and independent variables, respectively), we applied a varsoc command in STATA using the minimal values of Akaike Information Criterion, Schwarz's Bayesian information criterion (SBIC), and the Hannan and Quinn information criterion information criteria. If information criteria indicated different lag orders, SBIC was used to select optimal lags. Thus, for the analysis of the whole population and men, both exposure and outcome time series were lagged once, while for analysis among women, outcome time series were lagged four times and exposure time series were lagged once.

Wald test for asymmetry in a short-term ($Wald_{SR}$) and long-term ($Wald_{LR}$) has a null hypothesis of no cointegration. Q-test for autocorrelation reports the results of Portmanteau test for white noise. Heteroscedasticity is measured by Breusch-Pagan/Cook-Weisberg test. Normality is measured by Jarque-Bera test. RESET statistics refers to the regression specification error tests. CUSUM and CUSUMQ refer to the tests of the cumulative sum of recursive residuals and their squares, respectively. "Stable, no structural break" in the results of CUSUM indicates that the model was found stable and the null hypothesis of no structural break was not rejected (same applies to "Stable" as an output for CUSUMQ).

For peer review only

Supplementary material

eTable 5. Full specification for non-linear autoregressive distributed lag models used for the analysis of short-term and long-term relationship of suicide rates with positive and negative changes in influenza death rates among the whole population, men, and women in 1957-1978 in Sweden

	Whole population		Men		Women	
	Coefficient (95% CI)	p-value	Coefficient (95% CI)	p-value	Coefficient (95% CI)	p-value
LnSuicide, lag 1	-0.64009 (-1.15104 to -0.12914)	0.018	-0.52238 (-1.11901 to 0.07425)	0.081	-0.66040 (-1.04522 to -0.27558)	0.003
Influenza +, lag 1	0.008034 (-0.02823 to 0.04429)	0.640	-0.01385 (-0.05324 to 0.02553)	0.461	0.02702 (-0.00364 to 0.05768)	0.079
Influenza –, lag 1	0.00661 (-0.02823 to 0.04429)	0.699	-0.01281 (-0.054045 to 0.02841)	0.514	0.01895 (-0.01066 to 0.04857)	0.190
ΔLnSuicide, lag 1	0.14293 (-0.41621 to 0.70206)	0.590	-0.04565 (-0.70111 to 0.60981)	0.883	0.16936 (-0.25995 to 0.59868)	0.409
ΔInfluenza +	-0.00964 (-0.02252 to 0.00324)	0.883	-0.00876 (-0.02738 to 0.00985)	0.328	-0.00976 (-0.02033 to 0.00081)	0.068
ΔInfluenza +, lag 1	-0.01999 (-0.04573 to 0.00574)	0.117	-0.01837 (-0.04871 to 0.01197)	0.214	-0.01298 (-0.04073 to 0.014772)	0.331
ΔInfluenza –	0.00194 (-0.02614 to 0.03003)	0.130	-0.01531 (-0.04658 to 0.01596)	0.309	0.02155 (-0.00343 to 0.04653)	0.085
ΔInfluenza –, lag 1	0.00864 (-0.00271 to 0.02000)	0.124	0.01033 (-0.00294 to 0.02359)	0.116	0.00585 (-0.00565 to 0.01735)	0.292
**Long-term effect**						
Influenza +	0.01255	0.618	-0.02659	0.541	0.04091	0.064
Influenza –	-0.01033	0.684	0.024538	0.582	-0.02870	0.184
**Model diagnostics**						
Q-test for autocorrelation, χ^2	5.662	0.773	3.790	0.925	5.243	0.813

For peer review only - http://bmjopen.bmj.com/site/about/guidelines.xhtml

Supplementary material

Heteroscedasticity, χ^2	0.102	0.750	0.155	0.694	0.079	0.778
Normality, χ^2	1.569	0.456	1.627	0.443	1.237	0.537
RESET, F-statistics	5.839	0.014	3.348	0.064	1.089	0.398
CUSUM	Stable, no structural break	NA	Stable, no structural break	NA	Stable, no structural break	NA
CUSUMQ	Stable	NA	Stable	NA	Stable	NA
Adj. R^2	0.246	NA	0.337	NA	0.371	NA
$Wald_{SR}$, F-statistics	2.903	0.112	0.536	0.477	7.132	0.019
$Wald_{LR}$, F-statistics	0.922	0.354	0.297	0.595	26.74	<0.001
**Cointegration test statistics**						
t_BDM	-2.706	NA	-1.891	NA	-3.707	NA
F_PSS	2.649	NA	2.075	NA	4.688	NA
**Critical values for F_PSS**						
5% critical values; I(0), I(1)	k=1: 5.395, 6.350<br>k=2: 4.267, 5.473	NA	k=1: 5.395, 6.350<br>k=2: 4.267, 5.473	NA	k=1: 5.395, 6.350<br>k=2: 4.267, 5.473	NA

Note: F_PSS denotes F-statistics for Pesaran/Shin/Smith bounds test with null hypothesis of no cointegration. Critical values are retrieved from Narayan PK (2005) for a sample size of n=30 [1]. The null hypothesis of no cointegration is supported if F_PSS statistics is lower than 5% I(0) critical value; the null hypothesis is rejected if F_PSS statistics is greater than 5% I(1) critical value. We applied a conservative approach to considering the number of exposure variables (k), as suggested by Shin Y, et al (2014) [2] (since influenza death rates represent one exposure (k=1), while the analysis actually partitions the exposure to positive and negative changes (k=2)). In a given table, the results of F_PSS for the whole population, men, and women are lower than the reported 5% I(0) critical values that accepts a null hypothesis of no cointegration.

For peer review only - http://bmjopen.bmj.com/site/about/guidelines.xhtml

Supplementary material

“LnSuicide” indicates that suicide rates were logarithmically-transformed. “Δ” signifies the series is differenced. Signs as “+” and “–” denote the exposure variable being partitioned in positive and negative changes, respectively. “NA” denotes that a certain test or test parameter is not applicable.

Number of lags used for each variable in the model are noted by “lag#”. To select the optimal number of lags to be used for choosing p and q parameters for the NARDL (i.e., numbers of lags for dependent and independent variables, respectively), we applied a varsoc command in STATA using the minimal values of Akaike Information Criterion, Schwarz’s Bayesian information criterion (SBIC), and the Hannan and Quinn information criterion information criteria. If information criteria indicated different lag orders, SBIC was used to select optimal lags. Thus, for the analysis of the whole population, men, and women, both exposure and outcome time series were lagged once.

Wald test for asymmetry in a short-term ($Wald_{SR}$) and long-term ($Wald_{LR}$) has a null hypothesis of no cointegration. Q-test for autocorrelation reports the results of Portmanteau test for white noise. Heteroscedasticity is measured by Breusch-Pagan/Cook-Weisberg test. Normality is measured by Jarque-Bera test. RESET statistics refers to the regression specification error tests. CUSUM and CUSUMQ refer to the tests of the cumulative sum of recursive residuals and their squares, respectively. “Stable, no structural break” in the results of CUSUM indicates that the model was found stable and the null hypothesis of no structural break was not rejected (same applies to “Stable” as an output for CUSUMQ).

REFERENCES

1 Narayan PK. The saving and investment nexus for China: evidence from cointegration tests. *Applied Economics* 2005; **37**(17): 1979–1990.

2 Shin Y, Yu B, Greenwood-Nimmo M. Modelling Asymmetric Cointegration and Dynamic Multipliers in a Nonlinear ARDL Framework. In: Sickles RC, Horrace WC, eds. Festschrift in Honor of Peter Schmidt: Econometric Methods and Applications. New York: Springer Science+Business Media, 2014: 281–314.

For peer review only - http://bmjopen.bmj.com/site/about/guidelines.xhtml

STROBE Statement—checklist of items that should be included in reports of observational studies

	Item No	Recommendation	Page No
**Title and abstract**	1	(*a*) Indicate the study's design with a commonly used term in the title or the abstract	1
		(*b*) Provide in the abstract an informative and balanced summary of what was done and what was found	2
**Introduction**			
Background/rationale	2	Explain the scientific background and rationale for the investigation being reported	3
Objectives	3	State specific objectives, including any prespecified hypotheses	3
**Methods**			
Study design	4	Present key elements of study design early in the paper	3-4
Setting	5	Describe the setting, locations, and relevant dates, including periods of recruitment, exposure, follow-up, and data collection	3-5
Participants	6	(*a*) *Cohort study*—Give the eligibility criteria, and the sources and methods of selection of participants. Describe methods of follow-up *Case-control study*—Give the eligibility criteria, and the sources and methods of case ascertainment and control selection. Give the rationale for the choice of cases and controls *Cross-sectional study*—Give the eligibility criteria, and the sources and methods of selection of participants	3-5
		(*b*) *Cohort study*—For matched studies, give matching criteria and number of exposed and unexposed *Case-control study*—For matched studies, give matching criteria and the number of controls per case	n/a
Variables	7	Clearly define all outcomes, exposures, predictors, potential confounders, and effect modifiers. Give diagnostic criteria, if applicable	3-4
Data sources/ measurement	8*	For each variable of interest, give sources of data and details of methods of assessment (measurement). Describe comparability of assessment methods if there is more than one group	3-4
Bias	9	Describe any efforts to address potential sources of bias	4
Study size	10	Explain how the study size was arrived at	4
Quantitative variables	11	Explain how quantitative variables were handled in the analyses. If applicable, describe which groupings were chosen and why	5
Statistical methods	12	(*a*) Describe all statistical methods, including those used to control for confounding	5
		(*b*) Describe any methods used to examine subgroups and interactions	5
		(*c*) Explain how missing data were addressed	n/a
		(*d*) *Cohort study*—If applicable, explain how loss to follow-up was addressed *Case-control study*—If applicable, explain how matching of cases and controls was addressed *Cross-sectional study*—If applicable, describe analytical methods taking account of sampling strategy	n/a
		(*e*) Describe any sensitivity analyses	n/a

Continued on next page

For peer review only - http://bmjopen.bmj.com/site/about/guidelines.xhtml

**Results**			
Participants	13*	(a) Report numbers of individuals at each stage of study—eg numbers potentially eligible, examined for eligibility, confirmed eligible, included in the study, completing follow-up, and analysed	n/a
		(b) Give reasons for non-participation at each stage	n/a
		(c) Consider use of a flow diagram	n/a
Descriptive data	14*	(a) Give characteristics of study participants (eg demographic, clinical, social) and information on exposures and potential confounders	5-7
		(b) Indicate number of participants with missing data for each variable of interest	n/a
		(c) *Cohort study*—Summarise follow-up time (eg, average and total amount)	n/a
Outcome data	15*	*Cohort study*—Report numbers of outcome events or summary measures over time	6-7
		Case-control study—Report numbers in each exposure category, or summary measures of exposure	
		Cross-sectional study—Report numbers of outcome events or summary measures	
Main results	16	(*a*) Give unadjusted estimates and, if applicable, confounder-adjusted estimates and their precision (eg, 95% confidence interval). Make clear which confounders were adjusted for and why they were included	5-7, and 13-15
		(*b*) Report category boundaries when continuous variables were categorized	
		(*c*) If relevant, consider translating estimates of relative risk into absolute risk for a meaningful time period	
Other analyses	17	Report other analyses done—eg analyses of subgroups and interactions, and sensitivity analyses	
**Discussion**			
Key results	18	Summarise key results with reference to study objectives	7
Limitations	19	Discuss limitations of the study, taking into account sources of potential bias or imprecision. Discuss both direction and magnitude of any potential bias	8
Interpretation	20	Give a cautious overall interpretation of results considering objectives, limitations, multiplicity of analyses, results from similar studies, and other relevant evidence	7-8
Generalisability	21	Discuss the generalisability (external validity) of the study results	8
**Other information**			
Funding	22	Give the source of funding and the role of the funders for the present study and, if applicable, for the original study on which the present article is based	9

*Give information separately for cases and controls in case-control studies and, if applicable, for exposed and unexposed groups in cohort and cross-sectional studies.

Note: An Explanation and Elaboration article discusses each checklist item and gives methodological background and published examples of transparent reporting. The STROBE checklist is best used in conjunction with this article (freely available on the Web sites of PLoS Medicine at http://www.plosmedicine.org/, Annals of Internal Medicine at http://www.annals.org/, and Epidemiology at http://www.epidem.com/). Information on the STROBE Initiative is available at www.strobe-statement.org.

For peer review only
